# Hypothesis testing under uniform-block covariance structures

**DOI:** 10.1016/j.jmva.2026.105641

**Published:** 2026-04-20

**Authors:** Yifan Yang, Shuo Chen, Ming Wang

**Affiliations:** a Department of Population and Quantitative Health Sciences, Case Western Reserve University, Cleveland, OH, 44106, USA; b Department of Epidemiology and Public Health, University of Maryland, School of Medicine, Baltimore, MD, 21201, USA

**Keywords:** Block Hadamard product representation, Dimension reduction, High-dimensional covariance matrix, Uniform-block structure, primary 62H15, secondary 62H20

## Abstract

A block covariance structure is widely observed across large-scale and high-dimensional datasets in diverse fields such as biology, medicine, engineering, economics, and finance. This pattern entails partitioning a covariance matrix into uniform blocks, where each block exhibits equal variances and covariances. The importance of uniform-block structures lies in their ubiquity, interpretability, and ability to accommodate high dimensionality and data missingness. Despite their prevalence, statistical hypothesis testing under uniform-block covariance structures remains largely unexplored, and unknown statistical properties limit their application in research. To address this gap, we develop a comprehensive framework for hypothesis tests of both covariance structures and mean vectors, leveraging a novel block Hadamard product representation of uniform-block matrices. Specifically, we derive closed-form likelihood ratio test statistics and information statistics, explicitly establishing their null distributions. Additionally, we perform simultaneous marginal mean tests under a procedure that controls the false discovery proportion (FDP). Extensive simulations validate the consistency between theoretical and empirical distributions of the test statistics, assess the performance of the proposed FDP control procedure, and evaluate the robustness of the test statistics against structural disruptions, missing data, and distributional misspecification. Lastly, we apply our methodology to hypothesis testing in a high-dimensional imaging dataset.

## 1. Introduction

Covariance matrices with specific structures are fundamental to both the theoretical and practical aspects of multivariate analysis (Bilodeau and Brenner, 1999; Anderson, 2003; Muirhead, 2005). To address the challenges posed by large-scale and high-dimensional datasets resulting from technological advancements, various covariance structures have been developed. These customarily include bandability (Wu and Pourahmadi, 2003; Bickel and Levina, 2008a), sparsity (El Karoui, 2008; Bickel and Levina, 2008b; Cai and Liu, 2011), and hybrid structures that combine sparsity with low-rank properties (Fan et al., 2008, 2011). An alternative strategy for mitigating high dimensionality in covariance structures is the use of block structures. For example, Olkin (1973) introduced block circular symmetry structures. Szatrowski (1976) investigated covariance matrices with types I and II block compound symmetry structures, extending the compound symmetry framework of Votaw (1948). Many researchers have extensively studied covariance matrices with blocked compound symmetry or equicorrelation (Leiva, 2007; Roy and Leiva, 2011; Roy et al., 2015, 2016; Žežula et al., 2018). In particular, Liang et al. (2022) developed simultaneous tests for both mean and blocked compound symmetry covariance structures, and Filipiak et al. (2024) examined the problem of testing whether a block covariance structure belongs to a quadratic subspace. In this paper, we examine a block covariance structure in the context of high-dimensional hypothesis testing, termed the uniform-block structure, where each block contains identical block-wise diagonal and off-diagonal entries.

We focus on uniform-block structures because of their ubiquitous patterns, interpretability, and ability to handle high dimensionality. Firstly, uniform-block structures are important because they are prevalently observed in various high-throughput biomedical data, including genetics, proteomics, brain imaging, and RNA expression (Chen et al., 2018; Yang et al., 2024), as well as in large-scale engineering studies (Roustant and Deville, 2017; Roustant et al., 2020) and high-dimensional economic or financial data (Cadima et al., 2010; Archakov et al., 2024). Their widespread presence provides an opportunity to systematically investigate underlying scientific mechanisms within a unified framework. Secondly, the importance of uniform-block structures lies in their exceptional interpretability relative to classical block-diagonal structures. Specifically, a covariance structure with uniform blocks represents a form of variable clustering that frequently appears in real-world applications, where variables are grouped such that both within- and between-group correlations are groupwise identical. This offers more interpretability than the traditional clustering form, where between-group variables are assumed to be independent. Thirdly, uniform-block structures serve as a crucial link between existing covariance structures and high-dimensional statistical methods. As a direct generalization of sphericity and intraclass symmetry structures (Mauchly, 1940; Wilks, 1946) and a parallel version of blocked compound symmetry (Roy and Leiva, 2011; Roy et al., 2015, 2016), the parameterization of uniform-block structures depends on the number of blocks, significantly reducing the number of covariance parameters. If the model is correctly specified, this leads to more efficient statistical inferences in high-dimensional settings, with additional advantages such as closed-form covariance estimators and effective missing value management. These three aspects collectively highlight the importance of uniform-block structures in both theoretical research and real-world applications.

Although the uniform-block structures play an important role in multivariate analysis, they remain largely unexplored, likely due to the lack of appropriate algebraic tools. For example, while Geisser (1963) was the first to explore the null distributions of information statistics for population means under uniform-block covariance structures, rigorous proofs were not provided. Another challenge arises from the covariance estimation. Specifically, when the population covariance matrix follows a uniform-block structure, traditional marginal mean statistics do not exactly follow t-distributions. This deviation occurs because the diagonal estimators of the sample covariance matrix do not follow a scaled chi-square distribution, but rather a linear combination of two independent weighted chi-square distributions. Furthermore, when its dimension exceeds the sample size, the sample covariance matrix (multiplied by the sample size minus one) follows a singular Wishart distribution, lacking a probability density with respect to the Lebesgue measure (Díaz-García et al., 1997). These limitations have led to a paucity of research on uniform-block structures.

Historically, structures with uniform blocks have been known by various names across different research fields, but have not been comprehensively studied, particularly in statistical testing. Geisser (1963) referred to them as the uniform cases of certain orders and derived information statistics for testing population means of orders 1 and 2. Morrison (1972) later revisited Geisser’s information statistics for general orders but did not provide new findings. Huang and Yang (2010) investigated the random sampling issues involving uniform-block correlation matrices. Cadima et al. (2010) described them as group block structures and explored their eigendecomposition. Roustant and Deville (2017) introduced the term parametric block correlation matrices and provided necessary and sufficient conditions for positive definiteness. Roustant et al. (2020) investigated Gaussian process regression problems, referring to these matrices as generalized compound symmetry block covariance matrices. More recently, Archakov et al. (2024) examined uniform-block structured matrices, established their canonical forms, and called them block matrices with block partitions. Despite these contributions, comprehensive research on the algebraic properties of uniform-block matrices remains limited, restricting their broader application in high-dimensional statistical inference and fields such as biometrics, economics, and finance.

To bridge the gaps in high-dimensional statistical testing involving uniform-block structures, we propose likelihood ratio tests (LRTs) for population covariance structures, LRTs and Geisser’s information tests for population mean vectors, and a simultaneous multiple testing procedure for marginal mean components, all under uniform-block covariance structures. These tests allow the dimension of covariance matrices to exceed the sample size (except for the one-population covariance structure LRT). Our contributions are threefold. First, we introduce a novel block Hadamard product representation for uniform-block matrices, decomposing them into lower-dimensional diagonal and symmetric matrices. Compared to the original uniform-block matrices, these diagonal and symmetric matrices, along with a predetermined vector, serve as their unique “coordinate components”, remarkably reducing the computational complexity of matrix operations such as taking the inverse or computing the square. Second, leveraging this decomposition, we derive explicit expressions for LRT statistics and Geisser’s information statistics in both high-dimensional one- and multiple-population settings. Additionally, we rigorously establish the null distributions of Geisser’s information statistics in closed form. Third, we propose a multiple testing procedure that adjusts p-values for marginal mean tests, ensuring that the estimated false discovery proportion (FDP) remains below a predetermined significance level. Both the block Hadamard product representation and high-dimensional hypothesis tests involving uniform-block covariance structures will contribute to advancing the theoretical studies and the practical applications across various fields.

We structure the remainder of the paper as follows. Section 2 presents the definitions and fundamental properties of uniform-block matrices. In Section 3, we derive the LRT statistics and Geisser’s information statistics, and the FDP estimation procedure based on the theory of uniform-block matrices. We defer the simulation studies and the real data analysis to the supplementary material. There, the Simulation Studies section provides numerical demonstrations of the proposed procedures, while the Real Data Analysis section applies these tests to a high-dimensional imaging dataset comprising measurements of neurometabolites across various brain regions. Lastly, we summarize our findings and offer further remarks and discussions in Section 4. Throughout this paper, let ≜ denote the equality by definition. Let ⊤ denote the transpose of a vector or matrix. Let $0_{n\times m}$ and $1_{n\times m}$ denote the n by m all-zeros matrix and all-ones matrix, respectively. Additionally, let $I_{n}$ and $J_{n}≜1_{n\times n}$ denote the n by n identify and all-ones matrices. Let diag(⋅) and Bdiag(⋅) denote the diagonal matrix and the block-diagonal matrix. Let tr(⋅) and det(⋅) denote the trace and determinant of a square matrix, respectively. Let sum(⋅) denote the sum of all elements of a matrix. Let corr(Σ) denote the correlation matrix of the covariance matrix Σ. We denote N>0 if it is positive definite. Let $N_{p}(\mu ,\Sigma )$ denote the p-dimensional normal distribution with mean vector μ and covariance matrix Σ. Let $W_{p}(\Sigma ,n;\Omega )$ denote the noncentral Wishart distribution with p by p scale matrix Σ, degrees of freedom parameter n, and p by p noncentrality parameter Ω, respectively (see the appendix for further details on Ω). Detailed proofs of all lemmas and theorems are provided in the appendix.

## 2. Uniform-block matrix and uniform-block structure

In this section, we begin by defining a uniform-block matrix and its underlying structure. We then introduce the block Hadamard product representation for uniform-block matrices, revealing their algebraic properties.

### Definition 1 (*Partition-Size Vector and Partitioned Matrix by a Partition-Size Vector*).

Let $N\in R^{p\times p}$ be a real-valued matrix, and let $K\in Z_{+}$ be a positive integer such that K≤p.

- A column vector
$$p≜{\left(p_{1},\;\ldots \;,p_{K}\right)}^{⊤}\in Z_{+}^{K}$$
  is said to be a partition-size vector, if $p=p_{1}+\cdots +p_{K}$ and $p_{k}\geq 1$ for k∈{1,…,K}.
- The block matrix $\left(N_{kk^{\prime }}\right)$ is said to be the partitioned matrix of N (induced) by the partition-size vector $p≜{\left(p_{1},\;\ldots \;,p_{K}\right)}^{⊤}$, if the $\left(k,k^{\prime }\right)\text{th}$ block $N_{kk^{\prime }}$ is of dimension $p_{k}$ by $p_{k^{\prime }}$ for $k,k^{\prime }\in \{1,\;\ldots \;,K\}$.

### Definition 2 (*Uniform-Block Matrix and Structure*).

Let $N\in R^{p\times p}$ be a symmetric matrix, and let $\left(N_{kk^{\prime }}\right)$ be the partitioned matrix of N by partition-size vector $p≜{\left(p_{1},\;\ldots \;,p_{K}\right)}^{⊤}$.

- The block matrix $\left(N_{kk^{\prime }}\right)$ is said to be a uniform-block matrix, if there exist numbers $a_{kk}\in R$ and $b_{kk^{\prime }}\in R$, for $k,k^{\prime }\in \{1,\;\ldots \;,K\}$, satisfying that
$$N_{kk}=a_{kk}I_{p_{k}}+b_{kk}J_{p_{k}},\;k=k^{\prime };\;N_{kk^{\prime }}=b_{kk^{\prime }}1_{p_{k}\times p_{k^{\prime }}},\;b_{k^{\prime }k}=b_{kk^{\prime }},\;k\neq k^{\prime }.$$
  We further denote this uniform-block matrix as
$$N[A,B,p]≜\left(N_{kk^{\prime }}\right),\;\text{where}\;A≜diag\left(a_{11},\;\ldots \;,a_{KK}\right)\in R^{K\times K},\;B≜\left(b_{kk^{\prime }}\right)\in R^{K\times K}$$
  are diagonal and symmetric matrices, respectively.
- The structure of a uniform-block matrix N[A,B,p] is said to be a uniform-block structure.

Following Definition 2, we introduce two important examples of uniform-block matrices: the partitioned matrices of the identity matrix $I_{p}$ and the all-ones matrix $J_{p}$ by a given partition-size vector $p≜{\left(p_{1},\;\ldots \;,p_{K}\right)}^{⊤}$. For simplicity, we denote them as I[p] and J[p], instead of $I\left[I_{K},0_{K\times K},p\right]$ and $J\left[0_{K\times K},J_{K},p\right]$, throughout the paper, i.e.,

$$I[p]≜I\left[I_{K},0_{K\times K},p\right]=Bdiag\left(I_{p_{1}},\;\ldots \;,I_{p_{K}}\right)=I_{p},\;J[p]≜J\left[0_{K\times K},J_{K},p\right]=\left(1_{p_{k}\times p_{k^{\prime }}}\right)=J_{p}.$$

By Definition 2, we only know that a uniform-block matrix N[A,B,p] is “implicitly” determined by the matrices A, B, and the vector p. To make this relationship explicit, we introduce the block Hadamard product representation for uniform-block matrices utilizing the notations I[p] and J[p].

### Lemma 1 (*Block Hadamard Product Representation*).

Let N[A,B,p] be a uniform-block matrix with diagonal matrix $A≜diag\left(a_{11},\;\ldots \;\right.,\left.a_{KK}\right)$, symmetric matrix $B≜\left(b_{kk^{\prime }}\right)$, and partition-size vector $p≜{\left(p_{1},\;\ldots \;,p_{K}\right)}^{⊤}$. Suppose $p_{k}>1$ for k∈{1,…,K}, then the following block Hadamard product representation is unique (with respect to A and B):

N[A,B,p]=A∘I[p]+B∘J[p],

where ◦ denotes the block Hadamard product satisfying that A∘I[p] is the block-diagonal matrix $Bdiag\left(a_{11}I_{p_{1}},\;\ldots \;,a_{KK}I_{p_{K}}\right)$ and B∘J[p] is the symmetric block matrix $\left(b_{kk^{\prime }}1_{p_{k}\times p_{k^{\prime }}}\right)$.

### Lemma 2 (*Properties of Uniform-Block Matrices*).

Let N[A,B,p], $N_{1}\left[A_{1},B_{1},p\right]$, and $N_{2}\left[A_{2},B_{2},p\right]$ be uniform-block matrices with diagonal matrices A, $A_{1}$, and $A_{2}$, symmetric matrices B, $B_{1}$, and $B_{2}$, and the common partition-size vector $p≜{\left(p_{1},\;\ldots \;,p_{K}\right)}^{⊤}$ satisfying $p_{k}>1$ for k∈{1,…,K}. Throughout this paper, we denote

$$P≜diag\left(p_{1},\;\ldots \;,p_{K}\right)\in R^{K\times K},\;\Delta ≜A+BP\in R^{K\times K},\;\tilde{\Delta }≜A+P^{1/2}BP^{1/2}\in R^{K\times K}.$$

- (addition/subtraction) suppose $N^{*}=N_{1}\pm N_{2}$; then the partitioned matrix of $N^{*}$ by p is still a uniform-block matrix, denoted as $N^{*}[A^{*},B^{*},p]$, where $A^{*}=A_{1}\pm A_{2}$ and $B^{*}=B_{1}\pm B_{2}$.
- (multiplication) suppose $N^{*}=N_{1}N_{2}$; in general, the partitioned matrix of $N^{*}$ by p is not a uniform-block matrix. But if $N_{1}$ and $N_{2}$ commute, i.e., $N_{1}N_{2}=N_{2}N_{1}$, then it is still a uniform-block matrix, denoted as $N^{*}[A^{*},B^{*},p]$, where $A^{*}=A_{1}A_{2}=A^{*,⊤}$ and $B^{*}=A_{1}B_{2}+B_{1}A_{2}+B_{1}{PB}_{2}=B^{*,⊤}$.
- (power) suppose $N^{*}=N^{m}$ with integer m≥2; then the partitioned matrix of $N^{*}$ by p is still a uniform-block matrix, denoted as $N^{*}\left[A^{\left[m\right]},B^{\left[m\right]},p\right]$, where $A^{\left[1\right]}=A,\;B^{\left[1\right]}=B,\;A^{\left[m^{\prime }\right]}=A^{\left[m^{\prime }-1\right]}A$ and $B^{\left[m^{\prime }\right]}=A^{\left[m^{\prime }-1\right]}B+B^{\left[m^{\prime }-1\right]}A+B^{\left[m^{\prime }-1\right]}PB$ for $m^{\prime }\in \{2,\;\ldots \;,m\}$. In particular, when m=2, the square formulas are $A^{\left[2\right]}=A^{2}$ and $B^{\left[2\right]}=AB+BA+BPB$.
- (eigenvalues) N[A,B,p] has p real-valued eigenvalues in total; these are $a_{kk}$ with multiplicity $\left(p_{k}-1\right)$ for k∈{1,…,K}, and the remaining K eigenvalues are identical to those of Δ or $\tilde{\Delta }$.
- (determinant) N[A,B,p] has the determinant $\left(\prod_{k=1}^{K}a_{kk}^{p_{k}-1}\right)\;det\;(\Delta )$ or $\left(\prod_{k=1}^{K}a_{kk}^{p_{k}-1}\right)\;det\;(\tilde{\Delta })$.
- (inversion) suppose N is invertible and $N^{*}=N^{-1}$; then the partitioned matrix of $N^{*}$ by p is still a uniform-block matrix, denoted as $N^{*}[A^{*},B^{*},p]$, where $A^{*}=A^{-1}$ and $B^{*}=-\Delta^{-1}{BA}^{-1}$.
- (canonical form) there exists an orthonormal matrix $\Gamma \in R^{p\times p}$ satisfying that the block-diagonal matrix $\Xi ≜\Gamma N[A,B,p]\Gamma^{⊤}\in R^{p\times p}$ is the canonical form of N[A,B,p], where

(1) Γ≜Bdiag1p111×p1,…,1pK11×pKBdiagH~p1,…,H~pK,Ξ=BdiagΔ~,a11Ip1−1,…,aKKIpK−1,

and ${\tilde{H}}_{p_{k}}\in R^{\left(p_{k}-1\right)\times p_{k}}$ is the submatrix of a standard Helmert matrix of order $p_{k}$ without the first row (Lancaster, 1965).

By the power property in Lemma 2, the collection of all uniform-block matrices with a common partition-size vector forms a quadratic subspace (Seely, 1971). Hypothesis tests for determining whether a block covariance structure belongs to a quadratic subspace have been discussed (Filipiak et al., 2024).

### Remark 1 (Δ, $\tilde{\Delta }$, *Positive Definiteness, and Canonical Form*).

Both K by K matrices Δ≜A+BP and $\tilde{\Delta }≜A+P^{1/2}BP^{1/2}$ play important roles in the analysis of uniform-block matrices. Specifically, Δ determines the inversion formula in Lemma 2, while $\tilde{\Delta }$ determines the canonical form in Lemma 2. In general, Δ is not symmetric, $\tilde{\Delta }$ is symmetric, and they share the same eigenvalues. It is therefore not surprising that the K by K non-symmetric matrix Δ still possesses K real-valued eigenvalues. This follows from the fact that $\Delta =\left(AP^{-1}+B\right)P$ is similar, by definition, to a real-valued symmetric matrix $P^{1/2}\left(AP^{-1}+B\right)P^{1/2}=\tilde{\Delta }$, which has K real-valued eigenvalues. Thus, N[A,B,p]>0 if and only if A>0 (i.e., $a_{kk}>0$ for k∈{1,…,K}) and Δ>0 (or $\tilde{\Delta }>0$). The construction of the orthonormal matrix Γ can be traced back to Theorem 3.1 in Szatrowski (1976) and is subsequently restated in Archakov et al. (2024). Mathematically, a positive definite covariance matrix can always be eigendecomposed using its strictly positive eigenvalues. Statistically, however, the corresponding orthonormal matrix, typically constructed from its normalized eigenvectors, may depend on the (unknown) covariance parameters. By contrast, the proposed orthonormal matrix Γ is constructed solely from $p_{1},\;\ldots \;,p_{K}$, and therefore involves no unknown parameters. Meanwhile, $\Xi ≜Bdiag\left(\tilde{\Delta },a_{11}I_{p_{1}-1},\;\ldots \;,a_{KK}I_{p_{K}-1}\right)$ has a block-diagonal structure and contains all unknown covariance parameters.

### Remark 2 (*Non-Symmetric Uniform-Block Matrices, Structures, and Their Properties*).

The formal definition of uniform-block matrices was introduced by Yang et al. (2024). In Definition 2, we assume uniform-block matrices and structures are symmetric because our primary focus in this paper is on covariance matrices. Nevertheless, a non-symmetric uniform-block matrix (and corresponding structure) can be defined by allowing B to be non-symmetric. For such non-symmetric uniform-block matrices, all formulas in Lemma 2 remain valid; furthermore, multiplication is applicable to general (non-symmetric) uniform-block matrices (rather than commuting ones), and the eigenvalues, in general, may be complex. Unless explicitly specified otherwise, we consistently define uniform-block matrices and structures as symmetric throughout this paper.

We summarize the advantages of employing the block Hadamard product representation for studying uniform-block matrices as follows. Firstly, this representation provides an explicit expression for N[A,B,p] in terms of A, B, and p. Secondly, the components A, B, and p serve as “coordinate components” that govern the key algebraic properties of N[A,B,p], including its powers, inverse (if it exists), eigenvalues, and canonical form. Thirdly, computations involving the smaller K by K matrices A, B, and P can replace computations on the larger p by p matrix N, especially when K is typically much smaller than p. This reduction in matrix size substantially improves computational efficiency. These advantages broaden the applicability of uniform-block matrices across various fields.

Before applying the theory of uniform-block matrices to hypothesis testing problems, we specify the relationships between a covariance matrix and its precision matrix, and correlation matrix.

### Lemma 3 (*Uniform-Block Covariance, Precision, and Correlation Matrices*).

Let Σ[A,B,p] be a uniform-block covariance matrix with diagonal matrix $A≜diag\left(a_{11},\;\ldots \;,a_{KK}\right)$, symmetric matrix $B≜\left(b_{kk^{\prime }}\right)$, and partition-size vector $p≜{\left(p_{1},\;\ldots \;,p_{K}\right)}^{⊤}$ satisfying $p_{k}>1$ for k∈{1,…,K}. Furthermore, suppose A>0 and Δ≜A+BP>0 with $P≜diag\left(p_{1},\;\ldots \;,p_{K}\right)$, therefore Σ[A,B,p]>0. Then, the partitioned matrices of the precision matrix $\Theta ≜\Sigma^{-1}$ and the correlation matrix Ψ≜corr(Σ) by p exist and are still uniform-block matrices, denoted as $\Theta \left[A_{\Theta },B_{\Theta },p\right]$ and $\Psi \left[A_{\Psi },B_{\Psi },p\right]$, respectively, where

(2) $$\left\{\begin{array}{l} A_{\Theta }=A^{-1}\\ B_{\Theta }=-\Delta^{-1}{BA}^{-1}, \end{array}\;\left\{\begin{array}{l} A_{\Psi }=C^{-1/2}{AC}^{-1/2}\\ B_{\Psi }=C^{-1/2}{BC}^{-1/2}, \end{array}\;C≜diag\left(c_{11},\;\ldots \;,c_{KK}\right),\;c_{kk}≜a_{kk}+b_{kk},\;k\in \{1,\;\ldots \;,K\}.\right.\right.$$

## 3. Hypothesis testing under uniform-block covariance structures

In this section, we consider the following three hypothesis testing scenarios under the uniform-block covariance structures. Given M≥1 populations, suppose we have p-dimensional normal vectors $X_{1}^{\left(m\right)},\;\ldots \;,X_{n^{\left(m\right)}}^{\left(m\right)}\overset{\text{i.i.d.}}{∼}N_{p}\left(\mu^{\left(m\right)},\Sigma^{\left(m\right)}\right)$ with $\Sigma^{\left(m\right)}>0$ for m∈{1,…,M}, where $n^{\left(m\right)}$ denote the sample size from the *m*th population.

### Scenario 1 (testing covariance structures).

We focus on the hypothesis for a single population to determine whether it follows a (unknown) uniform-block covariance structure, given an unspecified mean vector, i.e., M=1,

$$H_{1}:\Sigma^{\left(1\right)}=\Sigma^{\left(1\right)}\left[A^{\left(1\right)},B^{\left(1\right)},p\right],\;\text{given}\;\text{unspecified}\;\mu^{\left(1\right)};\;K_{1}:\Sigma^{\left(1\right)}\;\text{is}\;\text{unstructured,}\;\text{given}\;\text{unspecified}\;\mu^{\left(1\right)};$$

and the hypothesis for multiple populations to determine the equality of (unknown) uniform-block covariance structures, given unspecified mean vectors, i.e., M>1,

$$H_{3}:\Sigma^{\left(1\right)}=\cdots =\Sigma^{\left(M\right)}=\Sigma [A,B,p]\text{,}\;\text{given}\;\text{unspecified}\;\mu^{\left(m\right)},\;\text{and}\;\Sigma^{\left(m\right)}=\Sigma^{\left(m\right)}\left[A^{\left(m\right)},B^{\left(m\right)},p\right],m\in \{1,\;\ldots \;,M\};$$

$$K_{3}:\Sigma^{\left(m^{\prime }\right)}\neq \Sigma^{\left(m\right)}\;\text{for}\;\text{some}\;m^{\prime }\neq m,\;\text{given}\;\text{unspecified}\;\mu^{\left(m\right)},\;\text{and}\;\Sigma^{\left(m\right)}=\Sigma^{\left(m\right)}\left[A^{\left(m\right)},B^{\left(m\right)},p\right],m\in \{1,\;\ldots \;,M\}.$$

### Scenario 2 (testing mean vectors).

We focus on the hypothesis for a single population to determine whether the population mean vector equals a specified vector $\mu_{0}\in R^{p}$, given a (unknown) uniform-block covariance structure, i.e., M=1,

$$H_{2}:\mu^{\left(1\right)}=\mu_{0},\;\text{given}\;\Sigma^{\left(1\right)}=\Sigma^{\left(1\right)}\left[A^{\left(1\right)},B^{\left(1\right)},p\right];\;K_{2}:\mu^{\left(1\right)}\neq \mu_{0},\;\text{given}\;\Sigma^{\left(1\right)}=\Sigma^{\left(1\right)}\left[A^{\left(1\right)},B^{\left(1\right)},p\right];$$

and the hypothesis for multiple populations to determine the equality of (unknown) population mean vectors, given an equal (unknown) uniform-block covariance structure, i.e., M>1,

$$H_{4}:\mu^{\left(1\right)}=\cdots =\mu^{\left(M\right)},\;\text{given}\;\Sigma^{\left(1\right)}=\cdots =\Sigma^{\left(M\right)}=\Sigma \left[A,B,p\right];$$

$$K_{4}:\mu^{\left(m^{\prime }\right)}\neq \mu^{\left(m\right)}\;\text{for}\;\text{some}\;m^{\prime }\neq m,\;\text{given}\;\Sigma^{\left(1\right)}=\cdots =\Sigma^{\left(M\right)}=\Sigma [A,B,p].$$

### Scenario 3 (simultaneously testing marginal means).

We focus on the simultaneous hypotheses for a single population to determine whether individual mean effects $\mu_{j}^{\left(1\right)}$ in $\mu^{\left(1\right)}≜{\left(\mu_{1}^{\left(1\right)},\;\ldots \;,\mu_{p}^{\left(1\right)}\right)}^{⊤}$, j∈{1,…,p}, are null, given a (unknown) uniform-block covariance structure, i.e., M=1,

$$H_{5,j}\;:\mu_{j}^{\left(1\right)}=0,\;\text{given}\;\Sigma^{\left(1\right)}=\Sigma^{\left(1\right)}\left[A^{\left(1\right)},B^{\left(1\right)},p\right];\;K_{5,j}\;:\mu_{j}^{\left(1\right)}\neq 0,\;\text{given}\;\Sigma^{\left(1\right)}=\Sigma^{\left(1\right)}\left[A^{\left(1\right)},B^{\left(1\right)},p\right];\;j\in \{1,\;\ldots \;,p\}.$$

We assume that $A^{\left(m\right)}>0$, A>0, $\Delta^{\left(m\right)}≜A^{\left(m\right)}+B^{\left(m\right)}P>0$, and Δ≜A+BP>0, thereby ensuring that $\Sigma^{\left(m\right)}>0$ and Σ>0 for m∈{1,…,M}, where $A^{\left(m\right)}$ and A are diagonal matrices, $B^{\left(m\right)}$ and B are symmetric matrices, $p≜{\left(p_{1},\;\ldots \;,p_{K}\right)}^{⊤}$ is a common partition-size vector satisfying $p_{k}>1$ for k∈{1,…,K}, and $P≜diag\left(p_{1},\;\ldots \;,p_{K}\right)$.

Before proceeding with the calculation of the test statistics, we introduce the maximum likelihood estimators for the uniform-block covariance matrices.

### Maximum likelihood estimators.

For the *m*th population, we denote

$${\bar{X}}^{\left(m\right)}≜\left(X_{1}^{\left(m\right)}+\cdots +X_{n^{\left(m\right)}}^{\left(m\right)}\right)/n^{\left(m\right)},\;W^{\left(m\right)}≜\sum_{i=1}^{n^{\left(m\right)}}\left(X_{i}^{\left(m\right)}-{\bar{X}}^{\left(m\right)}\right){\left(X_{i}^{\left(m\right)}-{\bar{X}}^{\left(m\right)}\right)}^{⊤},\;S^{\left(m\right)}≜W^{\left(m\right)}/\left(n^{\left(m\right)}-1\right),$$

and partition $S^{\left(m\right)}≜\left(S_{kk^{\prime }}^{\left(m\right)}\right)$ by p, satisfying that the block $S_{kk^{\prime }}^{\left(m\right)}$ is of dimension $p_{k}$ by $p_{k^{\prime }}$ for $k,k^{\prime }\in \{1,\;\ldots \;,K\}$. Thus, if $n^{\left(m\right)}>K+(K+1)K/2$ (i.e., the sample size exceeds the number of unknown covariance parameters), the following maximum likelihood estimators, for $k,k^{\prime }\in \{1,\;\ldots \;,K\}$,

(3) $${\hat{a}}_{kk}^{\left(m\right)}=\frac{tr\left(S_{kk}^{\left(m\right)}\right)}{p_{k}}-{\hat{b}}_{kk}^{\left(m\right)},\;{\hat{b}}_{kk^{\prime }}^{\left(m\right)}=\left\{\begin{array}{ll} \frac{sum\;\left(S_{kk^{\prime }}^{\left(m\right)}\right)}{p_{k}p_{k^{\prime }}}, & k\neq k^{\prime }\\ \frac{sum\;\left(S_{kk^{\prime }}^{\left(m\right)}\right)\;-\;tr\;\left(S_{kk^{\prime }}^{\left(m\right)}\right)}{p_{k}\left(p_{k^{\prime }}\;-\;1\right)}, & k=k^{\prime }, \end{array}\;{\hat{A}}^{\left(m\right)}≜diag\left({\hat{a}}_{11}^{\left(m\right)},\;\ldots \;,{\hat{a}}_{KK}^{\left(m\right)}\right),\;{\hat{B}}^{\left(m\right)}≜\left({\hat{b}}_{kk^{\prime }}^{\left(m\right)}\right)\right.$$

with ${\hat{b}}_{kk^{\prime }}^{\left(m\right)}={\hat{b}}_{k^{\prime }k}^{\left(m\right)}$ for $k\neq k^{\prime }$, are the uniformly minimum-variance unbiased estimators (Yang et al., 2024) for $A^{\left(m\right)}$ and $B^{\left(m\right)}$ in $H_{1}$, $H_{2}$, $K_{2}$, $K_{3}$, $H_{5j}$, and $K_{5j}$ for j∈{1,…,p}. We assume that ${\hat{A}}^{\left(m\right)}$ and ${\hat{\Delta }}^{\left(m\right)}≜{\hat{A}}^{\left(m\right)}+{\hat{B}}^{\left(m\right)}P$ are positive definite with probability 1 for m∈{1,…,M}. Additionally, let

(4) $${\hat{A}}_{\Theta }^{\left(m\right)}={\hat{A}}^{(m),-1},\;{\hat{B}}_{\Theta }^{\left(m\right)}=-{\hat{\Delta }}^{(m),-1}{\hat{B}}^{\left(m\right)}{\hat{A}}^{(m),-1},\;m\in \{1,\;\ldots \;,M\}.$$

Then, according to Lemma 3, m∈{1,…,M},

$${\hat{\Sigma }}^{\left(m\right)}\left[{\hat{A}}^{\left(m\right)},{\hat{B}}^{\left(m\right)},p\right]={\hat{A}}^{\left(m\right)}\circ I[p]+{\hat{B}}^{\left(m\right)}\circ J[p],\;{\hat{\Theta }}^{\left(m\right)}\left[{\hat{A}}_{\Theta }^{\left(m\right)},{\hat{B}}_{\Theta }^{\left(m\right)},p\right]={\hat{A}}_{\Theta }^{\left(m\right)}\circ I[p]+{\hat{B}}_{\Theta }^{\left(m\right)}\circ J[p]$$

are the plug-in estimators for covariance matrices $\Sigma^{\left(m\right)}\left[A^{\left(m\right)},B^{\left(m\right)},p\right]$ and precision matrices $\Theta^{\left(m\right)}\left[A_{\Theta }^{\left(m\right)},B_{\Theta }^{\left(m\right)},p\right]$, respectively.

To define the pooled version of estimators, we denote

$$n≜n^{\left(1\right)}+\cdots +n^{\left(M\right)},\;f^{\left(m\right)}≜n^{\left(m\right)}/n,\;m\in \{1,\;\ldots \;,M\},\;S≜\left(W^{\left(1\right)}+\cdots +W^{\left(M\right)}\right)/(n-M).$$

We partition $S≜\left(S_{kk^{\prime }}\right)$ by p such that the block $S_{kk^{\prime }}$ is of dimension $p_{k}$ by $p_{k^{\prime }}$ for $k,k^{\prime }\in \{1,\;\ldots \;,K\}$. Replacing $S_{kk^{\prime }}^{\left(m\right)}$ in (3) with $S_{kk^{\prime }}$, we obtain

$${\overset{ˆ}{a}}_{kk}=\frac{tr\;\left(S_{kk}\right)}{p_{k}}-{\overset{ˆ}{b}}_{kk},\;{\overset{ˆ}{b}}_{kk^{\prime }}=\left\{\begin{array}{ll} \frac{sum\;\left(S_{kk^{\prime }}\right)}{p_{k}p_{k^{\prime }}}, & k\neq k^{\prime }\\ \frac{sum\;\left(S_{kk^{\prime }}\right)\;-\;tr\;\left(S_{kk^{\prime }}\right)}{p_{k}\left(p_{k^{\prime }}\;-\;1\right)}, & k=k^{\prime }, \end{array}\;\overset{ˆ}{A}≜diag\left({\overset{ˆ}{a}}_{11},\;\ldots \;,{\overset{ˆ}{a}}_{KK}\right),\;\overset{ˆ}{B}≜\left({\overset{ˆ}{b}}_{kk^{\prime }}\right)\right.$$

with ${\hat{b}}_{k^{\prime }k}={\hat{b}}_{kk^{\prime }}$ for $k\neq k^{\prime }$. If n>K+(K+1)K/2, they are the uniformly minimum-variance unbiased estimators for A and B in $H_{3}$, $H_{4}$, and $K_{4}$. We assume that $\hat{A}$ and $\hat{\Delta }≜\hat{A}+\hat{B}P$ are positive definite with probability 1, and denote

$${\hat{A}}_{\Theta }={\hat{A}}^{-1},\;{\hat{B}}_{\Theta }=-{\hat{\Delta }}^{-1}\hat{B}{\hat{A}}^{-1}.$$

Similarly,

$$\hat{\Sigma }[\hat{A},\hat{B},p]=\hat{A}\circ I[p]+\hat{B}\circ J[p],\;\hat{\Theta }\left[{\hat{A}}_{\Theta },{\hat{B}}_{\Theta },p\right]={\hat{A}}_{\Theta }\circ I[p]+{\hat{B}}_{\Theta }\circ J[p]$$

are the plug-in estimators for the covariance matrix Σ[A,B,p] and the precision matrix $\Theta \left[A_{\Theta },B_{\Theta },p\right]$, respectively.

### Remark 3 (*Alternative Approach for Maximum Likelihood Estimators*).

Apart from the maximum likelihood estimators in (3), obtained by the method of Yang et al. (2024), an alternative approach to derive them is through the canonical form in (1).

### 3.1. Hypothesis tests for covariance structures in Scenario 1

#### Theorem 1 (*LRT Statistics Related to Covariance Structures*).

- Suppose that n>p and $p_{k}>2$ for k∈{1,…,K}. Then, the LRT statistic $\Lambda_{1}$ for testing $H_{1}$ versus $K_{1}$, raised to the power of 2/n, is computed as
$$\Lambda_{1}^{2/n}=\left\{\prod_{k=1}^{K}{\left(p_{k}-1\right)}^{p_{k}-1}\right\}\left\{\frac{det\;\left(V^{\left(1\right)}\right)}{det\;\left(V_{00}^{\left(1\right)}\right)}\right\}{\left\{\prod_{k=1}^{K}tr\;{\left(V_{kk}^{\left(1\right)}\right)}^{p_{k}-1}\right\}}^{-1},$$
  where $V^{\left(1\right)}≜\Gamma W^{\left(1\right)}\Gamma^{⊤}=\left(V_{kk^{\prime }}^{\left(1\right)}\right)$ is partitioned by a (K+1)-dimensional vector ${\left(K,p_{1}-1,\;\ldots \;,p_{K}-1\right)}^{⊤}$ for $k,k^{\prime }\in \{0,\;1,\;\ldots \;,K\}$. The transformation matrix Γ is defined in (1). Under the null hypothesis $H_{1}$,
$$\Lambda_{1}\overset{H_{1}}{∼}det{\left(W\right)}^{n/2}/\left\{det{\left(W_{00}\right)}^{n/2}\prod_{k=1}^{K}tr{\left(W_{kk}/\left(p_{k}-1\right)\right)}^{\left(p_{k}-1\right)n/2}\right\},$$
  where $W∼W_{p}(\Xi^{\left(1\right)},n-1)$ and $W=\left(W_{kk^{\prime }}\right)$ is partitioned by a (K+1)-dimensional vector ${\left(K,p_{1}-1,\;\ldots \;,p_{K}-1\right)}^{⊤}$ for $k,k^{\prime }\in \{0,\;1,\;\ldots \;,K\}$. Here $\Xi^{\left(1\right)}≜\Gamma \Sigma^{\left(1\right)}\left[A^{\left(1\right)},B^{\left(1\right)},p\right]\Gamma^{⊤}\;is\;the\;canonical\;form\;of\;\Sigma^{\left(1\right)}\left[A^{\left(1\right)},B^{\left(1\right)},p\right]$ presented in $H_{1}$.
- Suppose that ${\hat{A}}^{\left(m\right)}>0,\;\hat{A}>0,\;{\hat{\tilde{\Delta }}}^{\left(m\right)}>0$, and $\hat{\tilde{\Delta }}>0$ hold with probability 1, and that $n^{\left(m\right)}>1$ for m∈{1,…,M} and $p_{k}>2$ for k∈{1,…,K}. Then, the LRT statistic $\Lambda_{3}$ for testing $H_{3}$ versus $K_{3}$, raised to the power of 2/n, is computed as

$$\Lambda_{3}^{2/n}={\left(\prod_{m=1}^{M}f^{(m),f^{\left(m\right)}}\right)}^{-p}\prod_{m=1}^{M}{\left[\frac{det\;\left(V_{00}^{\left(m\right)}\right)}{det\;\left(\sum_{m=1}^{M}V_{00}^{\left(m\right)}\right)}\prod_{k=1}^{K}{\left\{\frac{tr\;\left(V_{kk}^{\left(m\right)}\right)}{tr\;\left(\sum_{m=1}^{M}V_{kk}^{\left(m\right)}\right)}\right\}}^{p_{k}-1}\right]}^{f^{\left(m\right)}},$$

where $V^{\left(m\right)}≜\Gamma W^{\left(m\right)}\Gamma^{⊤}=\left(V_{kk^{\prime }}^{\left(m\right)}\right)$ is partitioned by a (K+1)-dimensional vector ${\left(K,p_{1}-1,\;\ldots \;,p_{K}-1\right)}^{⊤}$ for $k,k^{\prime }\in \{0,\;1,\;\ldots \;,K\}$. The transformation matrix Γ is defined in (1). Under the null hypothesis $H_{3}$,

$$\Lambda_{3}\overset{H_{3}}{∼}{\left(\prod_{m=1}^{M}f^{(m),f^{\left(m\right)}}\right)}^{-pn/2}\left\{\prod_{m=1}^{M}det\;{\left(D_{m}\right)}^{n^{\left(m\right)}/2}\right\}\prod_{k=1}^{K}\prod_{m=1}^{M}D_{m,k}^{n^{\left(m\right)}\left(p_{k}-1\right)/2},$$

where $\left(D_{1},\;\ldots \;,D_{M}\right)∼{Matrix–Dirichlet}_{K}(\alpha ;0)$, i.e., a K-dimensional matrix variate Dirichlet (type I) distribution (Gupta and Nagar, 1999, Definition 6.2.1) with parameter vector $(\alpha ;0)≜\left(\left(n^{\left(1\right)}-1\right)/2,\;\ldots \;,\left(n^{\left(m\right)}-1\right)/2;0\right)$ and matrix $\Xi ≜\Gamma \Sigma [A,B,p]\Gamma^{⊤}$, the canonical form of Σ[A,B,p] presented in $H_{3}$. Moreover, for k∈{1,…,K}, $\left(D_{1,k},\;\ldots \;,D_{M,k}\right)∼{Dirichlet}_{M}\left(\alpha_{k}\right)$, i.e., an M-dimensional Dirichlet distribution (Ng et al., 2011, Definition 2.1) with parameter vector $\alpha_{k}≜\left(p_{k}-1\right)\alpha$.

We note that $\Lambda_{1}$ in Theorem 1 follows a product of standard LRT statistics, i.e., $\Lambda_{1}=\prod_{k=0}^{K}\Lambda_{1}^{\left(k\right)}$, where

$$\Lambda_{1}^{\left(0\right)}≜{\left\{\frac{det\;(V)}{\prod_{k=0}^{K}det\;\left(V_{kk}\right)}\right\}}^{n/2},\Lambda_{1}^{\left(k\right)}≜{\left\{\frac{det\;\left(V_{kk}\right)}{tr\;{\left(V_{kk}/\left(p_{k}\;-\;1\right)\right)}^{p_{k}-1}}\right\}}^{n/2},\;k\in \{1,\;\ldots \;,K\},$$

are (in general) dependent LRT statistics. Specifically, $\Lambda_{1}^{\left(0\right)}$ is the LRT statistic for testing the independence of (K+1) sets of variables and $\Lambda_{1}^{\left(k\right)}$ is the LRT statistic for testing the sphericity of the *k*th diagonal block for k∈{1,…,K}.

#### Remark 4 (*High-Dimensionality in Scenario 1*).

The LRT statistic $\Lambda_{1}$ requires n>p to ensure that $V^{\left(1\right)}>0$ with probability 1. Under the null hypothesis, we provide a large-sample Box approximation for $\Lambda_{1}$ in Corollary 1, which tends to a central chi-square distribution with degrees of freedom parameter p(p+1)/2−K(K+3)/2 in Corollary 3 as n→∞ since ρ→1 and ω→0. Additionally, we present a high-dimensional normal approximation to its null distribution in Corollary 2, when both p and n go to infinity with $p/n\to y_{p}\in (0,\;1]$, where $y_{p}$ is a constant. In contrast, the LRT statistic $\Lambda_{3}$ does not impose this restriction n>p. Specifically, the second part of Theorem 1 remains valid even when $n^{\left(m\right)}<p$ and n<p, as long as K by K matrices ${\hat{A}}^{\left(m\right)}$, $\hat{A}$, ${\hat{\tilde{\Delta }}}^{\left(m\right)}$, and $\hat{\tilde{\Delta }}$ are positive definite with probability 1. We emphasize that when $n^{\left(m\right)}<p$ and n<p, $\left(n^{\left(m\right)}-1\right)S^{\left(m\right)}$ and (n−M)S follow singular Wishart distributions lacking probability density functions with respect to Lebesgue measure (Díaz-García et al., 1997).

#### Corollary 1 (Large-Sample Box Approximation for $\Lambda_{1}$).

Given the same assumptions in Theorem 1, under the null hypothesis, $-2\;log\;\Lambda_{1}$ is approximately distributed as a linear combination of independent central chi-square variates when the sample size is sufficiently large, i.e.,

$$Pr\left(-2\;log\;\Lambda_{1}\leq t\right)=(1-\omega )\;Pr\;\left(\chi_{1}^{2}\leq \rho t\right)+\omega \;Pr\;\left(\chi_{2}^{2}\leq \rho t\right)+O\left(n^{-3}\right),$$

where $\chi_{1}^{2}∼\chi^{2}(f)$ and $\chi_{2}^{2}∼\chi^{2}(f+4)$ are independently distributed. Here, f≜p(p+1)/2−K(K+3)/2; ρ is determined by

$$12(1-\rho )nf=2\left(p^{3}-K^{3}\right)+9\left(p^{2}-K^{2}\right)+5p-17K-4\sum_{k=1}^{K}{\left(p_{k}-1\right)}^{-1};$$

and ω is determined by

$$\begin{array}{ll} -48n^{2}\rho^{2}\omega & \;=-6\left\{\left(p^{2}-K^{2}\right)+(p-K)-2K\right\}\{(1-\rho )n-1{\}}^{2}+12\left\{2(1-\rho )nf-\left(p^{2}-K^{2}\right)-(p-K)+2K\right\}\{(1-\rho )n-1\}\\ \; & \;-\left(p^{4}-K^{4}\right)-2\left(p^{3}-K^{3}\right)+p^{2}-K^{2}+2(p-K). \end{array}$$

#### Corollary 2 (High-Dimensional Normal Approximation for $\Lambda_{1}$).

Assume that $p_{1},\;\ldots \;,p_{K}$ are free of the sample size n, but $K≜K_{n}$ and $p≜p_{n}$ are two series of positive integers depending on the sample size n such that $K_{n}+1<p_{n}+1<n$ for all n≥4, $p_{n}/n\to y_{p}\in (0,\;1]$, and $K_{n}/n\to y_{K}<y_{p}\in (0,\;1]$ as n→∞. Given the same assumptions in Theorem 1, under the null hypothesis, $\left\{\log\left(\Lambda_{1}^{\frac{2}{n}}\right)-\mu_{n}\right\}/\sigma_{n}\to N(0,\;1)$ in distribution as n→∞, where the mean and variance are given by

$$\mu_{n}≜-\left(p_{n}-K_{n}\right)-log\left(1-\frac{p_{n}}{n\;-\;1}\right)\left(n-p_{n}-\frac{3}{2}\right)+log\left(1-\frac{K_{n}}{n\;-\;1}\right)\left(n\;-\;K_{n}-\frac{3}{2}\right)+\frac{K_{n}}{n\;-\;1},$$

$$\sigma_{n}^{2}≜-2\left\{log\left(1-\frac{p_{n}}{n\;-\;1}\right)-log\left(1-\frac{K_{n}}{n\;-\;1}\right)+\frac{p_{n}\;-\;K_{n}}{n\;-\;1}\right\}.$$

#### Corollary 3 (Large-Sample Wilks Approximation for $\Lambda_{1}$ and $\Lambda_{3}$).

Given the same assumptions in Theorem 1, under the null hypothesis, $-2\;log\;\Lambda_{1}$ is approximately distributed as $\chi^{2}(p(p+1)/2-K(K+3)/2)$ and $-2\;log\;\Lambda_{3}$ is approximately distributed as $\chi^{2}((M-1)K(K+3)/2)$, when the sample size is sufficiently large.

### 3.2. Hypothesis tests for mean vectors in Scenario 2

#### Theorem 2 (*LRT Statistics Related to Mean Vectors*).

- Suppose that ${\hat{A}}^{\left(1\right)}>0$ and ${\hat{\tilde{\Delta }}}^{\left(1\right)}>0$ hold with probability 1, and that $p_{k}>2$ for k∈{1,…,K}. Then, the LRT statistic $\Lambda_{2}$ for testing $H_{2}$ versus $K_{2}$, raised to the power of 2/n, is computed as
$$\Lambda_{2}^{2/n}=\left\{\frac{det\;\left(V_{00}^{\left(1\right)}\right)}{det\;\left(V_{00}^{\left(1\right)}\;+\;H_{00}^{\left(1\right)}\right)}\right\}\prod_{k=1}^{K}{\left\{\frac{tr\;\left(V_{kk}^{\left(1\right)}\right)}{tr\;\left(V_{kk}^{\left(1\right)}\;+\;H_{kk}^{\left(1\right)}\right)}\right\}}^{p_{k}-1},$$
  where $V^{\left(1\right)}≜\Gamma W^{\left(1\right)}\Gamma^{⊤}=\left(V_{kk^{\prime }}^{\left(1\right)}\right)$, $H^{\left(1\right)}≜\left(Y^{\left(1\right)}-v_{0}\right){\left(Y^{\left(1\right)}-v_{0}\right)}^{⊤}=\left(H_{kk^{\prime }}^{\left(1\right)}\right)$, both are partitioned by a (K+1)-dimensional vector ${\left(K,p_{1}-1,\;\ldots \;,p_{K}-1\right)}^{⊤}$ for $k,k^{\prime }\in \{0,\;1,\;\ldots \;,K\}$. Here, $Y^{\left(1\right)}≜\sqrt{n}\Gamma {\bar{X}}^{\left(1\right)}$, $v_{0}≜\sqrt{n}\Gamma \mu_{0},\;and\;\Gamma$ is defined in (1). Under the alternative hypothesis $K_{2}$ with general $v_{0}$, let $v^{\left(1\right)}≜\sqrt{n}\Gamma \mu^{\left(1\right)}$ and define the noncentrality parameter $\Omega_{H}^{\left(II\right)}≜\Xi^{(1),-1}\left(v^{\left(1\right)}-v_{0}\right){\left(v^{\left(1\right)}-v_{0}\right)}^{⊤}=\left(\Omega_{H,kk^{\prime }}^{\left(II\right)}\right)$, partitioned by a (K+1)-dimensional vector ${\left(K,p_{1}-1,\;\ldots \;,p_{K}-1\right)}^{⊤}$ for $k,k^{\prime }\in \{0,\;1,\;\ldots \;,K\}$, where $\Xi^{\left(1\right)}≜\Gamma \Sigma^{\left(1\right)}\left[A^{\left(1\right)},B^{\left(1\right)},p\right]\Gamma^{⊤}\;is\;the\;canonical\;form\;of\;\Sigma^{\left(1\right)}\left[A^{\left(1\right)},B^{\left(1\right)},p\right]$ presented in $H_{2}$. Then,
$$\Lambda_{2}\overset{K_{2}}{∼}\lambda_{2}^{n/2}\prod_{k=1}^{K}\beta_{II,2,k}^{\left(p_{k}-1\right)n/2}∼\lambda_{2}^{n/2}\prod_{k=1}^{K}{\left(1+\frac{1}{n\;-\;1}F_{2,k}\right)}^{-\left(p_{k}-1\right)n/2},$$
  where $\lambda_{2}∼\Lambda \left(K;n\;-\;1,\;1;\Omega_{H,00}^{\left(II\right)}\right)$, i.e., the noncentral Wilks lambda distribution (Butler and Wood, 2004), $\beta_{II,2,k}∼{Beta}_{II}((n\;-\;1)\left(p_{k}-1\right)/2,\;\left(p_{k}-1\right)/2;tr\left(\Omega_{H,kk}^{(\text{II)}}\right))$ for k∈{1,…,K}, i.e., the noncentral type II beta distribution (Chattamvelli, 1995), and $F_{2,k}∼F(p_{k}-1,\;(n\;-\;1)\left(p_{k}-1\right);tr\left(\Omega_{H,kk}^{\left(II\right)}\right))$ for k∈{1,…,K}, i.e., the noncentral F distribution. Under the null hypothesis $H_{2}$, all noncentrality parameters vanish.
- Suppose that $\hat{A}>0,\;\hat{\tilde{\Delta }}>0$ hold with probability 1, and that n>M and $p_{k}>2$ for k∈{1,…,K}. Then, the LRT statistic $\Lambda_{4}$ for testing $H_{4}$ versus $K_{4}$, raised to the power of 2/n, is computed as

$$\Lambda_{4}^{2/n}=\left\{\frac{det\;\left(\sum_{m=1}^{M}V_{00}^{\left(m\right)}\right)}{det\;\left({\tilde{H}}_{00}\;+\;\sum_{m=1}^{M}V_{00}^{\left(m\right)}\right)}\right\}\prod_{k=1}^{K}{\left\{\frac{tr\;\left(\sum_{m=1}^{M}V_{kk}^{\left(m\right)}\right)}{tr\;\left({\tilde{H}}_{kk}\;+\;\sum_{m=1}^{M}V_{kk}^{\left(m\right)}\right)}\right\}}^{p_{k}-1},$$

where $V^{\left(m\right)}≜\Gamma W^{\left(m\right)}\Gamma^{⊤}=\left(V_{kk^{\prime }}^{\left(m\right)}\right)$ for m∈{1,…,M}, $\tilde{H}≜\sum_{m=1}^{M}\left(Y^{\left(m\right)}-\sqrt{n^{\left(m\right)}}\bar{Y}\right){\left(Y^{\left(m\right)}-\sqrt{n^{\left(m\right)}}\bar{Y}\right)}^{⊤}=\left({\tilde{H}}_{kk^{\prime }}\right)$, all are partitioned by a (K+1)-dimensional vector ${\left(K,p_{1}-1,\;\ldots \;,p_{K}-1\right)}^{⊤}$ for $k,k^{\prime }\in \{0,\;1,\;\ldots \;,K\}$. Here, $\bar{Y}≜\sum_{m=1}^{M}\sqrt{n^{\left(m\right)}}Y^{\left(m\right)}/n$, $Y^{\left(m\right)}≜\sqrt{n^{\left(m\right)}}\Gamma {\bar{X}}^{\left(m\right)}≜\sqrt{n^{\left(m\right)}}{\tilde{v}}^{\left(m\right)},\;and\;\Gamma$ is defined in (1). Under the alternative hypothesis $K_{4}$, the noncentrality parameter $\Omega_{H}^{\left(II\right)}=(M-1)\;\Xi^{-1}\left\{\sum_{m=1}^{M}n^{\left(m\right)}\left({\tilde{v}}^{\left(m\right)}-\bar{\tilde{v}}\right){\left({\tilde{v}}^{\left(m\right)}-\bar{\tilde{v}}\right)}^{⊤}\right\}=\left(\Omega_{H,kk^{\prime }}^{\left(II\right)}\right)$, partitioned by a (K+1)-dimensional vector ${\left(K,p_{1}-1,\;\ldots \;,p_{K}-1\right)}^{⊤}$ for $k,k^{\prime }\in \{0,\;1,\;\ldots \;,K\}$, where $\bar{\tilde{v}}≜\left(n^{\left(1\right)}{\tilde{v}}^{\left(1\right)}+\cdots +n^{\left(m\right)}{\tilde{v}}^{\left(m\right)}\right)/n$ is the pooled mean vector, and $\Xi =\Gamma \Sigma \;[A,B,p]\Gamma^{⊤}$ is the canonical form of Σ[A,B,p] presented in $H_{4}$. Then,

$$\Lambda_{4}\overset{K_{4}}{∼}\lambda_{4}^{n/2}\prod_{k=1}^{K}\beta_{II,4,k}^{\left(p_{k}-1\right)n/2}∼\lambda_{4}^{n/2}\prod_{k=1}^{K}{\left(1+\frac{M\;-\;1}{n\;-\;M}F_{4,k}\right)}^{-\left(p_{k}-1\right)n/2},$$

where $\lambda_{4}∼\Lambda \left(K;n-M,M-1;\Omega_{H,00}^{\left(II\right)}\right)$, i.e., the noncentral Wilks lambda distribution (Butler and Wood, 2004), $\beta_{II,k}∼{Beta}_{II}((n-M)\left(p_{k}-1\right)/2,(M-1)\left(p_{k}-1\right)/2;tr\left(\Omega_{H,kk}^{\left(II\right)}\right))$ for k∈{1,…,K}, i.e., the noncentral type II beta distribution (Chattamvelli, 1995), and $F_{4,k}∼F\left((M-1)\left(p_{k}-1\right),(n-M)\left(p_{k}-1\right);tr\left(\Omega_{H,kk}^{\left(II\right)}\right)\right)$ for k∈{1,…,K}, i.e., the noncentral F distribution. Under the null hypothesis $H_{4}$, all noncentrality parameters vanish.

#### Corollary 4 (Large-Sample Wilks Approximation for $\Lambda_{2}$ and $\Lambda_{4}$).

Given the same assumptions in Theorem 2, under the null hypothesis, $-2\;log\;\Lambda_{2}$ is approximately distributed as $\chi^{2}(p)$ and $-2\;log\;\Lambda_{4}$ is approximately distributed as $\chi^{2}((M-1)p)$, when the sample size is sufficiently large.

In the calculation of $\Lambda_{2}$ or $\Lambda_{4}$, we transform the matrix $W^{\left(m\right)}$ into the matrix $V^{\left(m\right)}$ using Γ. Consequently, if $W^{\left(m\right)}$ contains missing values, $\Lambda_{2}$ or $\Lambda_{4}$ requires a data imputation process. However, the following information statistics leverage the uniform-block structures, eliminating the need for imputation of missing data.

#### Theorem 3 (*Geisser’s Information Statistics Related to Mean Vectors*).

- Suppose that ${\hat{A}}^{\left(1\right)}>0$ and ${\hat{\Delta }}^{\left(1\right)}>0$ hold with probability 1, and that n>K and $p_{k}>1$, k∈{1,…,K}. Then, the Geisser’s information statistic $G_{2}$ for testing $H_{2}$ versus $K_{2}$ is computed as
$$G_{2}=n{\left({\bar{X}}^{\left(1\right)}-\mu_{0}\right)}^{⊤}{\overset{ˆ}{\Theta }}^{\left(1\right)}\left[{\overset{ˆ}{A}}_{\Theta }^{\left(1\right)},{\overset{ˆ}{B}}_{\Theta }^{\left(1\right)},p\right]\left({\bar{X}}^{\left(1\right)}-\mu_{0}\right).$$
  Under the alternative hypothesis $K_{2}$ with general $\mu_{0}$,
$$G_{2}\overset{K_{2}}{∼}\sum_{k=1}^{K}\left(p_{k}-1\right)F_{k}+T^{2},$$
  where $F_{k}∼F\left(p_{k}-1,(n\;-\;1)\left(p_{k}-1\right);\delta_{k}\right)$ for k∈{1,…,K}, i.e., the noncentral F distribution, and $T^{2}∼K(n\;-\;1)/(n-K)F\left(K,n-K;\delta_{K+1}\right)$, i.e., the noncentral Hotelling statistic. Partition $\mu^{\left(1\right)}={\left(\mu_{1}^{(1),⊤},\;\ldots \;,\mu_{K}^{(1),⊤}\right)}^{⊤}$ and $\mu_{0}={\left(\mu_{0,1}^{⊤},\;\ldots \;,\mu_{0,K}^{⊤}\right)}^{⊤}$ satisfying that $\mu_{k}^{\left(1\right)},\mu_{0,k}\in R^{p_{k}}$ for k∈{1,…,K}. The noncentrality parameters are given by $\delta_{k}≜{\left(\mu_{k}^{\left(1\right)}-\mu_{0,k}\right)}^{⊤}\left(a_{kk}^{-1}I_{p_{k}}-a_{kk}^{-1}p_{k}^{-1}J_{p_{k}}\right)\left(\mu_{k}^{\left(1\right)}-\mu_{0,k}\right)$ for k∈{1,…,K}, and $\delta_{K+1}≜n{\left(\mu^{\left(1\right)}-\mu_{0}\right)}^{⊤}C_{p}^{⊤}\left(P\Delta^{-1}\right)C_{p}\left(\mu^{\left(1\right)}-\mu_{0}\right)$, where $C_{p}≜Bdiag\left(p_{1}^{-1}1_{1\times p_{1}},\;\ldots \;,p_{K}^{-1}1_{1\times p_{K}}\right)$ is a K by p block-diagonal matrix. Under the null hypothesis $H_{2}$, all noncentrality parameters vanish.
- Suppose that $\hat{A}>0$ and $\hat{\Delta }>0$ hold with probability 1, and that n>M+K and $p_{k}>1$ for k∈{1,…,K}. Then, the Geisser’s information statistic $G_{4}$ for testing $H_{4}$ versus $K_{4}$ is computed as

$$G_{4}=\sum_{m=1}^{M}n^{\left(m\right)}{\left({\bar{X}}^{\left(m\right)}-\bar{X}\right)}^{⊤}\hat{\Theta }\left[{\hat{A}}_{\Theta },{\hat{B}}_{\Theta },p\right]\left({\bar{X}}^{\left(m\right)}-\bar{X}\right).$$

Under the alternative hypothesis $K_{4}$,

$$G_{4}\overset{K_{4}}{∼}\sum_{k=1}^{K}(M-1)\left(p_{k}-1\right)F_{k}+T_{0}^{2},$$

where $F_{k}∼F\left((M-1)\left(p_{k}-1\right),(n-M)\left(p_{k}-1\right);\delta_{k}\right)$ for k∈{1,…,K}, i.e., the noncentral F distributions, and $T_{0}^{2}∼(n-M)tr\left(W_{1}W_{2}^{-1}\right)$, i.e., the Hotelling-Lawley statistic, with $W_{1}∼W_{K}\left(\Delta P^{-1},M-1;\Omega_{Y}^{\left(I\right)}\right)$ and $W_{2}∼W_{K}\left(\Delta P^{-1},n-M\right)$ independently distributed. Let $\bar{\mu }≜\sum_{m=1}^{M}f^{\left(m\right)}\mu^{\left(m\right)},\;n≜{\left(n^{\left(1\right)},\;\ldots \;,n^{\left(m\right)}\right)}^{⊤}$, $N≜diag\left(n^{\left(1\right)},\;\ldots \;,n^{\left(m\right)}\right)$, and $E_{kk}$ denote the matrix with a 1 in the (k,k)th element and zeros elsewhere, for k∈{1,…,K}. The noncentrality parameters are given by $\delta_{k}≜{\left(\mu^{(1),⊤},\;\ldots \;,\mu^{(m),⊤}\right)}^{⊤}$ $\left[\left\{\left(N-n^{-1}nn^{⊤}\right)⊗E_{kk}\right\}⊗\left(a_{kk}^{-1}I_{p_{k}}-a_{kk}^{-1}p_{k}^{-1}J_{p_{k}}\right)\right]\left(\mu^{(1),⊤},\;\ldots \;,\mu^{(m),⊤}\right)\;$ for k∈{1,…,K}, and $\Omega_{Y}^{\left(I\right)}\;≜\;\sum_{m=1}^{M}n^{\left(m\right)}C_{p}\left(\mu^{\left(m\right)}-\bar{\mu }\right){\left(\mu^{\left(m\right)}-\bar{\mu }\right)}^{⊤}C_{p}^{⊤}$, where $C_{p}≜Bdiag\left(p_{1}^{-1}1_{1\times p_{1}},\;\ldots \;,p_{K}^{-1}1_{1\times p_{K}}\right)$ is a K by p block-diagonal matrix. Under the null hypothesis $H_{4}$, all noncentrality parameters vanish.

#### Corollary 5 (Large-Sample Geisser Approximation for $G_{2}$ and $G_{4}$).

Given the same assumptions in Theorem 3, under the null hypothesis, $G_{2}$ is approximately distributed as $\chi^{2}(p)$ and $G_{4}$ is approximately distributed as $\chi^{2}((M-1)p)$, when the sample size is sufficiently large.

When K=1 or K=2, Geisser (1963) introduced the above statistic for testing $H_{2}$ versus $K_{2}$, and derived its exact null distribution in closed form. However, for K>2, Geisser (1963) only outlined an algorithm for computing the information statistic, without providing formal proofs. Using the block Hadamard product representation, our proofs establish that Geisser’s null distributions can be expressed as a linear combination of mutually independent F-variates, further augmented by either a Hotelling’s $T^{2}$ statistic (when M=1) or by a Hotelling-Lawley $T_{0}^{2}$ statistic (when M>1).

#### Remark 5 (*Approximate Distributions of the Hotelling’s* $T_{0}^{2}$ *Statistic*).

The Hotelling-Lawley $T_{0}^{2}$ statistic, also known as the Hotelling-Lawley trace, is widely employed for testing the equality of multiple population means. However, it is intractable to derive its exact null or alternative distribution. We explore F-type distributions in the simulation studies and real data analysis as its approximation (McKeon, 1974; Betz, 1987).

#### Remark 6 (*handling missing data*).

Compared to the LRT statistics $\Lambda_{2}$ and $\Lambda_{4}$, the information statistics $G_{2}$ and $G_{4}$ rely solely on the estimates of $A^{\left(m\right)}$ and $B^{\left(m\right)}$, without requiring data transformations such as $V^{\left(m\right)}$. The expressions in (3) demonstrate that ${\hat{a}}_{kk}^{\left(m\right)}+{\hat{b}}_{kk}^{\left(m\right)}$ represents the average of the diagonal elements of $S_{kk}^{\left(m\right)}$, ${\hat{b}}_{kk}^{\left(m\right)}$ represents the average of the off-diagonal elements of $S_{kk}^{\left(m\right)}$, and ${\hat{b}}_{kk^{\prime }}^{\left(m\right)}$ represents the average of all elements of $S_{kk^{\prime }}^{\left(m\right)}$. While conventional sample covariance matrices may be undefined in the presence of missing values, the proposed estimators remain valid when omitting the missing values from $S^{\left(m\right)}$ (or S), as long as $S_{kk^{\prime }}^{\left(m\right)}$ (or $S_{kk^{\prime }}$) includes at least one non-missing entry.

#### Remark 7 (*High-Dimensionality in Scenario 2*).

Compared to classical LRT statistics and Hotelling’s $T^{2}$ statistic, the LRT statistics $\Lambda_{2}$ and $\Lambda_{4}$, as well as Geisser’s information statistics $G_{2}$ and $G_{4}$, are applicable even when $n^{\left(m\right)}<p$ and n<p, as long as ${\hat{A}}^{\left(m\right)}$, $\hat{A}$, ${\hat{\Delta }}^{\left(m\right)}$, and $\hat{\Delta }$ are positive definite with probability 1 (and n>max{M+K,K+(K+1)K/2}). When $n^{\left(m\right)}<p$ and n<p, we remark that $\left(n^{\left(m\right)}-1\right)\;S^{\left(m\right)}$ and (n−M)S follow singular Wishart distributions lacking probability density functions with respect to Lebesgue measure (Díaz-García et al., 1997).

### 3.3. Simultaneous hypothesis tests for individual means in Scenario 3

We first simplify our notation due to the single population. For ease of presentation, we suppress the superscript ^(1)^ throughout this section. Specifically, we consider $X_{1},\;\ldots \;,X_{n}\overset{\text{i.i.d.}}{∼}N_{p}(\mu ,\Sigma [A,B,p])$ with $\mu ≜{\left(\mu_{1},\;\ldots \;,\mu_{p}\right)}^{⊤}$, A>0 and Δ≜A+BP>0. The sample mean and the sample covariance matrix are written as $\bar{X}≜{\left({\overset{‾}{X}}_{1},\;\ldots \;,{\overset{‾}{X}}_{p}\right)}^{⊤}$ and S, respectively. Additionally, we define $C≜diag\left(c_{11},\;\ldots \;,c_{KK}\right)$ and $D≜Bdiag\left(\sqrt{c_{11}}I_{p_{1}},\;\ldots \;,\sqrt{c_{KK}}I_{p_{K}}\right)$, where $c_{kk}≜a_{kk}+b_{kk}$ for k∈{1,…,K}. Thus, D contains the square-rooted diagonal elements of Σ[A,B,p]. Using the formulas in (3) and omitting the superscript (m) since m=1, we denote the estimators of A, B, C, D, and Σ[A,B,p] as follows: $\hat{A}≜diag\left({\hat{a}}_{11},\;\ldots \;,{\hat{a}}_{KK}\right)$, $\hat{B}≜\left({\hat{b}}_{kk^{\prime }}\right)$, $\hat{C}≜diag\left({\hat{c}}_{11},\;\ldots \;,{\hat{c}}_{KK}\right)$, $\hat{D}≜Bdiag\left(\sqrt{{\hat{c}}_{11}}I_{p_{1}},\;\ldots \;,\sqrt{{\hat{c}}_{KK}}I_{p_{K}}\right)$, where ${\hat{c}}_{kk}≜{\hat{a}}_{kk}+{\hat{b}}_{kk}$ for k∈{1,…,K}, and $\hat{\Sigma }[\hat{A},\hat{B},p]=\hat{A}\circ I[p]+\hat{B}\circ J[p]$.

To conduct simultaneous tests for $H_{5,j}\;:\mu_{j}=0$ versus $K_{5,j}\;:\mu_{j}\neq 0$, j∈{1,…,p}, given unknown Σ[A,B,p], we consider the transformed data $\sqrt{n}{\hat{D}}^{-1}\bar{X}∼N_{p}(\tilde{\mu },\tilde{\Sigma })\approx N_{p}(\tilde{\mu },corr(\Sigma \;[A,B,p]))$, where $\tilde{\mu }≜\sqrt{n}{\hat{D}}^{-1}\mu$, $\tilde{\Sigma }≜{\hat{D}}^{-1}\Sigma [A,B,p]{\hat{D}}^{-1}$. The approximation ≈ holds because ${\hat{c}}_{kk}$ uniformly converges to $c_{kk}$ for k∈{1,…,K} under normality (Bickel and Levina, 2008a; Fan and Han, 2017). This implies that $\mu_{j}=0$ for all j is equivalent to ${\tilde{\mu }}_{j}=0$ for all j. Thus, we test ${\tilde{\mu }}_{j}=0$ for j∈{1,…,p} using the marginal test statistic vector

(5) $$T={\left(T_{1},\;\ldots \;,T_{p}\right)}^{⊤}≜\sqrt{n}{\hat{D}}^{-1}\bar{X},\;T_{j}≜\frac{\sqrt{n}{\overset{‾}{X}}_{j}}{\sqrt{{\overset{ˆ}{c}}_{kk}}},\;\text{where}\;k\in \{1,\;\ldots \;,K\}\;\text{satisfies}\;\text{that}\;{\overset{‾}{p}}_{k-1}+1\leq j\leq {\overset{‾}{p}}_{k}.$$

Here, we define that ${\overset{‾}{p}}_{k}≜p_{1}+\cdots +p_{k}$, k∈{1,…,K} and ${\overset{‾}{p}}_{0}≜0$. However, two challenges arise in multiple hypothesis testing under a uniform-block covariance structure.

Firstly, the marginal test statistic $T_{j}$ in (5) does not exactly follow a t-distribution with degrees of freedom parameter (n−1), because ${\hat{c}}_{kk}$ does not follow a chi-square distribution; instead, it follows a linear combination of two independent weighted chisquare distributions, i.e., $(n\;-\;1)p_{k}{\hat{c}}_{kk}∼\left(a_{kk}+p_{k}b_{kk}\right)\;\chi^{2}(n\;-\;1)+a_{kk}\;\chi^{2}\left((n\;-\;1)\left(p_{k}-1\right)\right)$. Therefore, applying the Welch-Satterthwaite method (Satterthwaite, 1946; Welch, 1947), we approximate $(n\;-\;1)p_{k}{\hat{c}}_{kk}\overset{\text{Welch-Satterthwaite}}{∼}d_{k}\chi^{2}\left(v_{k}\right)$, where $d_{k}≜\left(a_{kk}^{2}+p_{k}b_{kk}^{2}+\right.\left.2a_{kk}b_{kk}\right)/\left(a_{kk}+b_{kk}\right)$ and $v_{k}≜(n\;-\;1)p_{k}{\left(a_{kk}+b_{kk}\right)}^{2}/\left(a_{kk}^{2}+2a_{kk}b_{kk}+p_{k}b_{kk}^{2}\right)$ are obtained by matching their means and variances. This approximation remains valid in high-dimensional settings (Zhang et al., 2020): under regularity conditions, $(n\;-\;1)p_{k}{\hat{c}}_{kk}$ and $d_{k}\chi^{2}\left(v_{k}\right)$ have the same asymptotic distribution. Thus, under ${\tilde{\mu }}_{j}=0,T_{j}$ in (5) has the following approximate distribution: for j∈{1,…,p},

(6) $$T_{j}≜\sqrt{n}{\overset{‾}{X}}_{j}/\sqrt{{\overset{ˆ}{c}}_{kk}}\overset{\text{Welch-Satterthwaite}}{∼}t\left(v_{k}\right),\;v_{k}≜(n\;-\;1)p_{k}\;{\left(a_{kk}+b_{kk}\right)}^{2}/\left(a_{kk}^{2}+2a_{kk}b_{kk}+p_{k}b_{kk}^{2}\right),$$

where k∈{1,…,K} satisfies that ${\overset{‾}{p}}_{k-1}+1\leq j\leq {\overset{‾}{p}}_{k}$ and t(⋅) denotes the t-distribution. The (unadjusted) p-values of marginal tests are calculated as

(7) $$P_{j}≜2\Phi_{v_{k}}\left(-\left|T_{j}\right|\right),\;j\in \{1,\;\ldots \;,p\},$$

where $\Phi_{v_{k}}(\cdot )$ is the cumulative distribution function of $t\left(v_{k}\right)$.

Secondly, an appropriate measure for type I errors is required due to the dependence among marginal test statistics. Conventionally, when the test statistics are independent or their joint distribution exhibits positive regression dependence on subsets (Benjamini and Yekutieli, 2001), the standard Benjamini–Hochberg procedure can be applied to control the false discovery rate (FDR) (Benjamini and Hochberg, 1995). However, when the simultaneous test statistics are correlated, e.g., in the presence of uniform-block structure, the expectation and variance of the number of false discoveries can be strongly influenced by these non-negligible dependencies (Finner and Roters, 2002; Schwartzman and Lin, 2011). Thus, incorporating the dependence structure among test statistics is essential to avoid misleading statistical inferences, such as loss of statistical power and distortion in the ranking of p-values (Leek and Storey, 2008). In settings with strong correlations, Fan et al. (2012) and Fan and Han (2017) suggest directly estimating the false discovery proportion (FDP), which is more relevant to the present study. Unlike FDR, which provides an expectation-based measure of error rate, FDP offers a direct assessment of false discoveries, i.e., FDR=E(FDP(T)), where T is a test threshold. Below, we describe how to control FDP in our setting following the approach of Fan et al. (2012) and Fan and Han (2017).

Given a threshold value t∈[0,1], we define the number of false discoveries V(t), the number of true discoveries S(t), and the total number of discoveries R(t) as the following empirical processes:

$$V(t)≜\#\left\{\;\text{true}\;\text{null}\;P_{j}\;:P_{j}\leq t\right\},\;S(t)≜\#\left\{\;\text{false}\;\text{null}\;P_{j}\;:P_{j}\leq t\right\},\;R(t)≜\#\left\{P_{j}\;:P_{j}\leq t\right\}.$$

Additionally, we define FDP(t)≜V(t)/R(t), where the convention 0/0=0 is used.

Let Ψ≜corr(Σ[A,B,p])>0 denote the population correlation matrix. By Lemma 3, Ψ follows a uniform-block structure, expressed as $\Psi =\Psi \left[A_{\Psi },B_{\Psi },p\right]$, where $A_{\Psi }=C^{-1/2}{AC}^{-1/2}$ and $B_{\Psi }=C^{-1/2}{BC}^{-1/2}$, as provided in (2). Let $\lambda_{j}^{\left(\Psi \right)}>0$ and $\xi_{j}^{\left(\Psi \right)}\in R^{p}$ be the *j*th eigenvalue and its corresponding normalized eigenvector of $\Psi \left[A_{\Psi },B_{\Psi },p\right]$ in decreasing order. Using Lemma 2, these can be computed and then reordered.

For a fixed constant $\kappa_{0}\in \{1,\;\ldots \;,p-1\}$, $\Psi \left[A_{\Psi },B_{\Psi },p\right]$ can be decomposed as

$$\Psi \left[A_{\Psi },B_{\Psi },p\right]=\sum_{j=1}^{\kappa_{0}}\lambda_{j}^{\left(\Psi \right)}\xi_{j}^{\left(\Psi \right)}\xi_{j}^{(\Psi ),⊤}+\sum_{j^{\prime }=\kappa_{0}+1}^{p}\lambda_{j^{\prime }}^{\left(\Psi \right)}\xi_{j^{\prime }}^{\left(\Psi \right)}\xi_{j^{\prime }}^{(\Psi ),⊤}≜L_{p\times \kappa_{0}}L^{⊤}+Q_{p\times p},$$

where $L_{p\times \kappa_{0}}≜\left(l_{1},\;\ldots \;,l_{\kappa_{0}}\right)$ contains the first $\kappa_{0}$ principal components, with $l_{j}≜\sqrt{\lambda_{j}^{\left(\Psi \right)}}\xi_{j}^{\left(\Psi \right)}$ as its *j*th column for $j\in \left\{1,\;\ldots \;,\kappa_{0}\right\}$. Define $Z≜{\left(Z_{1},\;\ldots \;,Z_{p}\right)}^{⊤}≜\sqrt{n}D^{-1}\bar{X}$ and $\overset{˘}{\mu }≜{\left({\overset{˘}{\mu }}_{1},\;\ldots \;,{\overset{˘}{\mu }}_{p}\right)}^{⊤}≜\sqrt{n}D^{-1}\mu$. Then, $Z∼N_{p}\left(\overset{˘}{\mu },D^{-1}\Sigma [A,B,p]D^{-1}\right)=N_{p}\left(\overset{˘}{\mu },\Psi \left[A_{\Psi },B_{\Psi },p\right]\right)$. Accordingly, the *j*th component of Z decomposes as

$$Z_{j}={\overset{˘}{\mu }}_{j}+l^{(j),⊤}w+q_{j},\;j\in \{1,\;\ldots \;,p\},$$

where $l^{(j),⊤}$ is the *j*th row of L, $w∼N_{K}\left(0_{K\times 1},I_{K}\right)$ and $q≜{\left(q_{1},\;\ldots \;,q_{p}\right)}^{⊤}∼N_{p}\left(0_{p\times 1},Q\right)$ are independent random vectors. The oracle FDP and its approximation are defined as:

$${FDP}^{\left(O\right)}(t)≜\sum_{j\in \{\text{true}\;\text{nulls}\}}\frac{\Phi \;\left(\ell_{j}\;\left(z_{t/2}\;+\;\tau_{j}\right)\right)\;+\;\Phi \;\left(\ell_{j}\;\left(z_{t/2}\;-\;\tau_{j}\right)\right)}{R(t)},\;{FDP}^{\left(A\right)}(t)≜\sum_{j=1}^{p}\frac{\Phi \;\left(\ell_{j}\;\left(z_{t/2}\;+\;\tau_{j}\right)\right)\;+\;\Phi \;\left(\ell_{j}\;\left(z_{t/2}\;-\;\tau_{j}\right)\right)}{R(t)},$$

where 0/0 = 0 is used conventionally. Here, $\ell_{j}≜{\left(1-\sum_{j=1}^{\kappa_{0}}l_{j^{\prime },j}^{2}\right)}^{-1/2}$, $l_{j^{\prime },j}$ is the *j*th component of $l^{\left(j^{\prime }\right),⊤}$, $\tau_{j}≜l^{(j),⊤}w$ for j∈{1,…,p}, and $z_{t/2}≜\Phi^{-1}(t/2)$ is the lower t/2 quantile of a standard normal distribution.

Given the estimator $\hat{\Psi }\left[{\hat{A}}_{\Psi },{\hat{B}}_{\Psi },p\right]$ of the population correlation matrix, where

(8) $${\hat{A}}_{\Psi }={\hat{C}}^{-1/2}\hat{A}{\hat{C}}^{-1/2},\;{\hat{B}}_{\Psi }={\hat{C}}^{-1/2}\hat{B}{\hat{C}}^{-1/2}$$

provided in (2). Let ${\hat{L}}_{p\times \kappa_{0}}$ denote the first $\kappa_{0}$ principal components of $\hat{\Psi }\left[{\hat{A}}_{\Psi },{\hat{B}}_{\Psi },p\right]$, and $\hat{w}≜{\left({\hat{L}}^{⊤}\hat{L}\right)}^{-1}{\hat{L}}^{⊤}T$, where the marginal test statistic vector T is defined in (5). We define the adjusted p-values as

(9) $${\hat{P}}_{j}≜2\Phi \left(-\left|{\hat{\ell }}_{j}\left(T_{j}-{\hat{\tau }}_{j}\right)\right|\right),\;j\in \{1,\;\ldots \;,p\},$$

and the FDP estimator as

(10) $$\hat{FDP}(t)≜\sum_{j=1}^{p}\frac{\Phi \;\left({\hat{\ell }}_{j}\;\left(z_{t/2}\;+\;{\overset{ˆ}{\tau }}_{j}\right)\right)\;+\;\Phi \;\left({\hat{\ell }}_{j}\;\left(z_{t/2}\;-\;{\overset{ˆ}{\tau }}_{j}\right)\right)}{R(t)},$$

where the convention 0/0 = 0 is used, ${\hat{\ell }}_{j}≜{\left(1-\sum_{j=1}^{\kappa_{0}}{\overset{ˆ}{l}}_{j^{\prime },j}^{2}\right)}^{-1/2}$, ${\overset{ˆ}{l}}_{j^{\prime },j}$ is the *j*th component of ${\hat{l}}^{\left(j^{\prime }\right),T}$, and ${\hat{l}}^{(j),T}$ is the *j*th row vector of $\hat{L}$, ${\hat{\tau }}_{j}≜{\hat{l}}^{(j),T}\hat{w}$ for j∈{1,…,p}.

#### Remark 8 (*Steps for Estimating FDP Under Uniform-Block Covariance Structures*).

Fix a level of α∈(0,1) and select $\kappa_{0}\in \{1,\;\ldots \;,p-1\}$, where $\kappa_{0}$ can be chosen based on suggestions in Fan and Han (2017).

Step 1: Compute the estimates $\hat{A}$, $\hat{B}$, $\hat{C}$, and $\hat{D}$ using (3).

Step 2: Calculate T using (5) and the corresponding unadjusted p-values $P_{j}$ for j∈{1,…,p} using (7), where the degrees of freedom parameters are given in (6).

Step 3: Set candidate threshold values: $t_{1},\;\ldots \;,t_{S}\in [0,\;1]$, where S is pre-determined, say S=200.

Step 4: Compute the numbers of total discoveries $R\left(t_{s}\right)≜\#\left\{P_{j}\;:P_{j}\leq t_{s}\right\}$ using the unadjusted p-values $P_{j}$ from Step 2 for each s∈{1,…,S}.

Step 5: Compute the estimates $\hat{\Psi }\left[{\hat{A}}_{\Psi },{\hat{B}}_{\Psi },p\right]$, ${\hat{A}}_{\Psi }$, ${\hat{B}}_{\Psi }$ using (8) and results from Step 1; then, calculate its eigenvalues and eigenvectors using Lemma 2; thus, construct $\hat{L}$ from the first $\kappa_{0}$ principal components.

Step 6: Calculate $\hat{w}≜{\left({\hat{L}}^{⊤}\hat{L}\right)}^{-1}{\hat{L}}^{⊤}T$, alternatively, $\hat{w}$ can be obtained using the quantile regression or other robust regression approaches (Vutov and Dickhaus, 2023a,b); then compute ${\hat{\tau }}_{j}≜{\hat{l}}^{(j),⊤}\hat{w}$ for j∈{1,…,p}.

Step 7: Calculate the adjusted p-values ${\hat{P}}_{j}$ using (9) for j∈{1,…,p} and the FDP(t) estimator $\hat{FDP}(t)$ using (10).

Step 8: Determine $t_{s_{\alpha }}≜max\left\{t_{s}\;:\hat{FDP}\left(t_{s}\right)\leq \alpha ,s\in \{1,\;\ldots \;,S\}\right\}$ : the largest threshold $t_{s}$ satisfying $\hat{FDP}\left(t_{s}\right)\leq \alpha$.

Step 9: Reject the null hypotheses $H_{5_{j}}$ if the adjusted p-values ${\hat{P}}_{j}\leq t_{s_{\alpha }}$ for j∈{1,…,p}.

The following theorem, adapted from Fan and Han (2017), establishes the approximation bounds for the proposed FDP estimator.

#### Theorem 4 (*Approximation Bounds for the FDP Estimator*).

For the population correlation matrix $\Psi \left[A_{\Psi },B_{\Psi },p\right]$, suppose there exists $\delta_{1}>0$, $\delta_{2}\geq 0$, and $\delta_{3}<\infty$ satisfying that $\sqrt{\lambda_{\kappa_{0}+1}^{(\Psi ),2}+\cdots +\lambda_{p}^{(\Psi ),2}}/p=O\left(p^{-\delta_{1}}\right)$, $R^{-1}(t)=O\left(p^{-\left(1-\delta_{2}\right)}\right)$, and $\ell_{j}\leq \delta_{3}$ for j∈{1,…,p}, respectively. Additionally, suppose $\lambda_{j}^{\left(\Psi \right)}-\lambda_{j+1}^{\left(\Psi \right)}\geq g_{p}$ for a positive sequence $g_{p}$ for $j\in \left\{1,\;\ldots \;,\kappa_{0}\right\}$. Then, given the proposed estimator in (8), for sufficiently large p and some $\delta_{4}>0$,

$$\left|{FDP}^{\left(O\right)}(t)-FDP(t)\right|=O_{P}\left\{p^{\delta_{2}}\left(p^{-\delta_{1}/2}+n^{-1/2}\right)\right\},$$

$$\left|\hat{FDP}(t)-{FDP}^{\left(A\right)}(t)\right|=O_{P}\left[p^{\delta_{2}}\left\{\kappa_{0}p^{-\delta_{4}}g_{p}/p+\left(\kappa_{0}+1\right)p^{-\delta_{4}}+\|\overset{˘}{\mu }\|p^{-1/2}\right\}\right].$$

#### Remark 9 (*High-Dimensionality in Scenario 3*).

Note that the estimators (3) or (8) require that n>K+(K+1)K/2. However, when K>n and $K≜K_{n}$ grows with n, Yang et al. (2024) proposed a modified hard-thresholding estimators for A and B (or $A_{\Psi }$ and $B_{\Psi }$) that achieve consistency in both Frobenius and spectral norms. Consequently, if $K_{n}>n$ and $log\left(K_{n}\right)/n\to 0$ as n→∞. Theorem 4 remains valid if we replace the estimators (3) or (8) with the modified hard-thresholding estimators.

## 4. Discussion

In this paper, we focus on high-dimensional hypothesis testing under uniform-block covariance structures, motivated by their three features. First, the uniform-block pattern has been popularly discovered in plenty of high-dimensional data across various fields, including biomedicine, engineering, and finance. Second, it offers clear interpretability: variables grouped within the same community (or block) tend to exhibit stochastic equivalence or comparable patterns, while those from different communities maintain coherent connections at the community level. Third, this structure enables efficient computation in high-dimensional (with missing data) settings by leveraging its block pattern. Therefore, compared with conventional structures such as diagonal or block-diagonal forms, the proposed uniform-block structure provides greater flexibility, making it particularly well-suited for large-scale or high-dimensional multivariate data analysis. Additionally, by incorporating information from the non-zero off-diagonal blocks, this structure has the potential to reveal valuable insights into underlying scientific mechanisms.

To establish the algebraic properties of uniform-block matrices, we introduced an innovative block Hadamard product representation. This representation allows the decomposition of a large-scale uniform-block matrix into two lower-dimensional matrices and an integer-valued vector, thereby simplifying matrix computations. These properties enhance the applicability of uniform-block structures to various statistical problems, particularly in hypothesis testing.

We addressed hypothesis testing problems related to covariance structures, mean vectors, and marginal mean components, respectively, under uniform-block structures. In contrast to traditional methods, the proposed test statistics are applicable in high-dimensional scenarios, with their null distributions derived in closed form. Furthermore, these test statistics demonstrated robustness against minor or moderate structural perturbations, missing data, and distributional misspecification.

In conclusion, hypothesis testing under uniform-block covariance structures, along with its associated algebraic properties, has broad applicability across various disciplines, encompassing fields such as linear algebra, statistics, economics, and numerous others.

## Supplementary Material

Supplementary Material

## Appendix A. Preliminaries

### Definition A.1 (*Wishart Distribution*, (Kollo and von Rosen, 2005, Definition 2.4.1), (Fujikoshi et al., 2010, Definition 2.1.1), (Muirhead, 2005, Definition 10.3.1)).

Let $X_{1},\;\ldots \;,X_{n}\overset{\text{i.i.d.}}{∼}N_{p}(\mu ,\Sigma )$ be p-dimensional normal vectors. The p by p matrix $W≜\sum_{i=1}^{n}X_{i}X_{i}^{⊤}$ is defined to follow a Wishart distribution, denoted as $W∼W_{p}(\Sigma ,n;\Omega )$. Here, Ω represents the first or second type of noncentrality parameter, defined as either $\Omega^{\left(I\right)}≜\mu \mu^{⊤}\in R^{p\times p}$ or $\Omega^{\left(II\right)}≜\Sigma^{-1}(n\Omega^{\left(I\right)})=n\Sigma^{-1}\Omega^{\left(I\right)}\in R^{p\times p}$.

### Lemma A.1 (*Closure of Wishart Distributions Under Transformations*, (Kollo and von Rosen, 2005, Theorem 2.4.2), (Fujikoshi et al., 2010, Theorem 2.2.1 (2)), (Muirhead, 2005, Theorem 10.3.5)).

Let $A\in R^{q\times p}$ be a matrix of rank(A)=q. If $W∼W_{p}\left(\Sigma ,n;\Omega^{\left(I\right)}\right)$, then the transformed matrix follows $AWA^{⊤}∼W_{q}\left(A\Sigma A^{⊤},n;A\Omega^{\left(I\right)}A^{⊤}\right)$. Similarly, if $W∼W_{p}\left(\Sigma ,n;\Omega^{\left(II\right)}\right)$, then the transformed matrix follows $AWA^{⊤}∼W_{q}\left(A\Sigma A^{⊤},n;{\left(A\Sigma A^{⊤}\right)}^{-1}A\left(\Sigma \Omega^{\left(II\right)}\right)A^{⊤}\right)$.

### Lemma A.2 (*Decomposition and Distribution of a Weighted Quadratic Form*).

Suppose the p-dimensional random vectors $Y_{1},\;\ldots \;,Y_{n}$ are mutually independent, with $Y_{i}∼N_{p}\left(\omega_{i}\mu_{i},\Sigma \right)$ for i∈{1,…,n}. Let $\bar{Y}$ denote the weighted mean defined by $\bar{Y}≜\left(\sum_{i=1}^{n}\omega_{i}Y_{i}\right)/\left(\sum_{i=1}^{n}\omega_{i}^{2}\right)$.

- Suppose $\mu_{i}≜\mu$ for all i∈{1,…,n}. Then, the decomposition
  $$\sum_{i=1}^{n}\left(Y_{i}-\omega_{i}\mu \right){\left(Y_{i}-\omega_{i}\mu \right)}^{⊤}=\sum_{i=1}^{n}\left(Y_{i}-\omega_{i}\bar{Y}\right){\left(Y_{i}-\omega_{i}\bar{Y}\right)}^{⊤}+\sum_{i=1}^{n}\omega_{i}^{2}(\bar{Y}-\mu )(\bar{Y}-\mu {)}^{⊤}$$
  holds.
- The quadratic form $\sum_{i=1}^{n}\left(Y_{i}-\omega_{i}\bar{Y}\right){\left(Y_{i}-\omega_{i}\bar{Y}\right)}^{⊤}$ follows $W_{p}\left(\Sigma ,rank\left(A_{Y}\right);\Omega^{\left(I\right)}\right)$, where $\Omega^{\left(I\right)}≜M_{Y}A_{Y}M_{Y}^{⊤}$, $A_{Y}≜I_{n}-{\left(\omega_{1},\;\ldots \;,\omega_{n}\right)}^{⊤}\left(\omega_{1},\;\ldots \;,\omega_{n}\right)/\left(\sum_{i=1}^{n}\omega_{i}^{2}\right)\in R^{n\times n}$, and $M_{Y}≜\left(E\left(Y_{1}\right),\;\ldots \;,E\left(Y_{n}\right)\right)=\left(\omega_{1}\mu_{1},\;\ldots \;,\omega_{n}\mu_{n}\right)\in R^{p\times n}$.

### Proof of Lemma A.2.

For the first part, we expand the sum of the centered outer products via the additive split $\left(Y_{i}-\omega_{i}\mu )=(Y_{i}-\omega_{i}\bar{Y})+(\omega_{i}\bar{Y}-\omega_{i}\mu \right)$. The cross-term vanishes identically. For the second part, let $\alpha ≜\sum_{i=1}^{n}\omega_{i}^{2}$ and define the vector $\beta ≜{\left(\beta_{1},\;\ldots \;,\beta_{n}\right)}^{⊤}$ where $\beta_{i}≜\omega_{i}/\alpha$ for every i. Let $e_{i}$ denote the standard basis vector with 1 at the *i*th coordinate and 0 otherwise. Consequently, $\sum_{i=1}^{n}(Y_{i}-\omega_{i}\bar{Y}){\left(Y_{i}-\omega_{i}\bar{Y}\right)}^{⊤}≜{YA}_{Y}Y^{⊤}$, where $A_{Y}≜\sum_{i=1}^{n}\left(e_{i}-\omega_{i}\beta \right){\left(e_{i}-\omega_{i}\beta \right)}^{⊤}\in R^{n\times n}$. Note that $\sum_{i=1}^{n}\omega_{i}e_{i}=\alpha \beta$. We simplify $A_{Y}$ as $A_{Y}=I_{n}-\alpha \beta \beta^{⊤}$, which is idempotent. Using the result (Singull and Koski, 2012, Corollary 2.1), we conclude that $YA_{Y}Y^{⊤}∼W_{p}\left(\Sigma ,rank\left(A_{Y}\right),\Omega^{\left(I\right)}\right)$, where $\Omega^{\left(I\right)}=E(Y)A_{Y}E{\left(Y\right)}^{⊤}$.

### Lemma A.3 (*Craig’s Theorem*, (Ravishanker and Dey, 2002, Result 5.4.4), (Mathai and Provost, 1992, Theorem 5.2.1)).

Suppose $X∼N_{p}(\mu ,\Sigma )$ with Σ>0. Let $A_{1},A_{2}\in R^{p\times p}$ be two symmetric matrices. Then, $X^{⊤}A_{1}X$ and $X^{⊤}A_{2}X$ are independent if and only if $A_{1}\Sigma A_{2}=0_{p\times p}$.

### Lemma A.4 (*Distribution of Quadratic Forms of Multivariate Normal Vectors*, (Muirhead, 2005, Theorem 1.4.5), (Mathai and Provost, 1992, Theorem 5.1.3), (Zhang, 2018)).

Suppose $X∼N_{p}(\mu ,\Sigma )$ with Σ>0. Let $A\in R^{p\times p}$ be a symmetric matrix. Then, $X^{⊤}AX∼\chi^{2}(rank(A\Sigma );\delta )$ with noncentrality parameter $\delta =\mu^{⊤}A\mu$, if and only if AΣ is idempotent.

### Lemma A.5 (*One-Sample LRTs*).

Suppose $Y_{1},\;\ldots \;,Y_{n}\overset{\text{i.i.d}}{∼}N_{p}(\nu ,n\Xi )$ with Ξ>0. Define the sample mean $\bar{Y}≜\left(Y_{1}+\cdots +Y_{n}\right)/n$ and the matrix V such that $nV≜\sum_{i=1}^{n}\left(Y_{i}-\bar{Y}\right){\left(Y_{i}-\bar{Y}\right)}^{⊤}$. Consider the following parameter spaces:

$$\omega_{1}\;:\Xi \;\mathrm{is}\;\mathrm{unstructured;}\;\omega_{2}:\Xi =Bdiag\left(\tilde{\Delta },a_{11}I_{p_{1}-1},\;\ldots \;,a_{KK}I_{p_{K}-1}\right);\omega_{3}\;:v=v_{0},\Xi =Bdiag\left(\tilde{\Delta },a_{11}I_{p_{1}-1},\;\ldots \;,a_{KK}I_{p_{K}-1}\right).$$

For $\omega_{3}$, $v_{0}\in R^{p\times 1}$ is known. If n>p, then the likelihood ratio between $\omega_{2}$ and $\omega_{1}$ is

$$\frac{{sup}_{\omega_{2}}\;L\;\left(v,\;\Xi ;\;Y_{1},\;\ldots \;,Y_{n}\right)}{{sup}_{\omega_{1}}\;L\;\left(v,\;\Xi ;\;Y_{1},\;\ldots \;,Y_{n}\right)}={\left[\left\{\prod_{k=1}^{K}{\left(p_{k}-1\right)}^{p_{k}-1}\right\}\frac{det\;(V)}{det\;\left(V_{00}\right)}\frac{1}{\prod_{k=1}^{K}tr\;{\left(V_{kk}\right)}^{p_{k}-1}}\right]}^{n/2}.$$

If $\hat{A}>0$ and $\hat{\tilde{\Delta }}=\hat{A}+P^{1/2}\hat{B}P^{1/2}>0$, then the likelihood ratio between $\omega_{3}$ and $\omega_{2}$ is

$$\frac{{sup}_{\omega_{3}}\;L\;\left(v,\;\Xi ;\;Y_{1},\;\ldots \;,Y_{n}\right)}{{sup}_{\omega_{2}}\;L\;\left(v,\;\Xi ;\;Y_{1},\;\ldots \;,Y_{n}\right)}={\left[\frac{det\;\left(V_{00}\right)}{det\;\left(V_{00}\;+\;H_{00}\right)}\prod_{k=1}^{K}{\left\{\frac{tr\;\left(V_{kk}\right)}{tr\;\left(V_{kk}\;+\;H_{kk}\right)}\right\}}^{p_{k}-1}\right]}^{n/2},$$

where $H≜\left(\bar{Y}-v_{0}\right){\left(\bar{Y}-v_{0}\right)}^{⊤}\in R^{p\times p}$, $V=\left(V_{kk^{\prime }}\right)\;and\;H=\left(H_{kk^{\prime }}\right)$ are partitioned by a (K+1)-dimensional partition-size vector ${\left(K,p_{1}-1,\;\ldots \;,p_{K}-1\right)}^{⊤}$ for $k,k^{\prime }\in \{0,\;1,\;\ldots \;,K\}$.

### Proof of Lemma A.5.

Let c≜c(n,p) denote the constant −nplog(2π)/2−(nplogn)/2. Under $\omega_{1}$, $\omega_{2}$, and $\omega_{3}$, we derive the supremum of the log-likelihood, respectively:

$$\underset{\omega_{1}}{sup}\;log\;L\;\left(v,\Xi ;Y_{1},\;\ldots \;,Y_{n}\right)=c-(n/2)\{p+log\;det\;(V/n)\},$$

$$\underset{\omega_{2}}{sup}\;log\;L\;\left(v,\Xi ;Y_{1},\;\ldots \;,Y_{n}\right)=c-\frac{n}{2}\left\{p+log\;det\;\left(V_{00}/n\right)\right\}-\frac{n}{2}\sum_{k=1}^{K}\left(p_{k}-1\right)log\left\{\frac{tr\left(V_{kk}/n\right)}{p_{k}\;-\;1}\right\},$$

$$\underset{\omega_{3}}{sup}\;log\;L\;\left(v,\Xi ;Y_{1},\;\ldots \;,Y_{n}\right)=c-\frac{n}{2}\left\{p+log\;det\;\left(D_{00}/n\right)\right\}-\frac{n}{2}\sum_{k=1}^{K}\left(p_{k}-1\right)log\left\{\frac{tr\left(D_{kk}/n\right)}{p_{k}\;-\;1}\right\},$$

where $D≜V+\left(\bar{Y}-v_{0}\right){\left(\bar{Y}-v_{0}\right)}^{⊤}\in R^{p\times p}$, $V=\left(V_{kk^{\prime }}\right)$ and $D=\left(D_{kk^{\prime }}\right)$ are partitioned by a (K+1)-dimensional partition-size vector ${\left(K,p_{1}-1,p_{2}-1,\;\ldots \;,p_{K}-1\right)}^{⊤}$ for $k,k^{\prime }\in \{0,\;1,\;\ldots \;,K\}$. We emphasize that the assumption n>p ensures $\hat{\Xi }\left(\omega_{1}\right)=V/n>0$ with probability 1, making the likelihood value finite.

Lastly, we straightforwardly compute the likelihood ratios between $\omega_{2}$ and $\omega_{1}$, and between $\omega_{3}$ and $\omega_{2}$.

### Lemma A.6 (*Multiple-Sample LRTs*).

Suppose $Y_{1}^{\left(m\right)},\;\ldots \;,Y_{n^{\left(m\right)}}^{\left(m\right)}\overset{\mathrm{i.i.d.}}{∼}N_{p}\left(\nu^{\left(m\right)},n^{\left(m\right)}\Xi^{\left(m\right)}\right)$ with $\Xi^{\left(m\right)}>0$ for m∈{1,…,M}. Define the sample mean ${\bar{Y}}^{\left(m\right)}≜\left(Y_{1}^{\left(m\right)}+\cdots +Y_{n^{\left(m\right)}}^{\left(m\right)}\right)/n^{\left(m\right)}$, the pooled mean $\bar{Y}≜\left(n^{\left(1\right)}{\bar{Y}}^{\left(1\right)}+\cdots +n^{\left(m\right)}{\bar{Y}}^{\left(m\right)}\right)/n$, the matrix $V^{\left(m\right)}$ such that $n^{\left(m\right)}V^{\left(m\right)}≜\sum_{i=1}^{n^{\left(m\right)}}\left(Y_{i}^{\left(m\right)}-{\bar{Y}}^{\left(m\right)}\right)\left(Y_{i}^{\left(m\right)}-{\bar{Y}}^{\left(m\right)}\right)$, and the weight $f^{\left(m\right)}≜n^{\left(m\right)}/n$ for m∈{1,…,M}. Consider the following parameter spaces:

$$\omega_{1}:{\tilde{v}}^{\left(m\right)}\;\mathrm{is}\;\mathrm{unstructured}\;\mathrm{and}\;\Xi^{\left(m\right)}=Bdiag\left({\tilde{\Delta }}^{\left(m\right)},a_{11}^{\left(m\right)}I_{p_{1}-1},\;\ldots \;,a_{KK}^{\left(m\right)}I_{p_{K}-1}\right),m\in \{1,\;\ldots \;,M\},$$

$$\omega_{2}:{\tilde{v}}^{\left(m\right)}\;\mathrm{is}\;\mathrm{unstructured}\text{,}\;m\in \{1,\;\ldots \;,M\},\;\mathrm{and}\;\Xi^{\left(1\right)}=\cdots \Xi^{\left(m\right)}=Bdiag\left(\tilde{\Delta },a_{11}I_{p_{1}-1},\;\ldots \;,a_{KK}I_{p_{K}-1}\right),$$

$$\omega_{3}:{\tilde{v}}^{\left(1\right)}=\cdots ={\tilde{v}}^{\left(m\right)}\;\text{and}\;\Xi^{\left(1\right)}=\cdots \Xi^{\left(m\right)}=Bdiag\left(\tilde{\Delta },a_{11}I_{p_{1}-1},\;\ldots \;,a_{KK}I_{p_{K}-1}\right),$$

where ${\tilde{v}}^{\left(m\right)}≜v^{\left(m\right)}/\sqrt{n^{\left(m\right)}}$ for m∈{1,…,M}. If ${\hat{A}}^{\left(m\right)}>0$, ${\hat{\tilde{\Delta }}}^{\left(m\right)}≜{\hat{A}}^{\left(m\right)}+P^{1/2}{\hat{B}}^{\left(m\right)}P^{1/2}>0$ for m∈{1,…,M}, $\hat{A}>0$, and $\hat{\tilde{\Delta }}≜\hat{A}+P^{1/2}\hat{B}P^{1/2}>0$ with probability 1, then the likelihood ratio between $\omega_{2}$ and $\omega_{1}$ is

$$\frac{{sup}_{\omega_{2}}\;L\;\left(v^{\left(m\right)},\;\Xi^{\left(m\right)};\;Y_{1}^{\left(m\right)},\;\ldots \;,Y_{n^{\left(m\right)}}^{\left(m\right)},\;\forall m\right)}{{sup}_{\omega_{1}}\;L\;\left(v^{\left(m\right)},\;\Xi^{\left(m\right)};\;Y_{1}^{\left(m\right)},\;\ldots \;,Y_{n^{\left(m\right)}}^{\left(m\right)},\;\forall m\right)}={\left(\prod_{m=1}^{M}f^{(m),f^{\left(m\right)}}\right)}^{-np/2}\prod_{m=1}^{M}{\left[\frac{det\;\left(V_{00}^{\left(m\right)}\right)}{det\;\left(\sum_{m=1}^{M}V_{00}^{\left(m\right)}\right)}\prod_{k=1}^{K}{\left\{\frac{tr\;\left(V_{kk}^{\left(m\right)}\right)}{tr\;\left(\sum_{m=1}^{M}V_{kk}^{\left(m\right)}\right)}\right\}}^{p_{k}-1}\right]}^{n^{\left(m\right)}/2}.$$

If $\hat{A}>0$ and $\hat{\tilde{\Delta }}≜\hat{A}+P^{1/2}\hat{B}P^{1/2}>0$ with probability 1, then the likelihood ratio between $\omega_{3}$ and $\omega_{2}$ is

$$\frac{{sup}_{\omega_{3}}\;L\;\left(v^{\left(m\right)},\;\Xi^{\left(m\right)};\;Y_{1}^{\left(m\right)},\;\ldots \;,Y_{n^{\left(m\right)}}^{\left(m\right)},\;\forall m\right)}{{sup}_{\omega_{2}}\;L\;\left(v^{\left(m\right)},\;\Xi^{\left(m\right)};\;Y_{1}^{\left(m\right)},\;\ldots \;,Y_{n^{\left(m\right)}}^{\left(m\right)},\;\forall m\right)}={\left[\frac{det\;\left(\sum_{m=1}^{M}V_{00}^{\left(m\right)}\right)}{det\;\left(H_{00}\;+\;\sum_{m=1}^{M}V_{00}^{\left(m\right)}\right)}\prod_{k=1}^{K}{\left\{\frac{tr\;\left(\sum_{m=1}^{M}V_{kk}^{\left(m\right)}\right)}{tr\;\left(H_{kk}\;+\;\sum_{m=1}^{M}V_{kk}^{\left(m\right)}\right)}\right\}}^{p_{k}-1}\right]}^{n/2},$$

where $H≜\sum_{m=1}^{M}\left({\bar{Y}}^{\left(m\right)}-\sqrt{n^{\left(m\right)}}\bar{Y}\right){\left({\bar{Y}}^{\left(m\right)}-\sqrt{n^{\left(m\right)}}\bar{Y}\right)}^{⊤}\in R^{p\times p}$, $V=\left(V_{kk^{\prime }}\right)\;and\;H=\left(H_{kk^{\prime }}\right)$ are partitioned by a (K+1)-dimensional partition-size vector ${\left(K,p_{1}-1,\;\ldots \;,p_{K}-1\right)}^{⊤}$ for $k,k^{\prime }\in \{0,\;1,\;\ldots \;,K\}$.

### Proof of Lemma A.6.

Let $c_{M}≜c\left(n^{\left(1\right)},\;\ldots \;,n^{\left(m\right)},p,M\right)$ denote the constant $-np\;log(2\pi )/2-\sum_{m=1}^{M}n^{\left(m\right)}p\;log\left(n^{\left(m\right)}\right)/2$. Under $\omega_{1}$, $\omega_{2}$, and $\omega_{3}$, we derive the supremum of the log-likelihood, respectively:

$$\underset{\omega_{1}}{sup}\log L\left(v^{\left(m\right)},\Xi^{\left(m\right)};Y_{1}^{\left(m\right)},\;\ldots \;,Y_{n^{\left(m\right)}}^{\left(m\right)},\forall m\right)=c_{M}-\sum_{m=1}^{M}\frac{n^{\left(m\right)}}{2}\left\{p+\log det\;\left(\frac{V_{00}^{\left(m\right)}}{n^{\left(m\right)}}\right)\right\}-\sum_{m=1}^{M}\frac{n^{\left(m\right)}}{2}\sum_{k=1}^{K}\left(p_{k}-1\right)\;\log\left\{\frac{tr\;\left(\frac{V_{kk}^{\left(m\right)}}{n^{\left(m\right)}}\right)}{p_{k}\;-\;1}\right\},$$

$$\underset{\omega_{2}}{sup}\;log\;L\;\left(v^{\left(m\right)},\Xi^{\left(m\right)};Y_{1}^{\left(m\right)},\;\ldots \;,Y_{n^{\left(m\right)}}^{\left(m\right)},\forall m\right)=c_{M}-\frac{n}{2}\left\{p+log\;det\;\left(\sum_{m=1}^{M}\frac{V_{00}^{\left(m\right)}}{n}\right)\right\}-\frac{n}{2}\sum_{k=1}^{K}\left(p_{k}-1\right)\;log\left\{\frac{tr\;\left(\sum_{m=1}^{M}V_{kk}^{\left(m\right)}/n\right)}{p_{k}\;-\;1}\right\},$$

$$\underset{\omega_{3}}{sup}\;log\;L\;\left(v^{\left(m\right)},\Xi^{\left(m\right)};Y_{1}^{\left(m\right)},\;\ldots \;,Y_{n^{\left(m\right)}}^{\left(m\right)},\forall m\right)=c_{M}-\frac{n}{2}\left\{p+log\;det\;\left(\frac{D_{00}}{n}\right)\right\}-\frac{n}{2}\sum_{k=1}^{K}\left(p_{k}-1\right)\;log\left\{\frac{tr\;\left(D_{kk}/n\right)}{p_{k}\;-\;1}\right\},$$

where $H≜\sum_{m=1}^{M}\left({\bar{Y}}^{\left(m\right)}-\sqrt{n^{\left(m\right)}}\bar{Y}\right){\left({\bar{Y}}^{\left(m\right)}-\sqrt{n^{\left(m\right)}}\bar{Y}\right)}^{⊤}\in R^{p\times p}$, $D≜\sum_{m=1}^{M}V^{\left(m\right)}+H\in R^{p\times p}$, $V^{\left(m\right)}=\left(V_{kk^{\prime }}^{\left(m\right)}\right)$ and $D=\left(D_{kk^{\prime }}\right)$ are partitioned by a (K+1)-dimensional partition-size vector ${\left(K,p_{1}-1,p_{2}-1,\;\ldots \;,p_{K}-1\right)}^{⊤}$ for $k,k^{\prime }\in \{0,\;1,\;\ldots \;,K\}$ and m∈{1,…,M}.

Lastly, we straightforwardly compute the likelihood ratios between $\omega_{2}$ and $\omega_{1}$, and between $\omega_{3}$ and $\omega_{2}$.

## Appendix B. Proofs of main results

### Proof of Lemma 1.

The representation holds by direct calculation. To establish uniqueness, consider two equal uniform-block matrices $N_{1}\left[A_{1},B_{1},p\right]$ and $N_{2}\left[A_{2},B_{2},p\right]$. Compute the difference. By the definition of a zero matrix, we have $A_{1}=A_{2}$ and $B_{1}=B_{2}$ if $p_{k}>1$.

### Proof of Lemma 2.

Property (i) follows by direct calculation. Properties (ii), (iii), and (vi) follow from the identity $\left(B_{1}\circ J[p]\right)$ $\left(A_{2}\circ I[p]\right)=\left(B_{1}A_{2}\right)\circ J[p]$. Properties (iv) and (v) are established via induction on K.

Property (vii) follows from a blockwise calculation of $\Gamma N[A,B,p]\Gamma^{⊤}$. This derivation utilizes the identities $1_{n\times p}1_{p\times q}=p1_{n\times q}$, as well as the orthogonality properties ${\tilde{H}}_{(p-1)\times p}1_{p\times q}=0_{(p-1)\times q},1_{p\times q}{\tilde{H}}_{q\times (q-1)}^{⊤}=0_{p\times (q-1)}$.

### Proof of Lemma 3.

The results follow directly from Lemmas 1 and 2.

### Proof of Theorem 1.

For simplicity, we suppress the superscript (1). We define $Y≜\sqrt{n}\Gamma \bar{X}$, $v≜\sqrt{n}\Gamma \mu$, $V≜\Gamma W\Gamma^{⊤}$, and let $\Gamma \Sigma \Gamma^{⊤}$ denote a general form of Σ. Using the first part of Lemma A.5, the likelihood ratio statistic $\Lambda_{1}$ is computed as

$$\Lambda_{1}^{2/n}=\left\{\prod_{k=1}^{K}{\left(p_{k}-1\right)}^{p_{k}-1}\right\}\left\{\frac{det\;(V)}{det\;\left(V_{00}\right)}\right\}{\left\{\prod_{k=1}^{K}tr\;{\left(V_{kk}\right)}^{p_{k}-1}\right\}}^{-1},$$

where $V=\left(V_{kk^{\prime }}\right)$ is partitioned by a (K+1)-dimensional partition-size vector ${\left(K,p_{1}-1,\;\ldots \;,p_{K}-1\right)}^{⊤}$ for $k,k^{\prime }\in \{0,\;1,\;\ldots \;,K\}$. Furthermore, we define that

$$\Lambda_{1}^{\left(0\right)}≜{\left\{det(V)/\prod_{k=0}^{K}det\;\left(V_{kk}\right)\right\}}^{n/2},\;\Lambda_{1}^{\left(k\right)}≜{\left\{\frac{det\;\left(V_{kk}\right)}{tr\;{\left(V_{kk}/\left(p_{k}\;-\;1\right)\right)}^{p_{k}-1}}\right\}}^{n/2},\;k\in \{1,\;\ldots \;,K\},$$

satisfying $\Lambda_{1}=\prod_{k=0}^{K}\Lambda_{1}^{\left(k\right)}$. Specifically, $\Lambda_{1}^{\left(0\right)}$ is the likelihood ratio test statistic for testing the independence of (K+1) sets of variables, and $\Lambda_{1}^{\left(k\right)}$ is the likelihood ratio test statistic for testing the sphericity of the *k*th diagonal block.

For the second part of Theorem 1, we define $Y^{\left(m\right)}≜\sqrt{n^{\left(m\right)}}\Gamma {\bar{X}}^{\left(m\right)}$, $v^{\left(m\right)}≜\sqrt{n^{\left(m\right)}}\Gamma \mu^{\left(m\right)}$, $\Xi^{\left(m\right)}≜\Gamma \Sigma^{\left(m\right)}[A,B,p]\Gamma^{⊤}$, for m∈{1,…,M}. Using the first part of Lemma A.6, the likelihood ratio statistic $\Lambda_{3}$ is computed as

$$\Lambda_{3}^{2/n}={\left(\prod_{m=1}^{M}f^{(m),f^{\left(m\right)}}\right)}^{-np/2}\prod_{m=1}^{M}{\left[det\;\left(V_{00}^{\left(m\right)}\right)/det\;\left(\sum_{m=1}^{M}V_{00}^{\left(m\right)}\right)\prod_{k=1}^{K}{\left\{tr\;\left(V_{kk}^{\left(m\right)}\right)\;/\;tr\;\left(\sum_{m=1}^{M}V_{kk}^{\left(m\right)}\right)\right\}}^{p_{k}-1}\right]}^{n^{\left(m\right)}/2}.$$

First, under $H_{3}$, $V^{\left(m\right)}∼W_{p}\left(\Xi^{\left(m\right)},n^{\left(m\right)}-1\right)=W_{p}\left(\Xi ,n^{\left(m\right)}-1\right)$ for m∈{1,…,M}. Setting the K by p matrix $\left(I_{K}\;0_{K\times (p-K)}\right)$ as A (which has rank) in Lemma A.1, we have that $V_{00}^{\left(m\right)}∼W_{K}\left(\tilde{\Delta },n^{\left(m\right)}-1\right)$ for m∈{1,…,M} and they are mutually independent across m. Thus,

$$det\;\left(V_{00}^{\left(m\right)}\right)/det\;\left(\sum_{m=1}^{M}V_{00}^{\left(m\right)}\right)=det\;\left(\left(V_{00}^{\left(m\right)}\right){\left(\sum_{m=1}^{M}V_{00}^{\left(m\right)}\right)}^{-1}\right)=det\;\left({\left(\sum_{m=1}^{M}V_{00}^{\left(m\right)}\right)}^{-1/2}\left(V_{00}^{\left(m\right)}\right){\left(\sum_{m=1}^{M}V_{00}^{\left(m\right)}\right)}^{-1/2}\right),$$

where $A^{1/2}$ denotes the square root matrix of matrix A. We have that M variates

$$\left({\left(\sum_{m=1}^{M}V_{00}^{\left(m\right)}\right)}^{-1/2}\left(V_{00}^{\left(1\right)}\right){\left(\sum_{m=1}^{M}V_{00}^{\left(m\right)}\right)}^{-1/2},\;\ldots \;,{\left(\sum_{m=1}^{M}V_{00}^{\left(m\right)}\right)}^{-1/2}\left(V_{00}^{\left(m\right)}\right){\left(\sum_{m=1}^{M}V_{00}^{\left(m\right)}\right)}^{-1/2}\right)$$

jointly follow a K-dimensional matrix variate Dirichlet (type I) distribution with parameters $\left(\left(n^{\left(1\right)}-1\right)/2,\;\ldots \;,\left(n^{\left(m\right)}-1\right)/2;0\right)$ (Gupta and Nagar, 1999, Theorem 6.2.1).

Second, under $H_{3}$, $V^{\left(m\right)}∼W_{p}\left(\Xi^{\left(m\right)},n^{\left(m\right)}-1\right)=W_{p}\left(\Xi ,n^{\left(m\right)}-1\right)$ for m∈{1,…,M}. Setting the K by p matrices $\left(\begin{array}{lll} 0_{K\times K} & I_{p_{1}-1} & 0_{K\times \left(p-K-\left(p_{1}-1\right)\right)} \end{array}\right),\;\ldots \;,\left(0_{K\times \left(K+p-\left(p_{K}-1\right)\right)}I_{p_{K}-1}\right)$ as A with ranks of $p_{1}-1,\;\ldots \;,p_{K}-1$ in Lemma A.1, respectively, we have that $V_{kk}^{\left(m\right)}∼W_{p_{k}-1}\left(a_{kk}I_{p_{k}-1},n^{\left(m\right)}-1\right)$ for k∈{1,…,K}. Thus, $tr\;\left(V_{kk}^{\left(m\right)}\right)∼a_{kk}\chi^{2}\left(\left(n^{\left(m\right)}-1\right)\left(p_{k}-1\right)\right)$ for k∈{1,…,K} and m∈{1,…,M} (Kollo and von Rosen, 2005, Corollary 2.4.2.2), and

$$tr\;\left(V_{kk}^{\left(m\right)}\right)\;/\;tr\;\left(\sum_{m=1}^{M}V_{kk}^{\left(m\right)}\right)∼a_{kk}\chi^{2}\left(\left(n^{\left(m\right)}-1\right)\left(p_{k}-1\right)\right)/\left\{\sum_{m=1}^{M}a_{kk}\chi^{2}\left(\left(n^{\left(m\right)}-1\right)\left(p_{k}-1\right)\right)\right\}.$$

Applying Ng et al. (2011, Theorem 2.1), we obtain that

$$\left(tr\;\left(V_{kk}^{\left(1\right)}\right)\;/\;tr\;\left(\sum_{m=1}^{M}V_{kk}^{\left(m\right)}\right),\;\ldots \;,tr\;\left(V_{kk}^{\left(m\right)}\right)\;/\;tr\;\left(\sum_{m=1}^{M}V_{kk}^{\left(m\right)}\right)\right)∼{Dirichlet}_{M}\left(\left(n^{\left(1\right)}-1\right)\left(p_{k}-1\right)/2,\;\ldots \;,\left(n^{\left(m\right)}-1\right)\left(p_{k}-1\right)/2\right).$$

Putting them together, we complete the proof.

### Proof of Corollary 1.

We first obtain the moment $E\left(\Lambda_{1}^{h}\right)=\prod_{j=1}^{p}\Gamma ((n-j+nh)/2)/\Gamma ((n-j)/2)\prod_{k=1}^{K}\Gamma ((n-k)/2)/\Gamma ((n-k+nh)/2)\prod_{k=1}^{K}\left\{{\left(p_{k}-1\right)}^{\left(p_{k}-1\right)nh/2}\Gamma \left(\left(p_{k}-1\right)(n\;-\;1)/2\right)\;/\;\Gamma \left(\left(p_{k}-1\right)(n\;-\;1+nh)/2\right)\right\}$. We then apply the Box’s approach (Anderson, 2003, Section 8.5) when the sample size n is sufficiently large.

### Proof of Corollary 2.

It follows the results from Jiang et al. (2012, Proposition 2.1).

### Proofs of Corollaries 3 and 4.

The degrees of freedom parameter is calculated directly as the difference in the dimensions of the parameter spaces under the alternative and null hypotheses.

### Proof of Theorem 2.

For simplicity, we suppress the superscript (1). We define $Y≜\sqrt{n}\Gamma \bar{X}$, $v≜\sqrt{n}\Gamma \mu$, $v_{0}≜\sqrt{n}\Gamma \mu_{0}$, and $V≜\Gamma W\Gamma^{⊤}$. Using the second part of Lemma A.5, the likelihood ratio statistic $\Lambda_{2}$ is computed as

$$\Lambda_{2}^{2/n}=det\;\left(V_{00}\right)\;/\;det\;\left(V_{00}+H_{00}\right)\prod_{k=1}^{K}{\left\{tr\;\left(V_{kk}\right)\;/\;tr\;\left(V_{kk}+H_{kk}\right)\right\}}^{p_{k}-1},$$

where $H≜\left(Y-v_{0}\right){\left(Y-v_{0}\right)}^{⊤}∼W_{p}\left(\Xi ,1;\Omega_{H}^{\left(I\right)}\right)$ with $\Omega_{H}^{\left(I\right)}=\left(v-v_{0}\right){\left(v-v_{0}\right)}^{⊤}$. Note that H>0 if $Y\neq v_{0}$, and $H=\left(H_{kk^{\prime }}\right)$ and $\Omega_{H}^{\left(I\right)}=\left(\Omega_{H,kk^{\prime }}^{\left(I\right)}\right)$ are partitioned by a (K+1)-dimensional partition-size vector ${\left(K,p_{1}-1,\;\ldots \;,p_{K}-1\right)}^{⊤}$ for $k,k^{\prime }\in \{0,\;1,\;\ldots \;,K\}$. Furthermore, we define

$$\Lambda_{2}^{\left(0\right)}≜{\left\{det\;\left(V_{00}\right)\;/\;det\;\left(V_{00}+H_{00}\right)\right\}}^{n/2},\;\Lambda_{2}^{\left(k\right)}≜{\left\{tr\;\left(V_{kk}\right)\;/\;tr\;\left(V_{kk}+H_{kk}\right)\right\}}^{\left(p_{k}-1\right)n/2},\;k\in \{1,\;\ldots \;,K\},$$

satisfying $\Lambda_{2}=\prod_{k=0}^{K}\Lambda_{2}^{\left(k\right)}$. By Lemma A.1, we obtain that $H_{00}∼W_{K}\left(\tilde{\Delta },1;\Omega_{H,00}^{\left(I\right)}\right)=W_{K}\left(\tilde{\Delta },1;\Omega_{H,00}^{\left(II\right)}\right)$, which is independent of $V_{00}∼W_{K}(\tilde{\Delta },n\;-\;1)$. Therefore, $\Lambda_{2}^{\left(0\right)}∼\lambda_{2}^{n/2}$ follows a noncentral Wilks lambda distribution $\lambda_{2}∼\Lambda_{K}\left(n-1,1;\Omega_{H,00}^{\left(II\right)}\right)$, where the second type of noncentrality parameter is given by $\Omega_{H}^{\left(II\right)}≜1\times \Xi^{-1}\Omega_{H}^{\left(I\right)}$ (Butler and Wood, 2004; Thu and Phong, 2022). $\Omega_{H}^{\left(II\right)}=\left(\Omega_{H,kk^{\prime }}^{\left(II\right)}\right)$ is partitioned by a (K+1)-dimensional partition-size vector ${\left(K,p_{1}-1,\;\ldots \;,p_{K}-1\right)}^{⊤}$ for $k,k^{\prime }\in \{0,\;1,\;\ldots \;,K\}$. On the other hand, $V_{kk}∼W_{p_{k}-1}\left(a_{kk}I_{p_{k}-1},n\;-\;1\right)$ and $H_{kk}∼W_{p_{k}-1}\left(a_{kk}I_{p_{k}-1},1;\Omega_{H,kk}^{\left(I\right)}\right)$ are independently distributed. Therefore, $tr\;\left(V_{kk}\right)∼a_{kk}\chi^{2}\left((n\;-\;1)\left(p_{k}-1\right)\right)$ and $tr\;\left(H_{kk}\right)∼a_{kk}\chi^{2}\left(p_{k}-1;tr\;\left(\Omega_{H,kk}^{\left(II\right)}\right)\right)$ are independently distributed (Kollo and von Rosen, 2005, Corollary 2.4.2.2). Thus,

$$\Lambda_{2}^{\left(k\right)}∼\beta_{II,2,k}^{\left(p_{k}-1\right)n/2},\;\beta_{II,2,k}∼{Beta}_{II}\left((n\;-\;1)\left(p_{k}-1\right)/2,\left(p_{k}-1\right)/2;tr\;\left(\Omega_{H,kk}^{\left(II\right)}\right)\right),\;k\in \{1,\;\ldots \;,K\},$$

which is called the type II noncentral beta distribution (Chattamvelli, 1995). Alternatively,

$$\Lambda_{2}^{\left(k\right)}∼{\left(1+(n\;-\;1{)}^{-1}F_{2,k}\right)}^{-\left(p_{k}-1\right)n/2},\;F_{2,k}∼F\left(p_{k}-1,(n\;-\;1)\left(p_{k}-1\right);tr\;\left(\Omega_{H,kk}^{\left(II\right)}\right)\right),\;k\in \{1,\;\ldots \;,K\}.$$

Therefore,

$$\Lambda_{2}∼\lambda_{2}^{n/2}\prod_{k=1}^{K}\beta_{II,2,k}^{\left(p_{k}-1\right)n/2}∼\lambda_{2}^{n/2}\prod_{k=1}^{K}{\left(1+(n\;-\;1{)}^{-1}F_{2,k}\right)}^{-\left(p_{k}-1\right)n/2}.$$

Under $H_{2}$, $v=v_{0}$, thus the above noncentrality parameters vanish.

For the second part of Theorem 2, we define $Y^{\left(m\right)}≜\sqrt{n^{\left(m\right)}}\Gamma {\bar{X}}^{\left(m\right)}$, ${\tilde{v}}^{\left(m\right)}≜\Gamma \mu^{\left(m\right)}$, $v^{\left(m\right)}≜\sqrt{n^{\left(m\right)}}{\tilde{v}}^{\left(m\right)}$, and $V^{\left(m\right)}≜\Gamma W^{\left(m\right)}\Gamma^{⊤}$, for m∈{1,…,M}. Using the second part of Lemma A.6, the likelihood ratio statistic $\Lambda_{4}$ is computed as

$$\Lambda_{4}^{2/n}=det\;\left(\sum_{m=1}^{M}V_{00}^{\left(m\right)}\right)\;/\;det\;\left(H_{00}+\sum_{m=1}^{M}V_{00}^{\left(m\right)}\right)\prod_{k=1}^{K}{\left\{tr\;\left(\sum_{m=1}^{M}V_{kk}^{\left(m\right)}\right)\;/\;tr\;\left(H_{kk}+\sum_{m=1}^{M}V_{kk}^{\left(m\right)}\right)\right\}}^{p_{k}-1},$$

where $H≜\sum_{m=1}^{M}\left(Y^{\left(m\right)}-\sqrt{n^{\left(m\right)}}\bar{Y}\right){\left(Y^{\left(m\right)}-\sqrt{n^{\left(m\right)}}\bar{Y}\right)}^{⊤}\in R^{p\times p}$ and $\bar{Y}≜\left(\sum_{m=1}^{M}\sqrt{n^{\left(m\right)}}Y^{\left(m\right)}\right)/n\in R^{p\times 1}$. Additionally, $V=\left(V_{kk^{\prime }}\right)$ and $H=\left(H_{kk^{\prime }}\right)$ are partitioned by a (K+1)-dimensional partition-size vector ${\left(K,p_{1}-1,\;\ldots \;,p_{K}-1\right)}^{⊤}$ for $k,k^{\prime }\in \{0,\;1,\;\ldots \;,K\}$. Furthermore, we define

$$\Lambda_{4}^{\left(0\right)}≜{\left\{det\;\left(\sum_{m=1}^{M}V_{00}^{\left(m\right)}\right)\;/\;det\;\left(H_{00}+\sum_{m=1}^{M}V_{00}^{\left(m\right)}\right)\right\}}^{n/2},\;\Lambda_{4}^{\left(k\right)}≜{\left\{tr\;\left(\sum_{m=1}^{M}V_{kk}^{\left(m\right)}\right)\;/\;tr\;\left(H_{kk}+\sum_{m=1}^{M}V_{kk}^{\left(m\right)}\right)\right\}}^{\left(p_{k}-1\right)n/2},\;k\in \{1,\;\ldots \;,K\},$$

satisfying that $\Lambda_{4}=\prod_{k=0}^{K}\Lambda_{4}^{\left(k\right)}$. Given the above explicit expression of $\Lambda_{4}$ and unstructured ${\tilde{v}}^{\left(m\right)}$, we observe that $Y^{\left(m\right)}∼N_{p}\left(v^{\left(m\right)},\Xi \right)=N_{p}\left(\sqrt{n^{\left(m\right)}}{\tilde{v}}^{\left(m\right)},\Xi \right)$. By the second part of Lemma A.2, $rank\left(A_{Y}\right)=M-1$, where $A_{Y}=I_{M}-{\left(\sqrt{f^{\left(1\right)}},\;\ldots \;,\sqrt{f^{\left(m\right)}}\right)}^{⊤}\left(\sqrt{f^{\left(1\right)}},\;\ldots \;,\sqrt{f^{\left(m\right)}}\right)\in R^{M\times M}$, and the first type of noncentrality parameter is $\Omega_{H}^{\left(I\right)}≜M_{Y}A_{Y}M_{Y}^{⊤}$ with $M_{Y}≜\left(E\left(Y^{\left(1\right)}\right),\;\ldots \;,E\left(Y^{\left(m\right)}\right)\right)=\left(v^{\left(1\right)},\;\ldots \;,v^{\left(m\right)}\right)\in R^{p\times M}$. It is easy to verify the following identity

$$\Omega_{H}^{\left(I\right)}=\sum_{m=1}^{M}n^{\left(m\right)}\left({\tilde{v}}^{\left(m\right)}-\bar{\tilde{v}}\right){\left({\tilde{v}}^{\left(m\right)}-\bar{\tilde{v}}\right)}^{⊤},$$

where $\bar{\tilde{v}}≜\left(n^{\left(1\right)}{\tilde{v}}^{\left(1\right)}+\cdots +n^{\left(m\right)}{\tilde{v}}^{\left(m\right)}\right)/n$ is the pooled mean vector. Therefore, $H∼W_{p}\left(\Xi ,M-1;\Omega_{H}^{\left(I\right)}\right)$, which is independent of $\sum_{m=1}^{M}V^{\left(m\right)}∼W_{p}(\Xi ,n-M)$. By Lemma A.1, we obtain that $H_{00}∼W_{K}\left(\tilde{\Delta },M-1;\Omega_{H,00}^{\left(I\right)}\right)$, which is independent of $\sum_{m=1}^{M}V_{00}^{\left(m\right)}∼W_{K}(\tilde{\Delta },n-M)$. Therefore, $\Lambda_{4}^{\left(0\right)}=\lambda_{4}^{n/2}$ follows a noncentral Wilks lambda distribution $\lambda_{4}∼\Lambda_{K}\left(n-M,M-1;\Omega_{H,00}^{\text{(II)}}\right)$, where the second type of noncentrality parameter is $\Omega_{H}^{\left(II\right)}=(M-1)\Xi^{-1}\Omega_{H}^{\left(I\right)}$ (Butler and Wood, 2004; Thu and Phong, 2022). $\Omega_{H}^{\left(II\right)}=\left(\Omega_{H,kk^{\prime }}^{\left(II\right)}\right)$ is partitioned by a (K+1)-dimensional partition-size vector ${\left(K,p_{1}-1,\;\ldots \;,p_{K}-1\right)}^{⊤}$ for $k,k^{\prime }=0,\;1,\;\ldots \;,K$. On the other hand, $\sum_{m=1}^{M}V_{kk}^{\left(m\right)}∼W_{p_{k}-1}\left(a_{kk}I_{p_{k}-1},n-M\right)$ and $H_{kk}∼W_{p_{k}-1}\left(a_{kk}I_{p_{k}-1},M-1;\Omega_{H,kk}^{\left(I\right)}\right)$ are independently distributed. Therefore, $tr\;\left(\sum_{m=1}^{M}V_{kk}^{\left(m\right)}\right)∼a_{kk}\chi^{2}\left((n-M)\left(p_{k}-1\right)\right)$ and $tr\;\left(H_{kk}\right)∼a_{kk}\chi^{2}\left((M-1)\left(p_{k}-1\right);tr\;\left(\Omega_{H,kk}^{\left(II\right)}\right)\right)$ are independently distributed (Kollo and von Rosen, 2005, Corollary 2.4.2.2). Thus,

$$\Lambda_{4}^{\left(k\right)}∼\beta_{II,4,k}^{\left(p_{k}-1\right)n/2},\;\beta_{II,k}∼{Beta}_{II}\left((n-M)\left(p_{k}-1\right)/2,(M-1)\left(p_{k}-1\right)/2;tr\;\left(\Omega_{H,kk}^{\left(II\right)}\right)\right),\;k\in \{1,\;\ldots \;,K\},$$

which is the type II noncentral beta distribution. Alternatively,

$$\Lambda_{4}^{\left(k\right)}∼{\left\{1+(M-1)/(n-M)F_{4,k}\right\}}^{-\left(p_{k}-1\right)n/2},\;F_{4,k}∼F\left((M-1)\left(p_{k}-1\right),(n-M)\left(p_{k}-1\right);tr\;\left(\Omega_{H,kk}^{\left(II\right)}\right)\right),\;k\in \{1,\;\ldots \;,K\}.$$

Putting them together, we complete the proof. Under $H_{4}$, ${\tilde{v}}^{\left(1\right)}=\cdots ={\tilde{v}}^{\left(m\right)}=\tilde{v}$ and $M_{Y}=\left(\sqrt{n^{\left(1\right)}}\tilde{v},\;\ldots \;,\sqrt{n^{\left(m\right)}}\tilde{v}\right)$. The above noncentrality parameters vanish.

### Proof of Theorem 3.

For the first part of Theorem 3, we suppress the superscript (1).

Step 1: decomposition of $G_{2}$. The following equality demonstrates an elegant way to decompose $G_{2}$:

$$(\hat{A}P{)}^{-1}-{\hat{\Delta }}^{-1}\hat{B}{\hat{A}}^{-1}=(P\hat{\Delta }{)}^{-1}.$$

Define $W_{k}≜W_{k}\left[A_{k},B_{k},p\right]$ for every k, with $A_{k}≜diag\left(0,\;\ldots \;,{\hat{a}}_{kk}^{-1},\;\ldots \;,0\right)$ and $B_{k}≜diag\left(0,\;\ldots \;,-{\hat{a}}_{kk}^{-1}p_{k}^{-1},\;\ldots \;,0\right)$. Herein, $G_{2}$ can be written as a sum of (K+1) components:

$$G_{2}=\sum_{k=1}^{K}n{\left(\bar{X}-\mu_{0}\right)}^{⊤}W_{k}\left(\bar{X}-\mu_{0}\right)+n{\left(\bar{X}-\mu_{0}\right)}^{⊤}\left\{\left(P\hat{\Delta }{)}^{-1}\circ J[p\right]\right\}(\bar{X}-\mu_{0})≜F_{1}+F_{2}+\cdots +F_{K}+F_{K+1},$$

where $F_{k}$ denotes $n{\left(\bar{X}-\mu_{0}\right)}^{⊤}W_{k}(\bar{X}-\mu_{0})$, for k∈{1,…,K}, and $F_{K+1}$ denotes $n{\left(\bar{X}-\mu_{0}\right)}^{⊤}\left\{\left(P\hat{\Delta }{)}^{-1}\circ J[p\right]\right\}(\bar{X}-\mu_{0})$.

Step 2: independence. We have that $F_{1},\;\ldots \;,F_{K},F_{K+1}$ are mutually independent following Lemma A.3.

Step 3: distribution. Decompose $\bar{X}≜{\left({\bar{X}}^{(1),T},\;\ldots \;,{\bar{X}}^{(K),T}\right)}^{⊤}$ and $\mu_{0}≜{\left(\mu_{0}^{(1),T},\;\ldots \;,\mu_{0}^{(K),T}\right)}^{⊤}$, where ${\bar{X}}^{\left(k\right)},\mu_{0}^{\left(k\right)}\in R^{p_{k}}$ for every k. We have

$$F_{k}=n{\left(\bar{X}-\mu_{0}\right)}^{⊤}W_{k}(\bar{X}-\mu_{0})={\left({\bar{X}}^{\left(k\right)}-\mu_{0}^{\left(k\right)}\right)}^{⊤}\left(n{\tilde{a}}_{kk}^{-1}I_{p_{k}}-n{\tilde{a}}_{kk}^{-1}p_{k}^{-1}J_{p_{k}}\right)({\bar{X}}^{\left(k\right)}-\mu_{0}^{\left(k\right)}),\;k\in \{1,\;\ldots \;,K\}.$$

Let $U_{k}≜n{\left({\bar{X}}^{\left(k\right)}-\mu_{0}^{\left(k\right)}\right)}^{⊤}\left\{\left(1/a_{kk}\right)I_{p_{k}}-1/\left(a_{kk}p_{k}\right)J_{p_{k}}\right\}({\bar{X}}^{\left(k\right)}-\mu_{0}^{\left(k\right)})$ and $V_{k}≜\left(p_{k}-1\right)(n\;-\;1)\left({\overset{ˆ}{a}}_{kk}/a_{kk}\right)$ for every k. Thus, $U_{k}/V_{k}=F_{k}/\left\{\left(p_{k}-1\right)(n\;-\;1)\right\}$ for every k, where $U_{k}∼\chi^{2}\left(p_{k}-1;\delta_{k}\right)$ with the noncentrality parameter

$$\delta_{k}={\left(\mu^{\left(k\right)}-\mu_{0}^{\left(k\right)}\right)}^{⊤}\left(a_{kk}^{-1}I_{p_{k}}-a_{kk}^{-1}p_{k}^{-1}J_{p_{k}}\right)(\mu^{\left(k\right)}-\mu_{0}^{\left(k\right)}),$$

by Lemma A.4, and $a_{kk}V_{k}∼\chi^{2}\left((n\;-\;1)\left(p_{k}-1\right)\right)$, for k∈{1,…,K}.

Given $F_{K+1}=n{\left(\bar{X}-\mu_{0}\right)}^{⊤}\left\{\left(P\hat{\Delta }{)}^{-1}\circ J[p\right]\right\}(\bar{X}-\mu_{0})$, consider transformations $Y=C_{p}X,Y_{i}=C_{p}X_{i}$, for i∈{1,…,n}, and $\bar{Y}=C_{p}\bar{X}$, where $C_{p}≜Bdiag\left(1_{1\times p_{1}}/p_{1},\;\ldots \;,1_{1\times p_{K}}/p_{K}\right)\in R^{K\times p}$. As $X∼N_{p}(\mu ,\Sigma [A,B,p])$, we obtain that $Y∼N_{K}\left(\mu_{Y},\Sigma_{Y}\right)$, where $\mu_{Y}≜C_{p}\mu$ and $\Sigma_{Y}≜C_{p}\Sigma [A,B,p]C_{p}^{⊤}=AP^{-1}+B=\Delta P^{-1}$. Let $v_{0}≜C_{p}\mu_{0}={\left(1_{1\times p_{1}}\mu_{0}^{\left(1\right)}/p_{1},\;\ldots \;,1_{1\times p_{K}}\mu_{0}^{\left(K\right)}/p_{K}\right)}^{⊤}$. Noting that ${\left({PC}_{p}\right)}^{⊤}\Gamma \left({PC}_{p}\right)=\Gamma \circ J[p]$ for any $\Gamma \in R^{K\times K}$, then,

$$F_{K+1}=n{\left(\bar{X}-\mu_{0}\right)}^{⊤}\left\{\left(P\hat{\Delta }{)}^{-1}\circ J[p\right]\right\}(\bar{X}-\mu_{0})=n{\left(\bar{X}-\mu_{0}\right)}^{⊤}\left\{{\left({PC}_{p}\right)}^{⊤}(P\hat{\Delta }{)}^{-1}\left(PC_{p}\right)\right\}\left(\bar{X}-\mu_{0}\right)=n{\left(\bar{Y}-v_{0}\right)}^{⊤}\left({\hat{\Sigma }}_{Y}^{-1}\right)(\bar{Y}-v_{0}),$$

where $P{\hat{\Delta }}^{-1}=P{\left(\hat{A}P^{-1}P+\hat{B}P\right)}^{-1}=PP^{-1}{\left(\hat{A}P^{-1}+\hat{B}\right)}^{-1}={\hat{\Sigma }}_{Y}^{-1}$. Since $\sum_{i=1}^{n}{\left(Y_{i}-\bar{Y}\right)}^{⊤}\left(Y_{i}-\bar{Y}\right)/(n\;-\;1)={\hat{\Sigma }}_{Y}$, $\sqrt{n}\left(\bar{Y}-v_{0}\right)∼N_{K}(\sqrt{n}\left(\mu_{Y}-v_{0}\right),\Sigma_{Y})$, and they are independent, we conclude that

$$F_{K+1}=T^{2}∼K(n\;-\;1)/(n-K)F\left(K,n-K;\delta_{K+1}\right),$$

where $\delta_{K+1}≜n{\left(\mu_{Y}-v_{0}\right)}^{⊤}\Sigma_{Y}^{-1}\left(\mu_{Y}-v_{0}\right)$ (Muirhead, 2005, Theorem 6.3.1). Under $H_{2}$, the above noncentrality parameters vanish.

The second part of Theorem 3 can be proved using similar arguments.

### Proof of Corollary 5.

This result follows directly from Geisser (1963, pages 663, 666).

### Proof of Theorem 4.

We verify the conditions of Theorem 2 and Theorem 3 in Fan and Han (2017). Specifically, given the existence of $\delta_{1}$, $\delta_{2}$, and $\delta_{3}$, (condition 1), (condition 2), (condition 5) in Fan and Han (2017) are satisfied. Thus, it remains to verify (condition 3) and (condition 4), which is equivalent to establishing the existence of $\delta_{4}>0$ such that $\left\|\hat{\Psi }\left[{\hat{A}}_{\Psi },{\hat{B}}_{\Psi },p\right]-\Psi \left[A_{\Psi },B_{\Psi },p\right]\right\|=O\left(g_{p}p^{-\delta_{4}}\right)$. Using the modified hard-thresholding estimators (Yang et al., 2024), the proposed correlation matrix estimator satisfies

$$Pr\left\{\left\|\hat{\Psi }\left[{\hat{A}}_{\Psi },{\hat{B}}_{\Psi },p\right]-\Psi \left[A_{\Psi },B_{\Psi },p\right]\right\|\leq C_{q}(2\lambda {)}^{1-q}\right\}\to 1$$

as K, p→∞, log(K)/n→0, where $\lambda ≜\eta \sqrt{log(K)/n}$, and 0<q<1, $C_{q}≜C_{q}(K)$, and η are constants. Thus, we can express this as

$$\left\|\hat{\Psi }\left[{\hat{A}}_{\Psi },{\hat{B}}_{\Psi },p\right]-\Psi \left[A_{\Psi },B_{\Psi },p\right]\right\|=O_{P}\left[C_{q}(K)\{log(K)/n{\}}^{(1-q)/2}\right].$$

This result is consistent with the sparse matrix case (Bickel and Levina, 2008b), and the existence of $\delta_{4}$ has been established (Fan and Han, 2017).

## Appendix C. Supplementary data

Supplementary material related to this article can be found online at https://doi.org/10.1016/j.jmva.2026.105641. The online supplementary material contains the Simulation Studies and the Real Data Analysis.
