# Supplementary material for: Hypothesis testing under uniform-block covariance structures
Source: J Multivar Anal. Author manuscript; Available in PMC 2026 Sep 12. (PMC13564209; doi:10.1016/j.jmva.2026.105641)
Supplement: Supplementary file 1 [file NIHMS2206487-supplement-Supplementary_Material.pdf]

Supplementary material for *Hypothesis testing under uniform-block covariance structures*

Yifan Yang
Department of Population and Quantitative Health Sciences,
Case Western Reserve University, Cleveland, Ohio, 44106 U.S.A.

Shuo Chen *
Department of Epidemiology and Public Health,
University of Maryland School of Medicine, Baltimore, Maryland, 21201 U.S.A.

Ming Wang *
Department of Population and Quantitative Health Sciences,
Case Western Reserve University, Cleveland, Ohio, 44106 U.S.A.

Abstract

The present supplementary material is organized into two sections. Section A presents the simulation studie, and Section B presents the real data analysis. The R code is accessible at https://github.com/yiorfun/UB.

A SIMULATION STUDIES

A.1 Study 1: LRT Statistics in Scenario 1

This study presents a numerical comparison of the empirical distributions of $-\log \Lambda_1$ and $-\log \Lambda_3$ with their corresponding null theoretical and approximate distributions. Theoretical and approximate results are given in Theorem 1 (LRT statistics related to covariance structures) and the associated corollaries. The comparison is carried out across a range of sample sizes, covariance matrix dimensions, and population numbers using Monte Carlo methods.

*Emails: yxy1423@case.edu; shuochen@som.umaryland.edu; mxw827@case.edu; and * is for the corresponding author

For $M = 1$, the true population mean vector and covariance matrix are given by $\boldsymbol{\mu}^{(1)} = \boldsymbol{\mu}_*$ and $\boldsymbol{\Sigma}^{(1)}\left[\mathbf{A}^{(1)}, \mathbf{B}^{(1)}, \boldsymbol{p}\right]$, respectively, where $\boldsymbol{p} = (p_1, p_2, p_3, p_4, p_5)^\top = (\lfloor 3\rho \rfloor, \lfloor 4\rho \rfloor, \lfloor 5\rho \rfloor, \lfloor 6\rho \rfloor, \lfloor 7\rho \rfloor)^\top$. Thus, the null hypothesis H_1 holds. Here, $\rho \in \mathbb{R}^+$ controls the dimension p, $\lfloor \cdot \rfloor$ denotes the floor function, and $K = 5$, where $p = \sum_{k=1}^{5} \lfloor k\rho \rfloor$. Additionally, $\boldsymbol{\mu}_* = \mathbf{1}_{p\times 1}$, $\mathbf{A}^{(1)} = \mathbf{A}_*$, $\mathbf{B}^{(1)} = \mathbf{B}_*$, $\mathbf{P} = \mathrm{diag}\left(\lfloor 3\rho \rfloor, \lfloor 4\rho \rfloor, \lfloor 5\rho \rfloor, \lfloor 6\rho \rfloor, \lfloor 7\rho \rfloor\right)$, and

$$\mathbf{A}_* = \begin{pmatrix} 0.016 & & & & \\ & 0.214 & & & \\ & & 0.749 & & \\ & & & 0.068 & \\ & & & & 0.100 \end{pmatrix}, \quad \mathbf{B}_* = \begin{pmatrix} 6.731 & -1.690 & 0.696 & -2.936 & 1.913 \\ -1.690 & 5.215 & 3.815 & -1.010 & 0.703 \\ 0.696 & 3.815 & 4.328 & -3.357 & -0.269 \\ -2.936 & -1.010 & -3.357 & 6.788 & 0.000 \\ 1.913 & 0.703 & -0.269 & 0.000 & 3.954 \end{pmatrix},$$

$$\boldsymbol{\Delta}^{(1)} = \begin{pmatrix} 20.21 & -6.761 & 3.480 & -17.62 & 13.39 \\ -5.071 & 21.07 & 19.08 & -6.060 & 4.921 \\ 2.088 & 15.26 & 22.39 & -20.14 & -1.882 \\ -8.809 & -4.040 & -16.79 & 40.80 & 0.001 \\ 5.739 & 2.812 & -1.345 & 0.001 & 27.78 \end{pmatrix}, \quad \widetilde{\boldsymbol{\Delta}}^{(1)} = \begin{pmatrix} 20.21 & -5.856 & 2.695 & -12.46 & 8.767 \\ -5.856 & 21.07 & 17.06 & -4.949 & 3.720 \\ 2.695 & 17.06 & 22.39 & -18.39 & -1.591 \\ -12.46 & -4.949 & -18.39 & 40.79 & 0.001 \\ 8.767 & 3.720 & -1.591 & 0.001 & 27.78 \end{pmatrix}.$$

There exists a p by p orthonormal matrix $\boldsymbol{\Gamma}$, determined by (1) in the manuscript, such that $\boldsymbol{\Xi}^{(1)} \triangleq \boldsymbol{\Gamma}\boldsymbol{\Sigma}^{(1)}\left[\mathbf{A}^{(1)}, \mathbf{B}^{(1)}, \boldsymbol{p}\right]\boldsymbol{\Gamma}^\top = \mathrm{Bdiag}(\widetilde{\boldsymbol{\Delta}}^{(1)}, 0.016 \times \mathbf{I}_{p_1-1}, 0.214 \times \mathbf{I}_{p_2-1}, 0.749 \times \mathbf{I}_{p_3-1}, 0.068 \times \mathbf{I}_{p_4-1}, 0.100 \times \mathbf{I}_{p_5-1})$.

Monte Carlo simulations with 10^5 replicates are conducted to compare the theoretical, approximate, and empirical distributions of the negative logarithm of the LRT statistic, $-\log \Lambda_1$, under the null hypothesis H_1. Specifically, for each replicate, we adjust $\boldsymbol{p}$ by varying the values of ρ and generate an i.i.d. sample of size $n = n^{(1)} \in \{50, 100\}$ from $N_p\left(\boldsymbol{\mu}^{(1)}, \boldsymbol{\Sigma}^{(1)}\left[\mathbf{A}^{(1)}, \mathbf{B}^{(1)}, \boldsymbol{p}\right]\right)$. Using the known $\boldsymbol{\Gamma}$, we then calculate $-\log \Lambda_1$ based on the result in the first part of Theorem 1 (LRT statistics related to covariance structures). This procedure yields the empirical distribution (denoted as "Emp") of $-\log \Lambda_1$ over 10^5 replicates. The theoretical distribution (denoted as "The") of $-\log \Lambda_1$ is also obtained numerically using the same theorem, based on 10^5 replicates. Additionally, for each replicate, we generate three approximate distributions of $-\log \Lambda_1$, as described in Corollary 1 (large-sample Box approximation for Λ_1) (denoted as "Box-App"), Corollary 2 (high-dimensional normal approximation for Λ_1) (denoted as "Normal-App"), and Corollary 3 (large-sample Wilks approximation for Λ_1 and Λ_3) (denoted as "ChiSq-App"), respectively. The quantiles of these distributions are summarized in Table A.1.

For $M > 1$, we consider the case of $M = 3$ populations. The partition-size vector $\boldsymbol{p}$, true population mean vectors, and covariance matrices are identical to those in the case of $M = 1$. Specifically, $\boldsymbol{p} = (\lfloor 3\rho \rfloor, \lfloor 4\rho \rfloor, \lfloor 5\rho \rfloor, \lfloor 6\rho \rfloor, \lfloor 7\rho \rfloor)^\top$, where ρ varies across different values. The mean vec-

tors are given by $\boldsymbol{\mu}^{(m)} = m\boldsymbol{\mu}_*$, and the covariance matrices take the form $\boldsymbol{\Sigma}^{(m)}\left[\mathbf{A}^{(m)}, \mathbf{B}^{(m)}, \boldsymbol{p}\right]$, where $\mathbf{A}^{(m)} = \mathbf{A}_*$ and $\mathbf{B}^{(m)} = \mathbf{B}_*$ for $m \in \{1, \ldots, M\}$. Thus, the null hypothesis H_3 holds. Additionally, in hypothesis H_3, the common covariance matrix $\boldsymbol{\Sigma}\left[\mathbf{A}, \mathbf{B}, \boldsymbol{p}\right]$ has $\mathbf{A} = \mathbf{A}_*$ and $\mathbf{B} = \mathbf{B}_*$. Consequently, its canonical form remains the same as in the case of $M = 1$, satisfying that $\boldsymbol{\Xi} = \boldsymbol{\Gamma}\boldsymbol{\Sigma}\left[\mathbf{A}, \mathbf{B}, \boldsymbol{p}\right]\boldsymbol{\Gamma}^\top$ with the same matrix $\boldsymbol{\Gamma}$.

Similarly, under the null hypothesis H_3, we compare the theoretical, approximate, and empirical distributions of the negative logarithm LRT statistic, $-\log\Lambda_3$, using Monte Carlo simulations with 10^5 replicates. Specifically, for each replicate, we generate i.i.d. samples from $N_p\left(\boldsymbol{\mu}^{(m)}, \boldsymbol{\Sigma}^{(m)}\left[\mathbf{A}^{(m)}, \mathbf{B}^{(m)}, \boldsymbol{p}\right]\right)$ for $m \in \{1, 2, 3\}$ with sizes $\left(n^{(1)}, n^{(2)}, n^{(3)}\right) \in \{(50, 50, 50), (50, 75, 100)\}$. We then apply formula (3) in the manuscript to every $\mathbf{S}^{(m)}$. Using the known matrix $\boldsymbol{\Gamma}$, we derive numerically the theoretical and empirical distributions of $-\log\Lambda_3$ according to the second part of Theorem 1 (LRT statistics related to covariance structures), denoted as "The" and "Emp", respectively. The approximate distribution, given in Corollary 3 (large-sample Wilks approximation for Λ_1 and Λ_3), is also included and denoted as "ChiSq-App". The quantiles of these distributions are summarized in Table A.1.

These results demonstrate that the null theoretical and approximate distributions closely align with their null empirical distributions, as evidenced by the remarkably similar quantiles. For $-\log\Lambda_1$, "Normal-App" performs well even when the dimension p is comparable to the sample size $n^{(1)}$, whereas the "Box-App" and "ChiSq-App" provide reliable results only when the sample size substantially exceeds the dimension. Accordingly, the result for "Box-App" is reported only for the case when $n^{(1)} = 50$ and $p = 25$. Additionally, we observe that the quantiles of $-\log\Lambda_3$ are similar across various sample sizes and dimensions. It is because the parameters for the Dirichlet variates are relatively large in comparison to the sample sizes.

Table A.1: Quantiles of null theoretical, approximate, and empirical distributions related to $-\log \Lambda_1$ and $-\log \Lambda_3$ under various sample sizes $n^{(1)}$ or $(n^{(1)}, n^{(2)}, n^{(3)})$ and covariance matrix dimensions p, where "The" and "Emp" represent the theoretical and empirical distributions, "Box-App", "ChiSq-App" and "Normal-App" represent the Box, χ^2, and normal approximate distributions.

Statistic	Sample Size	Dimension	Distribution	Quantile						
				0.01	0.05	0.10	0.50	0.90	0.95	0.99
$-\log \Lambda_1$	50	25	The	159.82	169.49	174.84	194.66	215.90	222.01	234.23
			Box-App	132.99	150.38	159.93	193.92	229.90	240.70	260.61
			Normal-App	158.71	169.33	174.97	195.18	215.32	221.06	231.69
			ChiSq-App	125.32	132.89	137.02	152.21	168.46	173.38	182.56
			Emp	159.84	169.65	174.95	194.61	215.90	222.33	234.40
		45	The	792.44	820.77	836.43	893.49	954.68	972.64	1007.15
			Normal-App	791.69	821.83	837.90	894.92	951.67	967.93	998.32
			ChiSq-App	457.00	470.97	478.83	507.15	536.54	545.14	561.31
			Emp	792.94	821.45	836.65	893.56	953.90	972.10	1007.13
	100	75	The	1942.13	1979.90	2000.40	2073.99	2149.85	2171.50	2212.52
			Normal-App	1942.08	1980.43	2001.21	2074.25	2147.46	2168.48	2207.96
			ChiSq-App	1328.71	1353.59	1367.00	1414.90	1463.86	1477.95	1503.56
			Emp	1942.67	1980.77	2001.17	2073.72	2149.30	2170.96	2211.20
		95	The	3931.43	4001.59	4039.75	4174.92	4316.69	4358.35	4435.73
			Normal-App	3932.67	4004.63	4043.27	4178.46	4315.03	4354.18	4425.45
			ChiSq-App	2160.80	2192.84	2209.73	2269.69	2331.10	2348.76	2382.69
			Emp	3934.19	4002.40	4040.18	4175.50	4317.80	4360.04	4438.28
$-\log \Lambda_3$	50, 50, 50	200	The	11.747	13.994	15.398	20.833	27.453	29.574	33.777
			ChiSq-App	11.117	13.288	14.542	19.653	25.889	27.841	31.888
			Emp	11.719	14.003	15.371	20.806	27.443	29.510	33.667
		400	The	11.789	14.064	15.396	20.823	27.454	29.554	33.855
			ChiSq-App	11.086	13.225	14.522	19.640	25.887	27.863	31.811
			Emp	11.683	14.000	15.367	20.841	27.471	29.579	33.754
	50, 75, 100	200	The	11.655	13.924	15.277	20.697	27.234	29.333	33.460
			ChiSq-App	11.079	13.223	14.507	19.652	25.899	27.918	31.926
			Emp	11.680	13.973	15.313	20.711	27.250	29.327	33.492
		400	The	11.773	14.068	15.398	20.875	27.487	29.570	33.779
			ChiSq-App	11.127	13.246	14.499	19.658	25.842	27.840	31.770
			Emp	11.725	14.034	15.402	20.858	27.427	29.525	33.819

A.2 Study 2: LRT and Geisser's Information Statistics in Scenario 2

We employ Monte Carlo simulations to numerically compare the theoretical, approximate, and empirical distributions of $-\log \Lambda_2$, G_2, $-\log \Lambda_4$, and G_4. Various sample sizes, dimensions of covariance matrices, and numbers of populations are considered. The statistics and their distributions are provided in Theorem 2 (LRT statistics related to mean vectors), Theorem 3 (Geisser's information statistics related to mean vectors), and the associated corollaries and remarks.

For $M = 1$, we set $\boldsymbol{\mu}_0 = 2\boldsymbol{\mu}_*$, while keeping the remaining configurations the same as in Study 1. As a result, $\boldsymbol{\mu}^{(1)} \neq \boldsymbol{\mu}_0$, implying the alternative hypothesis K_2 holds and the noncentrality parameters are non-zero. Following a procedure similar to that in Study 1, we compare the theoretical (denoted as "Beta-The" and "F-The" for the β- and F-related distributions), ap-

proximate, and empirical distributions of $-\log \Lambda_2$ and G_2. For $M > 1$, we set $M = 3$ while maintaining the same configurations as in Study 1. This ensures that $\boldsymbol{\mu}^{(1)} \neq \cdots \neq \boldsymbol{\mu}^{(M)}$ for $m \in \{1, \ldots, M\}$ and the alternative hypothesis K_4 holds, resulting in non-zero noncentrality parameters. We then compare the theoretical, approximate (denoted as "F-App" for the F-type distribution), and empirical distributions of $-\log \Lambda_4$ and G_4.

The quantiles of these distributions are summarized in Table A.2. These results indicate that the numerically derived theoretical and approximate distributions are closely aligned with the empirical ones.

Table A.2: Quantiles of alternative theoretical, approximate, and empirical distributions related to $-\log \Lambda_2$, G_2, $-\log \Lambda_4$, and G_4 under various sample sizes $n^{(1)}$ or $(n^{(1)}, n^{(2)}, n^{(3)})$ and covariance matrix dimensions p, where "The", "Beta-The", "F-The", and "Emp" represent the theoretical, β- and F-related theoretical, and empirical distributions, and "F-App" represents the F-type approximate distribution

Statistic	Sample Size	Dimension	Distribution	Quantile						
				0.01	0.05	0.10	0.50	0.90	0.95	0.99
$-\log \Lambda_2$	50	200	Beta-The	123.327	130.177	133.985	148.022	162.934	167.353	175.798
			F-The	123.303	130.351	134.124	147.992	163.051	167.465	175.998
			Emp	123.308	130.278	134.135	148.145	163.036	167.578	175.854
		400	Beta-The	220.413	229.944	235.166	254.114	273.985	279.694	291.080
			F-The	220.466	230.043	235.075	253.919	273.912	279.830	291.117
			Emp	220.394	229.950	234.982	254.052	274.012	279.845	290.972
	100	200	Beta-The	167.034	174.638	178.871	194.037	210.173	214.960	224.151
			F-The	166.874	174.591	178.848	194.032	210.268	214.816	223.883
			Emp	167.249	174.783	178.857	194.107	210.257	215.041	224.231
		400	Beta-The	269.3953	279.3113	284.7258	304.7685	325.6813	331.9213	343.4398
			F-The	269.2884	279.3946	284.8616	304.6875	325.6809	331.5823	343.0884
			Emp	269.4868	279.2633	284.7421	304.5777	325.6015	331.6315	343.3227
G_2	50	200	The	363.554	398.164	417.599	502.407	621.793	663.238	755.674
			Emp	364.475	398.230	418.419	503.561	621.576	663.432	756.562
		400	The	608.900	652.128	676.853	782.606	928.630	980.872	1093.590
			Emp	607.257	650.738	675.778	782.244	928.989	980.425	1095.708
	100	200	The	583.338	632.566	660.423	773.837	918.943	966.430	1065.677
			Emp	583.754	633.030	660.681	774.566	918.228	966.582	1068.722
		400	The	887.8168	948.3858	983.729	1125.463	1302.386	1360.822	1487.144
			Emp	888.9199	949.3287	984.214	1125.168	1303.548	1362.022	1485.925
$-\log \Lambda_4$	50, 50, 50	200	Beta-The	281.763	292.133	297.880	318.657	340.347	346.874	359.048
			F-The	281.594	292.156	297.733	318.623	340.324	346.690	358.708
			Emp	281.229	291.834	297.802	318.645	340.532	346.850	358.708
		400	Beta-The	485.561	499.869	507.721	535.237	563.825	572.285	588.060
			F-The	485.722	499.998	507.412	535.216	564.217	572.573	588.062
			Emp	485.353	500.074	507.562	535.343	564.076	572.250	588.618
	50, 75, 100	200	Beta-The	328.943	340.243	346.311	368.583	391.673	398.333	411.144
			F-The	329.095	340.406	346.482	368.687	391.641	398.360	410.985
			Emp	328.602	340.277	346.537	368.518	391.617	398.250	411.313
		400	Beta-The	539.792	554.690	562.471	591.034	620.571	629.281	645.608
			F-The	539.818	554.420	562.509	591.159	620.894	629.553	646.051
			Emp	539.341	554.386	562.479	591.088	620.883	629.566	645.908
G_4	50, 50, 50	200	The	788.881	835.757	862.111	966.345	1089.470	1130.437	1211.448
			F-App	828.570	868.187	889.878	970.393	1057.824	1084.427	1134.842
			Emp	789.131	835.519	861.782	965.595	1089.195	1128.819	1208.561
		400	The	1294.486	1353.808	1386.375	1514.127	1667.153	1715.710	1812.950
			F-App	1345.656	1394.111	1420.460	1519.227	1625.179	1656.667	1717.360
			Emp	1292.841	1351.560	1385.119	1514.584	1665.860	1713.583	1810.781
	50, 75, 100	200	The	975.567	1030.077	1060.282	1177.183	1312.755	1355.591	1440.176
			F-App	1024.231	1067.407	1091.444	1180.998	1277.502	1306.167	1364.009
			Emp	975.234	1029.516	1060.182	1177.412	1313.147	1355.722	1440.607
		400	The	1530.202	1598.158	1635.924	1781.462	1947.555	1998.722	2103.913
			F-App	1592.419	1647.910	1676.652	1785.270	1901.677	1934.882	2001.965
			Emp	1532.363	1598.074	1635.684	1780.707	1946.535	1998.482	2103.748

A.3 Study 3: Simultaneous Marginal Tests in Scenario 3

We evaluate the proposed FDP estimation procedure for multiple marginal tests through Monte Carlo approaches.

Given a single population, we fix $n = n^{(1)} = 100$ and set $\boldsymbol{p} = \left(\lfloor 3\rho \rfloor, \lfloor 4\rho \rfloor, \lfloor 5\rho \rfloor, \lfloor 6\rho \rfloor, \lfloor 7\rho \rfloor\right)^\top$, varying ρ over a predefined set of values $\rho \in \{12, 24\}$. The true population covariance matrix is defined as $\boldsymbol{\Sigma}^{(1)}\left[\mathbf{A}^{(1)}, \mathbf{B}^{(1)}, \boldsymbol{p}\right]$, where $\mathbf{A}^{(1)} = \mathbf{A}_*$ and $\mathbf{B}^{(1)} = \mathbf{B}_*$, consistent with Study 1. The population mean is designed as $\boldsymbol{\mu}^{(1)} = \boldsymbol{\mu}_\star$, where $\boldsymbol{\mu}_\star$ is constructed as follows. First, we define the effect size $\kappa_{\boldsymbol{\mu}} \in \{0.5, 1.0, 1.5\}$ as the mean over its standard deviation. Second, for each value of $\kappa_{\boldsymbol{\mu}}$, we set $\mu_{\star,\bar{p}_{k-1}+1} = \mu_{\star,\bar{p}_{k-1}+2} = \mu_{\star,\bar{p}_{k-1}+3} = \kappa_{\boldsymbol{\mu}} \times \sqrt{c_{kk}}/\sqrt{n}$ for $k \in \{1, \ldots, K\}$ and $\mu_{\star,j} = 0$ otherwise. That is, only the first three mean components are non-zero in each sub-block of the covariance structure. Thus, the total number of non-zero mean components is $3K = 15$, implying there are 15 false null hypotheses among all p hypotheses.

We conduct 10^3 Monte Carlo replicates in this study. In each replicate, we generate $n^{(1)} = 100$ normal observations from $N_p\left(\boldsymbol{\mu}^{(1)}, \boldsymbol{\Sigma}^{(1)}\left[\mathbf{A}^{(1)}, \mathbf{B}^{(1)}, \boldsymbol{p}\right]\right)$ for each combination of ρ and $\kappa_{\boldsymbol{\mu}}$. We then apply the proposed FDP estimation procedure as described in the previous section. Specifically, we fix $\alpha = 0.05$ and select κ_0 using Fan and Han (2017)'s approach. The estimates $\widehat{\mathbf{A}}^{(1)}$, $\widehat{\mathbf{B}}^{(1)}$, $\widehat{\mathbf{C}}^{(1)}$, and $\widehat{\mathbf{D}}^{(1)}$ are computed using (3) in the manuscript. Next, we construct the marginal test vector $\boldsymbol{T} \triangleq \sqrt{n}\widehat{\mathbf{D}}^{(1),-1}\bar{\boldsymbol{X}}$ and compute the unadjusted p-values P_j using (7) in the manuscript. The estimated correlation matrix $\widehat{\boldsymbol{\Psi}}^{(1)}\left[\widehat{\mathbf{A}}_{\boldsymbol{\Psi}}^{(1)}, \widehat{\mathbf{B}}_{\boldsymbol{\Psi}}^{(1)}, \boldsymbol{p}\right]$ is also determined. We consider a candidate set of 200 threshold values, ranging from $\exp(-2)$ to $\exp(-7)$. For each threshold t_s, we estimate the realized $\mathrm{FDP}\left(t_s\right)$ and compute the adjusted p-values $\widehat{P}_j$ for $j \in \{1, \ldots, p\}$ using the build-in functions of the R package `pfa` (Fan et al., 2012). The FDP estimate at t_s is denoted as "PFA-UB". For comparison, we also evaluate two additional covariance matrix estimators for the marginal test statistic $\sqrt{n}\bar{\boldsymbol{X}}$: "PFA-True", the true covariance matrix $\boldsymbol{\Sigma}^{(1)}\left[\mathbf{A}^{(1)}, \mathbf{B}^{(1)}, \boldsymbol{p}\right]$, and "PFA-POET", the principal orthogonal complement thresholding (POET) estimator from the R package `POET` (Fan et al., 2011). For each t_s, we compute the number of true discoveries, $\widehat{S}(t_s)$, modified from the build-in functions from Vutov and Dickhaus (2023a,b), for each estimator when the corresponding adjusted p-values $\widehat{P}_j$ satisfy $\widehat{P}_j \leq t_s$. Across 10^3 replicates, we calculate the median and standard error of $\widehat{\mathrm{FDP}}(t_s)$ and the average and standard error of $\widehat{S}(t_s)$ for "PFA-True", "PFA-UB", and "PFA-POET", respectively. Given $\alpha = 0.05$, we report t_α in Table A.3 for each estimator. The complete results for all 200 threshold values are provided in the online Excel file.

Based on these results, "PFA-UB" exhibits greater similarity to "PFA-True" than "PFA-

POET", as it better captures the underlying covariance structure. Specifically, "PFA-POET" yields a relatively higher estimated FDP than "PFA-True", resulting in a smaller t_α based on the same candidate threshold values under the same nominal level α. As the number of tests p and effect sizes $\kappa_{\boldsymbol{\mu}}$ increase, the performances of "PFA-UB" and "PFA-POET" become increasingly similar. Notably, the standard error of $\widehat{\mathrm{FDP}}(t)$ is relatively large compared to its median, suggesting that FDP values are not tightly concentrated around the $\mathrm{FDR}(t)$ due to strong correlation.

Table A.3: Results of the estimated FDP and t_α for various methods under various sample sizes $n^{(1)}$, covariance matrix dimensions p, and "Effect size" $\kappa_{\boldsymbol{\mu}}$, where "PFA-POET", "PFA-True", and "PFA-UB" refer to methods utilizing the POET covariance matrix estimator, the true covariance matrix, and the proposed covariance matrix estimator, respectively. Here "t_α" denotes the largest threshold satisfying $\widehat{\mathrm{FDP}}(t_\alpha) \leq \alpha$ and "S.E." refers to the standard error

Dimension	Effect Size	Method	t_α	$R(t)$		$S(t)$		$\widehat{\mathrm{FDP}}(t)$	
				Mean	S.E.	Mean	S.E.	Mean	S.E.
		PFA-POET	0.0016	55.1000	54.6436	4.3080	3.2012	0.0493	0.3477
	0.5	PFA-True	0.0548	17.9980	32.6748	11.5120	1.2391	0.0431	0.4412
		PFA-UB	0.0521	17.1410	31.7714	9.5470	2.7795	0.0346	0.4507
		PFA-POET	0.0018	58.6940	54.0670	8.1440	3.0946	0.0498	0.3395
300	1.0	PFA-True	0.0495	17.7250	30.6826	14.1440	0.7745	0.0444	0.4175
		PFA-UB	0.0405	14.5870	27.4518	13.2960	1.7537	0.0471	0.4218
		PFA-POET	0.0032	68.7660	54.9925	11.3980	2.6104	0.0500	0.3491
	1.5	PFA-True	0.0521	20.7920	31.3039	14.9120	0.2938	0.0442	0.4068
		PFA-UB	0.0415	16.9400	27.6478	14.5140	1.1319	0.0466	0.4097
		PFA-POET	0.0011	94.9220	101.8203	3.8760	3.0196	0.0492	0.3546
	0.5	PFA-True	0.0534	29.7710	59.0493	11.4980	1.2367	0.0379	0.4474
		PFA-UB	0.0508	28.2870	56.7161	9.5700	2.8434	0.0443	0.4645
		PFA-POET	0.0011	95.7850	100.1713	7.7880	3.1011	0.0498	0.3360
600	1.0	PFA-True	0.0357	20.8820	47.2782	14.0280	0.8221	0.0033	0.4007
		PFA-UB	0.0376	22.1970	48.4059	13.2960	1.7741	0.0392	0.4309
		PFA-POET	0.0018	111.3730	104.7553	11.0650	2.6393	0.0491	0.3460
	1.5	PFA-True	0.0508	31.8240	56.5126	14.9180	0.3023	0.0486	0.4076
		PFA-UB	0.0348	22.6660	46.0926	14.5340	1.1358	0.0474	0.4088

A.4 Study 4: Robustness Analysis for the Proposed Tests

In this section, we conduct a robustness analysis of the proposed test statistics. Specifically, we assess their robustness in terms of empirical statistical powers and sizes under three scenarios: (1) random noise that disrupts the uniform-block structures for $-\log\Lambda_1$, $-\log\Lambda_2$, G_2, $-\log\Lambda_3$, $-\log\Lambda_4$, and G_4; (2) random missingness that leads to incomplete sample covariance matrices for G_2 and G_4; and (3) distributional misspecification that violates the normality assumption (e.g., multivariate t-distributions with heavy tails) for $-\log\Lambda_2$, G_2, $-\log\Lambda_4$, and G_4.

We begin with the same simulation setup as in Study 1 for both $M = 1$ and $M = 3$, except for the designs of population means and covariance matrices. Specifically, we fix $n = n^{(1)} = 50$

for $M = 1$ and $n = 150$, $\left(n^{(1)}, n^{(2)}, n^{(3)}\right) = (50, 50, 50)$ for $M = 3$, respectively, while setting $\boldsymbol{p} = (\lfloor 3\rho \rfloor, \lfloor 4\rho \rfloor, \lfloor 5\rho \rfloor, \lfloor 6\rho \rfloor, \lfloor 7\rho \rfloor)^\top$ with $\rho = 4$.

To generate distinct mean patterns, we follow the population mean designs (Benjamini and Hochberg, 1995; Chen and Qin, 2010). For $M = 1$, let $\boldsymbol{\mu}_0 = \mathbf{0}_{p\times 1}$ denote the fixed hypothesized mean. We specify the components of $\boldsymbol{\mu}^{(1)} \triangleq \left(\mu_1^{(1)}, \ldots, \mu_p^{(1)}\right)^\top$ as follows. Let $\ell \in \{0.00, 0.10, 0.20, 0.50, 0.80, 0.90, 1.00\}$ represent the proportion of true null hypotheses among all p null hypotheses. At a fixed value of ℓ, three configurations are considered for the false null hypotheses, i.e., $\{\mu_{j_l}^{(1)} \neq 0 : j_l \in \{1, \ldots, p\}, 1 \leq l \leq \lfloor (1-\ell)p \rfloor\}$ if $\lfloor (1-\ell)p \rfloor \geq 1$ and $\varnothing$ if otherwise. These configurations include the equal configuration, where $\mu_{j_1}^{(1)} = \cdots = \mu_{j_{\lfloor (1-\ell)p \rfloor}}^{(1)} = L$; the linearly increasing configuration, where $\mu_{j_1}^{(1)} = L/(\lfloor (1-\ell)p \rfloor), \mu_{j_2}^{(1)} = 2L/(\lfloor (1-\ell)p \rfloor), \ldots, \mu_{j_{\lfloor (1-\ell)p \rfloor}}^{(1)} = L$; and the linear decreasing configuration, where $\mu_{j_1}^{(1)} = -L/(\lfloor (1-\ell)p \rfloor), \mu_{j_2}^{(1)} = -2L/(\lfloor (1-\ell)p \rfloor), \ldots, \mu_{j_{\lfloor (1-\ell)p \rfloor}}^{(1)} = -L$. Here, L is determined by solving $\|\boldsymbol{\mu}^{(1)} - \boldsymbol{\mu}_0\|^2 / \operatorname{tr}^{1/2}(\boldsymbol{\Sigma}_\epsilon^{(1),2}) = 0.02$, where $\boldsymbol{\Sigma}_\epsilon^{(1)}$ is the population covariance matrix discussed later. For $M = 3$, we set $\boldsymbol{\mu}^{(2)} = 0.75 \times \boldsymbol{\mu}^{(1)}$ and $\boldsymbol{\mu}^{(3)} = 0.50 \times \boldsymbol{\mu}^{(1)}$. Now L is determined by solving $\|\boldsymbol{\mu}^{(1)} - \boldsymbol{\mu}_0\|^2 / \operatorname{tr}^{1/2}(\boldsymbol{\Sigma}_\epsilon^{(1),2}) = 0.5$.

To evaluate robustness against random noise, we define $\boldsymbol{\Sigma}_\epsilon^{(m)} \triangleq \boldsymbol{\Sigma}^{(m)}\left[\mathbf{A}^{(m)}, \mathbf{B}^{(m)}, \boldsymbol{p}\right] + \mathbf{E}_\epsilon$, where $m = 1$ for $M = 1$ and $m \in \{1, 2, 3\}$ for $M = 3$. The matrices $\mathbf{A}^{(m)} = \mathbf{A}_*$ and $\mathbf{B}^{(m)} = \mathbf{B}_*$ are the same as in Study 1, while $\mathbf{E}_\epsilon \sim W_p(\epsilon \mathbf{I}_p, p)$ represents random noise at levels $\epsilon \in \{0.00, 0.01, 0.05\}$. When $\epsilon = 0$, $\boldsymbol{\Sigma}_\epsilon^{(m)}$ reduces to the uniform-block matrix in Study 1. Using Monte Carlo simulations with 10^4 replicates, we compute the empirical statistical power and size for every mean pattern and every value of ϵ. Specifically, for each replicate, we generate a random sample from $N_p\left(\boldsymbol{\mu}^{(m)}, \boldsymbol{\Sigma}_\epsilon^{(m)}\right)$, compute the test statistics, and compare them with the theoretical critical values (i.e., $(1-\alpha)$th percentiles of theoretical distributions) at a predetermined significance level of $\alpha = 0.05$. Rejections are counted when the test statistic exceeds the critical value. The empirical statistical powers (when $\ell \neq 1$) and sizes (when $\ell = 1$) of the proposed joint statistics are reported in Table A.4.

To assess robustness against random missingness, we focus on the population covariance matrices $\boldsymbol{\Sigma}_\epsilon^{(m)}$ with $\epsilon = 0$, considering $m = 1$ for $M = 1$ and $m \in \{1, 2, 3\}$ for $M = 3$. Monte Carlo simulations with 10^4 replicates are conducted to evaluate empirical statistical powers and sizes under different levels of missingness in the sample covariance matrices. Specifically, for

each replicate, we generate a random sample from $N_p\left(\boldsymbol{\mu}^{(m)}, \boldsymbol{\Sigma}_\epsilon^{(m)}\right)$ with $\epsilon = 0$, then introduce a proportion of $\{0.00, 0.05, 0.10, 0.15\}$ of missing values in $\mathbf{S}^{(m)}$ for $m \in \{1, \ldots, M\}$. Without imputing the missing values, we compute the test statistics G_2 and G_4, counting rejections when the test statistic exceeds the critical value. The results for empirical statistical powers (when $\ell \neq 1$) and sizes (when $\ell = 1$) of the proposed G_2 and G_4 are summarized in Table A.5.

Similar to the random missingness scenario, we remove the missingness design and instead sample from a multivariate t-distribution rather than a multivariate normal distribution. The mean vectors and covariance matrices of the multivariate normal distributions are used as the location vectors and scale matrices for the corresponding multivariate t-distribution. In addition, the degrees of freedom parameters are chosen from $\{1, 3, 5, 10, \infty\}$ for each fixed configuration of the location vector and scale matrix. The results are summarized in Table A.6 (a complete result was provided in the online Excel file).

The simulation results indicate that when the uniform-block covariance structure is slightly or moderately disrupted, the proposed test statistics experience only modest reductions in empirical powers and slight deviations in sizes from the nominal level. Furthermore, even with moderate levels of random missingness in the sample covariance structure, the proposed test statistics maintain reliable empirical performance. Under heavy-tailed distributions, the proposed LRT and Geisser's information statistics display only minor deviations in empirical power and size when the degrees of freedom parameters are moderately large. In contrast, for small degrees of freedom parameters, both statistics exhibit moderate bias in empirical power and size compared with the multivariate normal setting. These findings suggest that our tests exhibit robustness in hypothesis testing by effectively leveraging the covariance structure.

Table A.4: Results of empirical powers (proportion $l \neq 1$) and sizes (proportion $l = 1$) under random noise that disrupts the uniform-block structures, where "Proportion" represents the proportion of true null hypotheses among all hypotheses, Λ and G refer to either Λ_2, G_2 or Λ_4, G_4, and "ϵ" represents the noise level

Population	Pattern	Proportion	$\epsilon = 0\%$			$\epsilon = 1\%$			$\epsilon = 5\%$		
			$-\log \Lambda$ (beta)	$-\log \Lambda$ (F)	G	$-\log \Lambda$ (beta)	$-\log \Lambda$ (F)	G	$-\log \Lambda$ (beta)	$-\log \Lambda$ (F)	G
$M = 1$	equal	0%	0.108	0.120	0.142	0.134	0.144	0.153	0.136	0.146	0.151
		10%	1.000	1.000	1.000	0.258	0.273	0.282	0.160	0.171	0.177
		20%	1.000	1.000	1.000	0.256	0.272	0.280	0.163	0.172	0.178
		50%	0.997	0.997	0.998	0.189	0.201	0.209	0.144	0.156	0.162
		80%	1.000	1.000	1.000	0.806	0.820	0.875	0.273	0.287	0.317
		90%	1.000	1.000	1.000	0.769	0.783	0.808	0.234	0.251	0.261
		100%	0.047	0.054	0.055	0.100	0.108	0.107	0.112	0.120	0.120
	increasing	0%	0.830	0.845	0.905	0.249	0.266	0.318	0.158	0.167	0.179
		10%	1.000	1.000	1.000	0.646	0.664	0.695	0.222	0.235	0.244
		20%	1.000	1.000	1.000	0.676	0.694	0.719	0.231	0.245	0.257
		50%	1.000	1.000	1.000	0.498	0.517	0.551	0.196	0.208	0.218
		80%	1.000	1.000	1.000	0.992	0.994	0.997	0.400	0.419	0.446
		90%	1.000	1.000	1.000	0.956	0.961	0.969	0.300	0.315	0.322
		100%	0.047	0.054	0.055	0.100	0.108	0.107	0.112	0.120	0.120
	decreasing	0%	0.838	0.850	0.908	0.247	0.264	0.314	0.158	0.168	0.181
		10%	1.000	1.000	1.000	0.641	0.660	0.694	0.219	0.231	0.244
		20%	1.000	1.000	1.000	0.678	0.695	0.721	0.228	0.243	0.255
		50%	1.000	1.000	1.000	0.493	0.515	0.547	0.196	0.209	0.220
		80%	1.000	1.000	1.000	0.990	0.991	0.994	0.402	0.422	0.446
		90%	1.000	1.000	1.000	0.952	0.958	0.967	0.294	0.311	0.319
		100%	0.047	0.054	0.055	0.100	0.108	0.107	0.112	0.120	0.120
$M = 3$	equal	0%	0.150	0.158	0.169	0.157	0.165	0.167	0.151	0.157	0.156
		10%	1.000	1.000	1.000	0.385	0.396	0.396	0.191	0.198	0.197
		20%	1.000	1.000	1.000	0.378	0.388	0.388	0.195	0.203	0.202
		50%	1.000	1.000	1.000	0.256	0.266	0.264	0.166	0.174	0.173
		80%	1.000	1.000	1.000	0.981	0.982	0.988	0.412	0.424	0.440
		90%	1.000	1.000	1.000	0.975	0.978	0.979	0.336	0.348	0.348
		100%	0.048	0.051	0.049	0.098	0.102	0.099	0.108	0.113	0.111
	increasing	0%	0.990	0.992	0.996	0.376	0.389	0.428	0.194	0.204	0.206
		10%	1.000	1.000	1.000	0.907	0.913	0.921	0.304	0.315	0.316
		20%	1.000	1.000	1.000	0.934	0.939	0.943	0.319	0.329	0.330
		50%	1.000	1.000	1.000	0.773	0.783	0.794	0.268	0.278	0.278
		80%	1.000	1.000	1.000	1.000	1.000	1.000	0.625	0.638	0.648
		90%	1.000	1.000	1.000	1.000	1.000	1.000	0.446	0.458	0.457
		100%	0.048	0.051	0.049	0.098	0.102	0.099	0.108	0.113	0.111
	decreasing	0%	0.990	0.991	0.995	0.376	0.388	0.423	0.195	0.202	0.204
		10%	1.000	1.000	1.000	0.905	0.909	0.917	0.305	0.317	0.318
		20%	1.000	1.000	1.000	0.927	0.931	0.935	0.325	0.335	0.338
		50%	1.000	1.000	1.000	0.770	0.780	0.790	0.256	0.266	0.267
		80%	1.000	1.000	1.000	1.000	1.000	1.000	0.627	0.637	0.647
		90%	1.000	1.000	1.000	1.000	1.000	1.000	0.445	0.457	0.457
		100%	0.048	0.051	0.049	0.098	0.102	0.099	0.108	0.113	0.111

Table A.5: Results of empirical powers (proportion $l \neq 1$) and sizes (proportion $l = 1$) under random missingness that results in incomplete sample covariance matrices, where "Proportion" represents the proportion of true null hypotheses among all hypotheses, and "miss" represents the proportion of missing values in the sample covariance matrices

Pattern	Proportion	$M = 1, G_2$				$M = 3, G_4$			
		miss 0%	miss 5%	miss 10%	miss 15%	miss 0%	miss 5%	miss 10%	miss 15%
equal	0%	0.139	0.212	0.278	0.337	0.162	0.201	0.249	0.284
	10%	1.000	1.000	1.000	1.000	1.000	1.000	1.000	1.000
	20%	1.000	1.000	1.000	1.000	1.000	1.000	1.000	1.000
	50%	0.998	0.995	0.993	0.989	1.000	1.000	1.000	1.000
	80%	1.000	1.000	1.000	1.000	1.000	1.000	1.000	1.000
	90%	1.000	1.000	1.000	1.000	1.000	1.000	1.000	1.000
	100%	0.057	0.130	0.195	0.256	0.050	0.080	0.121	0.157
increasing	0%	0.902	0.903	0.903	0.907	0.995	0.995	0.992	0.991
	10%	1.000	1.000	1.000	1.000	1.000	1.000	1.000	1.000
	20%	1.000	1.000	1.000	1.000	1.000	1.000	1.000	1.000
	50%	1.000	1.000	1.000	1.000	1.000	1.000	1.000	1.000
	80%	1.000	1.000	1.000	1.000	1.000	1.000	1.000	1.000
	90%	1.000	1.000	1.000	1.000	1.000	1.000	1.000	1.000
	100%	0.057	0.130	0.195	0.256	0.050	0.080	0.121	0.157
decreasing	0%	0.905	0.910	0.909	0.911	0.994	0.993	0.993	0.992
	10%	1.000	1.000	1.000	1.000	1.000	1.000	1.000	1.000
	20%	1.000	1.000	1.000	1.000	1.000	1.000	1.000	1.000
	50%	1.000	1.000	1.000	1.000	1.000	1.000	1.000	1.000
	80%	1.000	1.000	1.000	1.000	1.000	1.000	1.000	1.000
	90%	1.000	1.000	1.000	1.000	1.000	1.000	1.000	1.000
	100%	0.057	0.130	0.195	0.256	0.050	0.080	0.121	0.157

Table A.6: Results of empirical powers (proportion $l \neq 1$) and sizes (proportion $l = 1$) under distributional misspecification that violates the normality assumption, where "Proportion" represents the proportion of true null hypotheses among all hypotheses, and "df" represents the degrees of freedom parameter of the multivariate t-distributions

Pattern	Proportion	$M = 1, G_2$					$M = 3, G_4$				
		df 1	df 3	df 5	df 10	df Inf (normal)	df 1	df 3	df 5	df 10	df Inf (normal)
equal	0%	0.019	0.084	0.098	0.124	0.142	0.018	0.085	0.110	0.138	0.169
	10%	0.040	0.803	0.972	0.999	1.000	0.024	0.948	1.000	1.000	1.000
	20%	0.039	0.800	0.971	0.998	1.000	0.024	0.944	1.000	1.000	1.000
	50%	0.034	0.665	0.907	0.979	0.998	0.021	0.871	0.994	1.000	1.000
	80%	0.118	0.982	1.000	1.000	1.000	0.046	0.998	1.000	1.000	1.000
	90%	0.500	1.000	1.000	1.000	1.000	0.342	1.000	1.000	1.000	1.000
	100%	0.016	0.047	0.049	0.055	0.055	0.017	0.046	0.050	0.049	0.049
increasing	0%	0.041	0.468	0.666	0.814	0.905	0.023	0.619	0.875	0.972	0.996
	10%	0.127	0.992	1.000	1.000	1.000	0.053	0.999	1.000	1.000	1.000
	20%	0.136	0.994	1.000	1.000	1.000	0.055	0.999	1.000	1.000	1.000
	50%	0.117	0.991	1.000	1.000	1.000	0.048	0.999	1.000	1.000	1.000
	80%	0.337	1.000	1.000	1.000	1.000	0.170	1.000	1.000	1.000	1.000
	90%	0.590	1.000	1.000	1.000	1.000	0.442	1.000	1.000	1.000	1.000
	100%	0.016	0.047	0.049	0.055	0.055	0.017	0.046	0.050	0.049	0.049
decreasing	0%	0.039	0.466	0.663	0.803	0.908	0.023	0.620	0.884	0.971	0.995
	10%	0.118	0.991	1.000	1.000	1.000	0.052	0.999	1.000	1.000	1.000
	20%	0.127	0.994	1.000	1.000	1.000	0.056	0.999	1.000	1.000	1.000
	50%	0.112	0.991	1.000	1.000	1.000	0.053	0.999	1.000	1.000	1.000
	80%	0.337	1.000	1.000	1.000	1.000	0.170	1.000	1.000	1.000	1.000
	90%	0.594	1.000	1.000	1.000	1.000	0.451	1.000	1.000	1.000	1.000
	100%	0.016	0.047	0.049	0.055	0.055	0.017	0.046	0.050	0.049	0.049

B REAL DATA ANALYSIS

Our motivating dataset originates from a high-dimensional echo-planar spectroscopic imaging (EPSI) study. In previous research, Chiappelli et al. (2019) investigated the associations between regional neurometabolite levels and cardiovascular risk factors, suggesting that neurometabolite levels could serve as early biomarkers for cognitive decline. They employed linear regression models to estimate the associations between Framingham cardiovascular risk scores (D'Agostino et al., 2008) and regional neurometabolite levels, adjusting for covariates such as gender and age due to their potential relationships with neurometabolites.

Objectives. Leveraging the study protocol and data (Chiappelli et al., 2019), the current analysis aimed to examine the interdependence of regional neurometabolite levels and to validate the influence of gender and age on these levels. Our data analysis evaluated model specifications and provided statistical insights for future research.

Covariance structure. We explored the relationships between measurements from 89 brain regions (see the regions in the online Excel file) and 5 neurometabolites: choline-containing compounds (Cho), creatine-containing compounds (Cre), glutamate and glutamine (Glx), myo-inositol (Mi), and N-acetylaspartate (NAA). As illustrated in Figure B.1, the correlation matrix of the regional neurometabolite levels naturally exhibited a uniform-block structure, where each block represented the levels of a single neurometabolite across 89 brain regions. This motivated our use of the proposed approach to determine the dependence relationships between regional neurometabolites and to test whether their covariance structures and mean levels were stratified by gender and age, considering the uniform-block covariance structures.

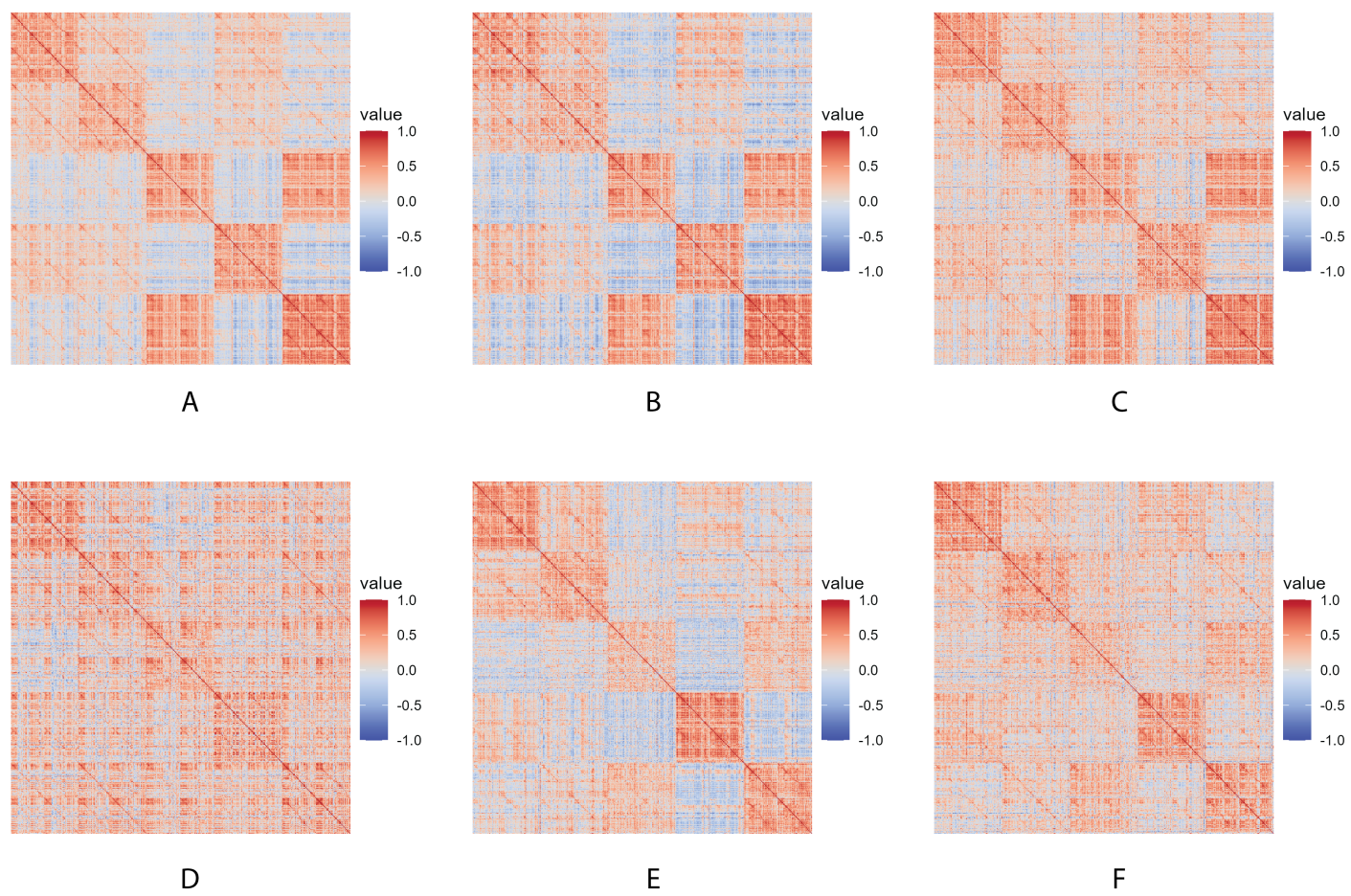

Figure B.1: Heatmaps (445 by 445) exhibit correlation matrices for all participants (A), female participants (B), male participants (C), participants aged 12–30 (D), participants aged 31–50 (E), participants aged 51–77 (F) using "pair" method, where (diagonally from top to bottom) blocks represent choline-containing compounds (Cho), creatine-containing compounds (Cre), glutamate and glutamine (Glx), myo-inositol(Mi), and N-acetylaspartate (NAA), respectively.

Data analysis for all participants. The EPSI dataset consisted of $n = n^{(1)} = 78$ participants, with an average age of 42.1 and a standard deviation of 18.8. Each participant had $p = 445$ measurements, i.e., $\boldsymbol{X}_i^{(m)} \in \mathbb{R}^p$ with $m = M = 1$, representing combinations of $K = 5$ neurometabolites across $p_k = 89$ brain regions for $k \in \{1, \ldots, K\}$. The sample correlation matrices of all participants, as shown in Figure B.1, displayed the structure of uniform blocks. Two primary challenges existed for classical hypothesis testing. The high-dimensionality issue: $n = 78 < p = 445$, and the presence of missing values: calculating the sample covariance matrix $\mathbf{S}^{(1)}$ revealed that 76.65% of its entries were missing. To address the high-dimensionality issue, we considered the proposed LRT statistics and Geisser's information statistics rather than the classical Hotelling's T^2 statistics. To address the missing data, we employed three approaches. The "full" method: calculate $\mathbf{S}^{(m)}$ using all entries, including those with missing values in $\boldsymbol{X}_i^{(m)}$. The "pair(wise)" method: calculate $\mathbf{S}^{(m)}$ based on all complete pairs of variables, excluding any observations with missing values. The "impu(ted)" method: impute missing observations in $\boldsymbol{X}_i^{(m)}$, for all i and m, using a numerical approach (Stekhoven and Bühlmann, 2011) and then calculate $\mathbf{S}^{(m)}$. Thus, the "pair" and "impu" methods resulted in $\mathbf{S}^{(m)}$ matrices without missing values.

Given an empirical center 1.645, i.e., the average of components in $\bar{\boldsymbol{X}}$, we tested the hypoth-

esis that the population mean vector has a homogeneous structure: $\boldsymbol{\mu}^{(1)} = 1.645 \times \mathbf{1}_{p\times 1}$, using the proposed Λ_2, G_2, and $\mathbf{S}^{(1)}$ matrices obtained from “full”, “pair”, and “impu” methods, respectively. The estimates and testing results were detailed in Tables B.1 and B.2. Under the “full” framework, only G_2 was available due to the extensive missing data, and it suggested statistically rejecting the above hypothesis due to the extremely small p-value (i.e., $< 10^{-4}$). Under the “pair” and “impu” frameworks, both Λ_2 and G_2 were available, and they also indicated rejection of the homogeneous mean hypothesis due to the extremely small p-values (i.e., $< 10^{-4}$).

Additionally, we tested whether the marginal mean components were simultaneously equal to the empirical center: $\mu_j^{(1)} = 1.645$ for $j \in \{1, \dots, 445\}$, using the proposed FDP estimation procedure. The results for marginal mean components were provided in the online Excel file. The adjusted p-values for the marginal tests and the corresponding t_α values were reported for the “full”, “pair”, and “impu” methods, with the respective estimated FDPs of 0.0492, 0.0489, and 0.0499. At a significance level of 0.05, the regional neurometabolites whose marginal mean levels were statistically different from the empirical center of 1.645 were also reported for “full”, “pair”, and “impu” methods.

Table B.1: Estimation results for data analysis for all participants

	“full” method					“pair” method					“impu” method				
	0.0015					0.0026					0.0024				
		0.0181					0.0324					0.0289			
$\widehat{\mathbf{A}}^{(1)}$			0.0334					0.0573					0.0517		
				0.0318					0.0581					0.0511	
					0.0277					0.0395					0.0362
	0.0015	0.0027	-0.0005	0.0040	0.0000	0.0014	0.0021	-0.0003	0.0029	0.0003	0.0013	0.0021	-0.0001	0.0029	0.0004
	0.0027	0.0145	0.0023	0.0110	0.0020	0.0021	0.0119	0.0017	0.0062	0.0021	0.0021	0.0118	0.0020	0.0065	0.0024
$\widehat{\mathbf{B}}^{(1)}$	-0.0005	0.0023	0.0363	-0.0007	0.0356	-0.0003	0.0017	0.0266	-0.0046	0.0288	-0.0001	0.0020	0.0263	-0.0037	0.0286
	0.0040	0.0110	-0.0007	0.0317	-0.0021	0.0029	0.0062	-0.0046	0.0251	-0.0068	0.0029	0.0065	-0.0037	0.0247	-0.0056
	0.0000	0.0020	0.0356	-0.0021	0.0446	0.0003	0.0021	0.0288	-0.0068	0.0417	0.0004	0.0024	0.0286	-0.0056	0.0407

Table B.2: Statistical decision results for data analysis for all participants

	“full” method				“pair” method				“impu” method			
	$-\log \Lambda_1$	$-\log \Lambda_2$ (beta)	$-\log \Lambda_2$ (F)	G_2	$-\log \Lambda_1$	$-\log \Lambda_2$ (beta)	$-\log \Lambda_2$ (F)	G_2	$-\log \Lambda_1$	$-\log \Lambda_2$ (beta)	$-\log \Lambda_2$ (F)	G_2
test statistic	NA	NA	NA	310365	Inf	21574	21574	239954	Inf	22842	22842	259072
critical point	Inf	249	249	496	Inf	249	249	496	Inf	249	249	496
rejection	NA	NA	NA	yes	NA	yes	yes	yes	NA	yes	yes	yes
p-value	NA	NA	NA	0	NA	0	0	0	NA	0	0	0

Data analysis for the gender-specific participants. We then investigated the influence of gender. With $M = 2$, the EPSI dataset comprised an equal number of male and female participants, e.g., $n^{(1)} = n^{(2)} = 39$. The sample correlation matrices for male and female partici-

pants, as depicted in Figure B.1, exhibited the structures of uniform blocks, sharing a common $\boldsymbol{p} = (p_1, \ldots, p_5)^\top$ with $K = 5$ and $p_k = 89$ for $k \in \{1, \ldots, K\}$. Missing values in $\boldsymbol{X}_i^{(m)}$ were handled separately for each gender group using the "full", "pair", and "impu" methods, respectively.

We tested the equality of uniform-block covariance structures using the proposed Λ_3. The estimated population covariance matrices were available in the online Excel file. Testing results of Λ_3 suggested a statistical difference between gender-specific covariance structures at a significance level of 0.05 for both "pair" and "impu" methods. Additionally, we tested the equality of population mean vectors using the proposed Λ_4 and G_4. The estimates and testing were provided in Tables B.3 and B.4. The testing results indicated significant heterogeneity in mean vectors at the 0.05 level.

Table B.3: Estimation results for data analysis for the gender-specific participants

			"full" method							
			Male					Female		
	0.0014					0.0020				
		0.0190					0.0219			
$\widehat{\mathbf{A}}^{(m)}$			0.0366					0.0377		
				0.0442					0.0362	
					0.0287					0.0306
	0.0015	0.0025	0.0011	0.0046	0.0014	0.0015	0.0030	-0.0022	0.0035	-0.0016
	0.0025	0.0158	0.0091	0.0129	0.0086	0.0030	0.0120	-0.0032	0.0063	-0.0035
$\widehat{\mathbf{B}}^{(m)}$	0.0011	0.0091	0.0371	0.0102	0.0382	-0.0022	-0.0032	0.0322	-0.0118	0.0309
	0.0046	0.0129	0.0102	0.0368	0.0078	0.0035	0.0063	-0.0118	0.0260	-0.0135
	0.0014	0.0086	0.0382	0.0078	0.0432	-0.0016	-0.0035	0.0309	-0.0135	0.0452
			"pair" method							
			Male					Female		
	0.0026					0.0026				
		0.0326					0.0306			
$\widehat{\mathbf{A}}^{(m)}$			0.0589					0.0540		
				0.0619					0.0547	
					0.0393					0.0391
	0.0014	0.0020	0.0009	0.0032	0.0014	0.0014	0.0023	-0.0018	0.0031	-0.0011
	0.0020	0.0141	0.0064	0.0080	0.0081	0.0023	0.0097	-0.0022	0.0035	-0.0031
$\widehat{\mathbf{B}}^{(m)}$	0.0009	0.0064	0.0256	0.0035	0.0296	-0.0018	-0.0022	0.0275	-0.0111	0.0278
	0.0032	0.0080	0.0035	0.0248	0.0023	0.0031	0.0035	-0.0111	0.0235	-0.0138
	0.0014	0.0081	0.0296	0.0023	0.0381	-0.0011	-0.0031	0.0278	-0.0138	0.0446
			"impu" method							
			Male					Female		
	0.0023					0.0024				
		0.0286					0.0277			
$\widehat{\mathbf{A}}^{(m)}$			0.0522					0.0497		
				0.0559					0.0466	
					0.0346					0.0368
	0.0014	0.0021	0.0013	0.0033	0.0017	0.0013	0.0023	-0.0017	0.0030	-0.0012
	0.0021	0.0137	0.0071	0.0082	0.0087	0.0023	0.0095	-0.0022	0.0038	-0.0030
$\widehat{\mathbf{B}}^{(m)}$	0.0013	0.0071	0.0257	0.0049	0.0299	-0.0017	-0.0022	0.0266	-0.0108	0.0269
	0.0033	0.0082	0.0049	0.0249	0.0036	0.0030	0.0038	-0.0108	0.0229	-0.0134
	0.0017	0.0087	0.0299	0.0036	0.0388	-0.0012	-0.0030	0.0269	-0.0134	0.0426

Table B.4: Statistical decision results for data analysis for the gender-specific participants

	"full" method				"pair" method				"impu" method			
	$-\log\Lambda_3$	$-\log\Lambda_4$ (beta)	$-\log\Lambda_4$ (F)	G_4	$-\log\Lambda_3$	$-\log\Lambda_4$ (beta)	$-\log\Lambda_4$ (F)	G_4	$-\log\Lambda_3$	$-\log\Lambda_4$ (beta)	$-\log\Lambda_4$ (F)	G_4
test statistic	NA	NA	NA	1712	28	510	510	1011	36	567	567	1126
critical point	17	253	253	496	17	253	253	496	17	253	253	496
rejection	NA	NA	NA	yes	yes	yes	yes	yes	yes	yes	yes	yes
p-value	NA	NA	NA	0	0	0	0	0	0	0	0	0

Data analysis for the age-specific participants. With $M = 3$, we considered three age-specific groups (Lecoeur et al., 2022): $n^{(1)} = 24$ participants aged 12–30, $n^{(2)} = 27$ participants aged 31–50, and $n^{(3)} = 27$ participants aged 51–77. Other age group definitions were possible. The sample correlation matrices for these age groups, as depicted in Figure B.1, exhibited the structures of uniform blocks, sharing a common $\boldsymbol{p} = (p_1, \ldots, p_5)^\top$ with $K = 5$ and $p_k = 89$ for $k \in \{1, \ldots, K\}$. We conducted hypothesis tests for the equality of population uniform-block covariance structures and mean vectors across age-specified groups using the proposed Λ_3, Λ_4, G_4 with the "full", "pair", and "impu" methods. Following these imputation methods, we calculated the estimates of population covariance matrices. The estimates and testing were provided in Tables B.5 and B.6. The testing results demonstrated statistically significant heterogeneity in both population covariance and mean structures at the 0.05 level.

Table B.5: Estimation results for data analysis for the age-specific participants

	“full” method														
	aged 12-30					aged 31-50					aged 51-77				
	0.0018					0.0014					0.0019				
		0.0214					0.0174					0.0260			
$\widehat{\mathbf{A}}^{(m)}$			0.0463					0.0351					0.0418		
				0.0534					0.0358					0.0388	
					0.0298					0.0225					0.0345
	0.0017	0.0034	0.0020	0.0052	0.0040	0.0011	0.0014	-0.0006	0.0019	0.0002	0.0017	0.0023	0.0015	0.0033	0.0008
	0.0034	0.0188	0.0158	0.0175	0.0169	0.0014	0.0083	-0.0001	0.0014	0.0031	0.0023	0.0141	0.0078	0.0056	0.0068
$\widehat{\mathbf{B}}^{(m)}$	0.0020	0.0158	0.0299	0.0182	0.0230	-0.0006	-0.0001	0.0107	-0.0060	0.0084	0.0015	0.0078	0.0134	0.0065	0.0141
	0.0052	0.0175	0.0182	0.0353	0.0186	0.0019	0.0014	-0.0060	0.0256	-0.0047	0.0033	0.0056	0.0065	0.0245	0.0041
	0.0040	0.0169	0.0230	0.0186	0.0269	0.0002	0.0031	0.0084	-0.0047	0.0141	0.0008	0.0068	0.0141	0.0041	0.0224
	“pair” method														
	aged 12-30					aged 31-50					aged 51-77				
	0.0028					0.0019					0.0026				
		0.0349					0.0257					0.0341			
$\widehat{\mathbf{A}}^{(m)}$			0.0621					0.0499					0.0560		
				0.0596					0.0593					0.0496	
					0.0388					0.0280					0.0437
	0.0015	0.0021	0.0005	0.0028	0.0025	0.0012	0.0018	-0.0005	0.0018	0.0007	0.0015	0.0020	0.0011	0.0032	0.0007
	0.0021	0.0116	0.0087	0.0074	0.0104	0.0018	0.0095	0.0011	0.0009	0.0052	0.0020	0.0130	0.0063	0.0057	0.0054
$\widehat{\mathbf{B}}^{(m)}$	0.0005	0.0087	0.0203	0.0092	0.0127	-0.0005	0.0011	0.0101	-0.0067	0.0089	0.0011	0.0063	0.0111	0.0050	0.0125
	0.0028	0.0074	0.0092	0.0186	0.0099	0.0018	0.0009	-0.0067	0.0250	-0.0045	0.0032	0.0057	0.0050	0.0216	0.0040
	0.0025	0.0104	0.0127	0.0099	0.0155	0.0007	0.0052	0.0089	-0.0045	0.0161	0.0007	0.0054	0.0125	0.0040	0.0192
	“impu” method														
	aged 12-30					aged 31-50					aged 51-77				
	0.0026					0.0018					0.0023				
		0.0312					0.0229					0.0300			
$\widehat{\mathbf{A}}^{(m)}$			0.0577					0.0435					0.0491		
				0.0545					0.0454					0.0443	
					0.0358					0.0257					0.0395
	0.0015	0.0023	0.0006	0.0034	0.0025	0.0011	0.0017	-0.0004	0.0018	0.0006	0.0015	0.0021	0.0013	0.0030	0.0009
	0.0023	0.0125	0.0089	0.0094	0.0105	0.0017	0.0090	0.0010	0.0014	0.0047	0.0021	0.0126	0.0065	0.0051	0.0062
$\widehat{\mathbf{B}}^{(m)}$	0.0006	0.0089	0.0195	0.0097	0.0125	-0.0004	0.0010	0.0100	-0.0060	0.0085	0.0013	0.0065	0.0110	0.0046	0.0123
	0.0034	0.0094	0.0097	0.0218	0.0103	0.0018	0.0014	-0.0060	0.0242	-0.0038	0.0030	0.0051	0.0046	0.0211	0.0033
	0.0025	0.0105	0.0125	0.0103	0.0152	0.0006	0.0047	0.0085	-0.0038	0.0150	0.0009	0.0062	0.0123	0.0033	0.0191

Table B.6: Statistical decision results for data analysis of the age-specific participants

	“full” method				“pair” method				“impu” method			
	$-\log\Lambda_3$	$-\log\Lambda_4$ (beta)	$-\log\Lambda_4$ (F)	G_4	$-\log\Lambda_3$	$-\log\Lambda_4$ (beta)	$-\log\Lambda_4$ (F)	G_4	$-\log\Lambda_3$	$-\log\Lambda_4$ (beta)	$-\log\Lambda_4$ (F)	G_4
test statistic	NA	NA	NA	5037	181	1472	1472	3053	184	1641	1641	3417
critical point	31	493	493	963	31	493	493	963	31	493	493	963
rejection	NA	NA	NA	yes	yes	yes	yes	yes	yes	yes	yes	yes
p-value	NA	NA	NA	0	0	0	0	0	0	0	0	0

Results. In contrast to conventional Hotelling-type statistics, we have demonstrated the advantages of the proposed LRT and Geisser’s information statistics in handling high-dimensionality and missing data. The proposed estimators produced reliable estimates of population parameters compared to imputation methods, but with reduced computational time. For all participants, the off-diagonal elements of the estimated $\mathbf{B}^{(1)}$ represented the estimated covariances between regional neurometabolite levels. We observed relatively strong positive dependence between Cre

and Mi (glia-related metabolites), and between Glx and NAA (neuron-related metabolites), probably due to their metabolic interconnections (Clark et al., 2006; Lind et al., 2020; Bissonnette et al., 2022). Using the gender- or age-specific participants, the results consistently indicated that both uniform-block covariance structures and mean vectors were not identical, as evidenced by extremely small p-values at a significance level of 0.05. These differences in gender- or age-specific participants highlighted the necessity of stratification in subsequent analyses. These findings suggested that future research could achieve more precise statistical inferences by incorporating the interconnected relationship between neurometabolites and adjusting for gender and age.

References

Benjamini, Y. and Hochberg, Y. (1995). Controlling the false discovery rate: a practical and powerful approach to multiple testing. *J. Roy. Statist. Soc. Ser. B* **57,** 289–300.

Bissonnette, J., Francis, A., MacNeil, S., Crocker, C., Tibbo, P., and Fisher, D. (2022). Glutamate and N-acetylaspartate alterations observed in early phase psychosis: A systematic review of proton magnetic resonance spectroscopy studies. *Psychiatry Res. Neuroimaging* **321,** 111459.

Chen, S. X. and Qin, Y.-L. (2010). A two-sample test for high-dimensional data with applications to gene-set testing. *Ann. Statist.* **38,** 808–835.

Chiappelli, J., Rowland, L. M., Wijtenburg, S. A., Chen, H., Maudsley, A. A., Sheriff, S., Chen, S., Savransky, A., Marshall, W., Ryan, M. C., Bruce, H. A., Shuldiner, A. R., Mitchell, B. D., Kochunov, P., and Hong, L. E. (2019). Cardiovascular risks impact human brain N-acetylaspartate in regionally specific patterns. *Proc. Natl. Acad. Sci.* **116,** 25243–25249.

Clark, J. F., Doepke, A., Filosa, J. A., Wardle, R. L., Lu, A., Meeker, T. J., and Pyne-Geithman, G. J. (2006). N-acetylaspartate as a reservoir for glutamate. *Med. Hypotheses* **67,** 506–512.

D'Agostino, R. B., Vasan, R. S., Pencina, M. J., Wolf, P. A., Cobain, M., Massaro, J. M., and Kannel, W. B. (2008). General cardiovascular risk profile for use in primary care. *Circulation* **117,** 743–753.

Fan, J. and Han, X. (2017). Estimation of the false discovery proportion with unknown dependence. *J. R. Stat. Soc. Ser. B. Stat. Methodol.* **79,** 1143–1164.

Fan, J., Han, X., and Gu, W. (2012). Estimating false discovery proportion under arbitrary covariance dependence. *J. Amer. Statist. Assoc.* **107,** 1019–1035.

Fan, J., Liao, Y., and Mincheva, M. (2011). High-dimensional covariance matrix estimation in approximate factor models. *Ann. Statist.* **39,** 3320–3356.

Lecoeur, E., Domengé, O., Fayol, A., Jannot, A.-S., and Hulot, J.-S. (2022). Epidemiology of heart failure in young adults: a french nationwide cohort study. *Eur. Heart J.* **44,** 383–392.

Lind, A., Boraxbekk, C.-J., Petersen, E. T., Paulson, O. B., Siebner, H. R., and Marsman, A. (2020). Regional myo-inositol, creatine, and choline levels are higher at older age and scale negatively with visuospatial working memory: a cross-sectional proton MR spectroscopy study at 7 Tesla on normal cognitive ageing. *J. Neurosci.* **40,** 8149–8159.

Stekhoven, D. J. and Bühlmann, P. (2011). Missforest:non-parametric missing value imputation for mixed-type data. *Bioinformatics* **28,** 112–118.

Vutov, V. and Dickhaus, T. (2023a). multiple two-sample testing under arbitrary covariance dependency with an application in imaging mass spectrometry. *Biom. J.* **65,** Paper No. 2100328, 17.

Vutov, V. and Dickhaus, T. (2023b). multiple multi-sample testing under arbitrary covariance dependency. *Stat. Med.* **42,** 2944–2961.
